# Evolutionary origins of sensation in metazoans: functional evidence for a new sensory organ in sponges

**DOI:** 10.1186/1471-2148-14-3

**Published:** 2014-01-13

**Authors:** Danielle A Ludeman, Nathan Farrar, Ana Riesgo, Jordi Paps, Sally P Leys

**Affiliations:** 1 Department of Biological Sciences, University of Alberta, CW 405 Biological Sciences Building, Edmonton, Alberta T6G 2E9, Canada; 2 Department of Animal Biology, Universitat de Barcelona, Avinguda Diagonal 643, Barcelona 08028, Spain; 3 Department of Zoology, University of Oxford, Oxford OX1 3PS, UK

**Keywords:** Porifera, Primary cilia, Evolution of nervous systems, Sensory systems, PKD

## Abstract

**Background:**

One of the hallmarks of multicellular organisms is the ability of their cells to trigger responses to the environment in a coordinated manner. In recent years primary cilia have been shown to be present as ‘antennae’ on almost all animal cells, and are involved in cell-to-cell signaling in development and tissue homeostasis; how this sophisticated sensory system arose has been little-studied and its evolution is key to understanding how sensation arose in the Animal Kingdom. Sponges (Porifera), one of the earliest evolving phyla, lack conventional muscles and nerves and yet sense and respond to changes in their fluid environment. Here we demonstrate the presence of non-motile cilia in sponges and studied their role as flow sensors.

**Results:**

Demosponges excrete wastes from their body with a stereotypic series of whole-body contractions using a structure called the osculum to regulate the water-flow through the body. In this study we show that short cilia line the inner epithelium of the sponge osculum. Ultrastructure of the cilia shows an absence of a central pair of microtubules and high speed imaging shows they are non-motile, suggesting they are not involved in generating flow. In other animals non-motile, ‘primary’, cilia are involved in sensation. Here we show that molecules known to block cationic ion channels in primary cilia and which inhibit sensory function in other organisms reduce or eliminate sponge contractions. Removal of the cilia using chloral hydrate, or removal of the whole osculum, also stops the contractions; in all instances the effect is reversible, suggesting that the cilia are involved in sensation. An analysis of sponge transcriptomes shows the presence of several transient receptor potential (TRP) channels including PKD channels known to be involved in sensing changes in flow in other animals. Together these data suggest that cilia in sponge oscula are involved in flow sensation and coordination of simple behaviour.

**Conclusions:**

This is the first evidence of arrays of non-motile cilia in sponge oscula. Our findings provide support for the hypothesis that the cilia are sensory, and if true, the osculum may be considered a sensory organ that is used to coordinate whole animal responses in sponges. Arrays of primary cilia like these could represent the first step in the evolution of sensory and coordination systems in metazoans.

## Background

Sensory systems use specialized cells or organelles to receive signals that are conducted through the body electrically or chemically [1]. Signal transduction in many unicellular eukaryotes occurs via cilia, which often have both motile and sensory roles [2-4]. The evolution of multicellularity necessarily involved the ability to transduce signals over longer distances, which in animals is now done by nerves [5] to allow rapid coordinated movements of the whole organism [6]. Although cilia play an important role in sensing the environment in both unicellular and multicellular animals, the evolutionary relationship of sensory cilia in unicellular eukaryotes, fungi and metazoans is unclear. Studies of sensory systems in the earliest evolving metazoans could shed light on shared common mechanisms of sensation.

Sponges lack a nervous system and while they are usually considered representatives of the first multicellular animals [7-10], some recent phylogenomic analyses place ctenophores more basally [11,12] calling into question our understanding of the evolution of nerves and the ancestral metazoan state. Analysis of sponge genomes and transcriptomes has revealed a complex assortment of signaling molecules and proteins necessary for a post-synaptic scaffold [13,14]. Together with physiological evidence that glutamatergic signaling occurs in sponges [15,16] this suggests that a signaling system similar to that seen in other metazoans may be used to coordinate sponge behavior. Whereas sensory organs are well-known from ctenophores, in sponges the mechanism for transducing sensory information from the environment has as yet remained unknown.

Here we provide experimental data which suggest that an array of non-motile cilia in the sponge osculum–the chimney-like structure through which water exits from the sponge–functions as a sensory system to detect changes in flow and control whole animal responses. We used an emergent model system, the freshwater sponge, to investigate the ultrastructure and physiology of the cilia. We also studied the molecular evolution of sensory channels of the Transient Receptor Potential family in Porifera. Regardless of whether sponges as we know them today were or were not the earliest multicellular animals to evolve, it is intriguing to consider that an array of sensory cilia like this in sponge oscula could have given rise to more complex signalling cells, such as nerves and sensory sensilla, in the early evolution of animals.

## Results and discussion

### Sponge oscula are ciliated

**Figure 1:**
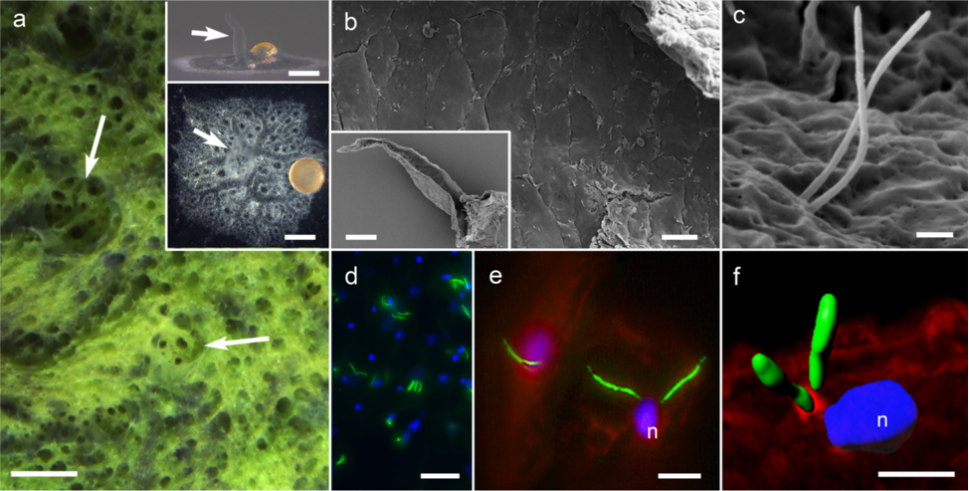
**Cilia are found on the epithelia lining the osculum*. *a**. The sponge *Ephydatia muelleri* in the lake, and grown in the lab viewed from the side (upper inset) and from above (lower inset). The oscula (white arrows) extend upwards from the body. **b**, **c**, Scanning electron micrographs show cilia arise from the middle of each cell along the entire length of the inside of the osculum; **b** the lining of the osculum with cilia on each cell (inset shows an osculum removed from the sponge and sliced in half longitudinally); **c**, two cilia arise from each cell. **d**, **e**, Cilia in the oscula labeled with antibodies to acetylated α-tubulin (green), nuclei with Hoechst (blue, n), actin with phalloidin (red). **f**. A 3D surface rendering illustrates how the cilia arise just above the nucleus of the cell. Scale bars **a** 5 mm; inset 1 mm; **b** 20 μm; inset 100 μm **c**, 1 μm **d**, 20 μm **e**, **f** 5 μm.

Sponges are unusual in possessing both cilia and flagella (named for their differing beat patterns [17]) on somatic cells. These include ciliated epithelial cells of sponge larvae which are involved in locomotion and also photoresponses [18,19], ciliated cells at the exit of the feeding choanocyte chambers [20,21] and flagellated choanocytes involved in pumping water through the canal system (reviewed in [20]). In contrast, the epithelia of adult sponges are usually naked. We were therefore surprised to find cilia on all cells forming the epithelial lining of the osculum in the freshwater sponge *Ephydatia muelleri*, a demosponge that can be cultured in the laboratory (Figure 1a). The osculum is the most prominent feature of a sponge, and is the final exit of water filtered through the sponge body for food and oxygen.

In *E. muelleri* a pair of cilia, each 4–6 microns long, emerges above the nucleus of every epithelial cell (Figure 1b-f). A survey of 6 other demosponges showed that in each, the oscula are also lined by ciliated cells; in some species the cells have a single cilium, and others up to 4 cilia, all arising centrally above the cell nucleus (Additional file 1: Figure S1). Even glass sponges (class Hexactinellida), which are syncytial, have cilia at the lip of their large oscula. There is no data available so far for the other two classes, Calcarea and Homoscleromorpha, although the latter is known to have cilia throughout the canals, and therefore presumably also up to the osculum lip.

**Figure 2:**
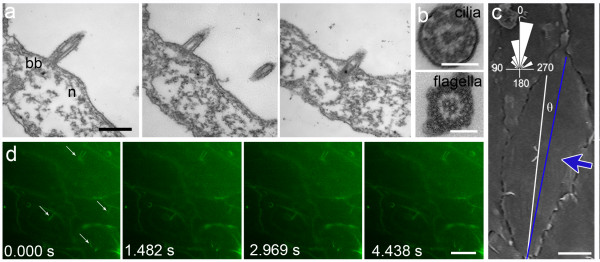
**Cilia are non-motile and are oriented perpendicular to the direction of water flow in the osculum. a**. Serial longitudinal sections (86 nm apart) show each cilium arises just above the cell nucleus (n) from simple basal bodies (bb); no links between the bases of the ciliary pair were found. **b**. In cross-section the cilium lacks a central microtubule pair in contrast to the cross section of a flagellum from a choanocyte chamber. **c**. Cilia pairs are aligned parallel to the long axis of the cells in the osculum, and both the cilia pairs and the cells’ long axes lie perpendicular to the direction of water flow (shown by the blue arrow) at 345.12 ± 4.72° (mean ± SE) (rose diagram: H_A_:0°; V = 0.841; p < 0.001; n = 49). **d**. Still images from high-frequency time-lapse imaging of live cilia (arrows) labeled with FM1-43 (see Additional file 2: Movie S1). Scale bars: **a**, 500 nm **b**, 100 nm **c**, 10 μm **d**, 20 μm.

Serial sections through the base of the cilium in *E. muelleri* show basal bodies are simple, with no structures linking pairs of cilia in a cell (Figure 2a). In contrast to the flagella of choanocyte chambers, which have a central pair of microtubules, in cross section the oscula cilia have a 9 + 0 axonemal skeleton (Figure 2b), which is characteristic of sensory cilia in other organisms [3]. Both fluorescence and scanning electron microscopy show pairs of cilia in *E. muelleri* are oriented perpendicular to the water flow (Figure 2c), which may be important for sensing changes in flow. In live animals the cilia label with the vital dye FM 1–43, and high frequency time-lapse microscopy showed that they are non-motile and only vibrate in the flow that passes out of the osculum (Figure 2d, and Additional file 2: Movie S1).

### Cationic channel blockers inhibit sponge behaviour

In the last decade it has been recognized that most cells in the vertebrate body, and many in invertebrates, possess specialized sensory structures called ‘primary’ cilia, which function as sensory organelles as in kidney epithelial cells, chondrocytes, odontoblasts, embryonic endocardial cells, and ‘Kupffer’s vesicle’ [22]. Primary cilia, although similar to motile cilia in their basic structure, lack the radial spokes and dynein arms that enable motility. Instead they possess stretch-activated cationic channels that are part of the transient receptor potential (TRP) channel superfamily [23] including polycystin-1 (PC1) and polycystin-2 (PC2) [23] or their homologs, which allow them to function as sensory organelles [3,22-24]. Remarkably, TRP channels are responsible for almost all forms of sensation experienced by eukaryotic cells, including movement, taste, smell, temperature, vision and osmolarity.

**Figure 3:**
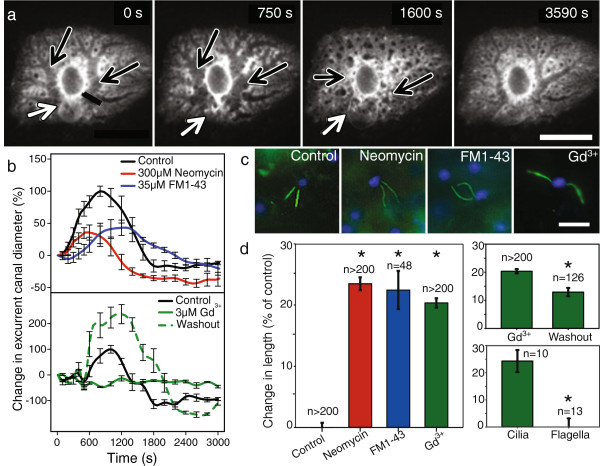
**Cationic channel blockers reduce the ‘sneeze’ response. a**. The sponge ‘sneeze’ behaviour involves contraction of the osculum (white arrows), inflation, then contraction of canals (black arrows) and recovery (bar shows canal diameter). **b**. Neomycin sulfate (red) and FM1-43 (blue) reduce the peak amplitude of the behaviour in *E. muelleri* (n = 8; p < 0.001). Gd^3+^ (solid green) eliminated all response (n = 3; p = 0.015), but after recovery for 24 h the sponge response was even greater than before (dotted green). **c**, **d** All three compounds caused lengthening of cilia relative to controls (left), but had no effect on choanocyte flagella (bottom right) in *E. muelleri* (*significance at p < <0.001; error bars show ± SE). Scale bars: **a**, 1,000 μm **c**, 10 μm.

The function of TRP channel sensation is difficult to assess directly, and is therefore usually done by behavioral assay; for example inhibition of an avoidance reaction by the unicellular alga *Chlamydomonas* using TRP channel blockers has shown that TRP11 is involved in mechanosensation [2]. In multicellular organisms it is difficult to study the function of primary cilia in living tissues, except in cell culture. In contrast, freshwater sponges are small and transparent, and cilia can be viewed live. Furthermore, both of the freshwater sponges *E. muelleri* and *S. lacustris* can be triggered to inflate and then contract their whole body (a behaviour termed a ‘sneeze’ [14,15]) in response to mechanical or chemical stimuli (Figure 3a). Because the osculum is the final channel through which water exits the sponge, we hypothesized that the cilia have a sensory role in controlling the canal diameter to optimize normal flow through the sponge filter, and in particular during the sneeze behaviour.

Three commonly used chemicals–the antibiotic neomycin sulfate, styryl dye FM1-43, and cationic channel blocker Gadolinium (Gd^3+^)–have been shown to inhibit sensory ability of primary cilia in other organisms [25,26]. These drugs are all thought to block TRPP2 (PC2) channels on the ciliary membrane. In sponges natural stimuli (sediment, vigorous mechanical agitation) as well as bath treatments of 75–90 μM L-glutamate trigger the inflation and contraction of the excurrent canals [14,15]. Treatment of sponges with neomycin sulfate (300 μM) and FM 1–43 (35 μM) reduced the maximum amplitude of the inflation response by 60% (Figure 3b) in both cases, and treatment with Gd^3+^ (5 μM) eliminated the response; the effects were reversible (Figure 3b). After recovery, the Gd^3+^-treated sponges showed an enhanced response to L-Glu (Figure 3b). This knock-down and knockout of the sponge behaviour by drugs that are known to affect channels on ciliary membranes implicates the cilia in sensing stimuli and transducing them into behaviour. Further support for this idea comes from the direct effect the drugs had on ciliary length.

Lengthening of primary cilia in other organisms has been proposed to increase their sensitivity [27,28]. Ciliary (and flagellar) length is determined by a dynamic process of intraflagellar transport (IFT) which continuously brings molecules, including tubulin, up and down the cilium [29]. Chemical or mechanical stimuli that interfere with Ca^2+^ influx have been shown to alter IFT, thereby changing cilium length [27,28]. In *E. muelleri* cilia length increased 1.2-fold after only one hour of treatment in all three drugs (Figure 3c, d), and Gd^3+^ treated sponges recovered partially after a one-hour washout. These data suggest that the drugs interfere with IFT in the oscula cilia. Unlike cilia, the flagella in choanocyte chambers of *E. muelleri* did not change length (Figure 3d), implying that the effects of the drugs reported here are only on ciliated cells.

**Figure 4:**
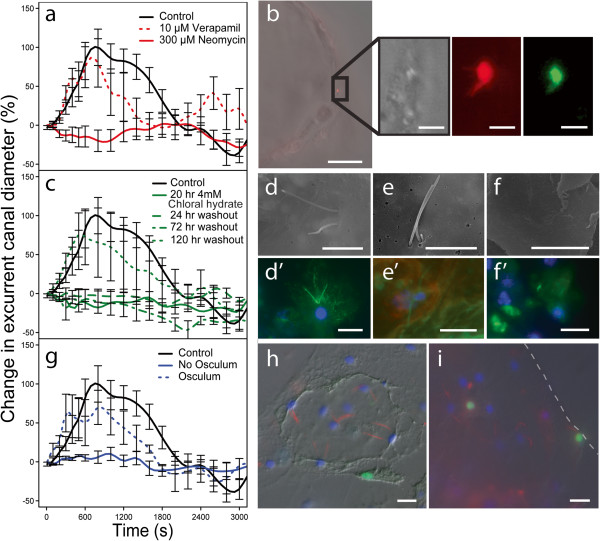
**Cilia are specifically involved in the sponge behaviour. a**. In contrast to Neomycin sulfate (solid red) which eliminates the ‘sneeze’ response (n = 3, p = 0.035), the calcium channel blocker Verapamil (dotted red) does not affect amplitude of the sneeze behaviour in *S. lacustris* (n = 5, p = 0.573). **b**. Texas-Red Neomycin sulfate conjugate (red) and YO-PRO1 (green) selectively label cells in the osculum. **c**. A 20 hr treatment in chloral hydrate eliminates the sneeze behaviour in *S. lacustris* (solid green; n = 5, p = 0.004), which does not return until more than 3 days after recovery (dotted green; n = 5, 24 hr washout p = 0.003, 72 hr washout p = 0.018, 120 hr washout p = 0.864)). **d-f(SEM) d’-f’(fluorescence)**. Cilia are removed by chloral hydrate treatment; *S. lacustris* 0 hr (**d**, **d’**), 20 hr (**e**, **e’**), and 70 hr (**f**, **f’**) treatment in chloral hydrate. **g**. The sneeze behaviour in *S. lacustris* cannot be triggered when the osculum is removed (solid blue; n = 3, p = 0.010) until it has fully regrown (dotted blue; n = 3, p = 0.275). **h**. Ciliated cells on the surface of *E. muelleri* 8 hr post osculum removal and (**i**) in the newly formed osculum 24 hr post osculum removal. Ciliated cells do not become labeled with EdU until after the osculum has regrown suggesting they arise by migration of newly formed mesohyl cells which differentiate into ciliated pinacocytes. Cilia are labeled with acetylated α-tubulin (red), nuclei with Hoechst (blue), and newly synthesized DNA with EdU (green). Scale bars: **b**, 50 μm inset 10 μm **d**, **e**, 5 μm **d’**, **e’**, **f**, **f’**, **h**, **i** 10 μm.

Although pharmacology is almost universally used to study the sensory roles of cilia and flagella in other organisms [2,25-27,30], neomycin sulfate, FM 1–43, and Gadolinium can also affect other calcium transport processes in tissues including smooth muscle contractility. We therefore tested whether another calcium channel blocker could equally affect the sponges. In contrast to neomycin sulfate which eliminates all response in the sponge, the L-type calcium channel blocker Verapamil (10 μM) had no effect on the amplitude of the sneeze reflex (Figure 4a). This finding is consistent with experiments on vertebrate primary cilia [25,26]. We found that longer incubation in Neomycin sulfate (2 hr in *S. lacustris* compared to 10 min for *E. muelleri*) repressed the sneeze reflex for longer. FM 1–43 is fluorescent and was clearly localized primarily to the cilia (Additional file 2: Movie S1), but to determine where neomycin sulfate localized we incubated sponges in Texas Red-conjugates of neomycin sulfate. Cells in the sponge osculum labeled within 2 minutes of incubation in the dye, and the same cells co-labelled with YO-PRO1, which selectively labels hair cells in the lateral line of zebrafish (*Danio rerio*) [31,32] (Figure 4b). Taken together, the effect of these treatments suggests that stretch-activated, nonselective cation channels are involved in the sponge behavior.

While we cannot rule out the possibility that any of these drugs have other effects on the sponge in addition to working on the cilia, in our experience very few molecules cause the sponge to relax–most trigger contractions [15,16]. However, to confirm that the cilia in the osculum, and the osculum itself, are indeed required for the sponge sneeze reflex we used both chloral hydrate to deciliate the sponge and removed the osculum, and tested the responsiveness of the sponge in each instance. Chloral hydrate is known to remove cilia from cells, causing a loss of behaviour in both metazoans [24] and unicellular eukaryotes [2,4] after 20 hr exposure. It is thought to act by weakening the attachment of the cilium to the basal body, with full loss of cilia occurring after 68 hr in kidney epithelial cells [24]. We found that 20 hr exposure to 4 mM chloral hydrate eliminated the sneeze reflex and it took 120 hr for recovery of sensitivity (Figure 4c-e). As in kidney cells [24], it took 70 hr to remove all cilia from the epithelium of the osculum (Figure 4f).

We have found that when removed, a new osculum forms again after 8 hours. De-osculated sponges could not be triggered to sneeze (Figure 4g), and although the sponge continued to filter water at all times during repair of the osculum, it was only after the osculum had fully formed that the sneeze response returned. Together these results suggest that both the osculum and the cilia lining it are necessary for the sneeze reflex. To determine when ciliated cells first appear on newly formed oscula, we labeled sponges from which the osculum had been removed with the cell proliferation marker EdU and detected incorporation of uridine into new cells using Click-iT (Molecular Probes, Invitrogen). At 8 hr after the osculum was removed, cilia were found on cells in a few discrete places on the surface of the sponge (Figure 4h). Pinacocytes in the sponge surface are not usually ciliated, therefore we interpreted the differentiation of cilia on pinacocytes as an early marker of the location of a new osculum. Furthermore, although mesohyl cells were labeled within 6 hrs of incubation in EdU, cells of the new osculum were not labeled with EdU, and it was only 24 hr after the new osculum was formed that a few new ciliated cells labeled (Figure 4i). Although we were unable to trace the migration of cells in live animals, we interpret these data to suggest that cilia differentiate on cells in the surface of the sponge, thereby identifying the region as a potential osculum; then as the osculum grows to full height using cells already present in the sponge, new ciliated epithelial cells differentiate from newly formed mesohyl cells.

### Sponges possess a repertoire of transient receptor potential channels

**Figure 5:**
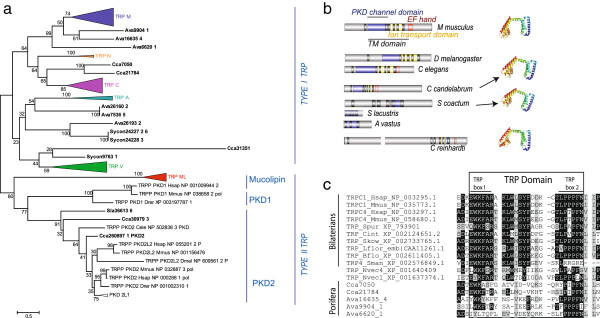
**Phylogenetic analysis of sponge TRP genes. a**. Evolutionary relationships of sponge TRP Type I and II genes, values in the nodes indicate Boostrap Support and Posterior Probabilities (see Methods); sponge sequences are in bold. **b**. Domain diagrams showing the PKD channel domain, transmembrane domain (TM), EF hand domain, and ion transport domains for the *pkd2* genes from mouse, *Mus musculus*; Cca, *Corticium candelabrum* (Homoscleromorpha); Cel, *Caenorhabditis elegans;* Sla, *Spongilla lacustris* (Demospongiae); Sco; *Sycon coactum* (Calcarea); Ava, *Aphrocallistes vastus* (Hexactinellida); Cre, *Chlamydomonas reinhardtii*, and 3D models of the proteins from mouse, *Corticium, Sycon,* and *Chlamydomonas*. **c**. Alignment of bilaterian, cnidarian and sponge TRP sequences showing the TRP domain and TRPbox (Hsap, *Homo sapiens;* Mmus, *Mus musculus;* Spur, *Strongylocentrotus purpuratus;* Cint, *Ciona intestinalis,* Sko, *Saccoglossus kowaleskii,* Lforb, *Loligo forbesi*, Bflo, *Branchiostoma floridae,* Sman, *Schistosoma mansoni,* Nvec, *Nematostella vectensis*). For the full tree and alignment see Additional file 1: Figures S2-S4.

Considering the conserved role of TRP channels, and in particular PKD in sensory behaviour across eukaryotes [2], we searched the transcriptomes of 8 sponge species for homologs of both *pkd1* and *pkd2* and other TRP channels. A 700aa homolog of *pkd2* (Type II TRP) was identified in *Corticium candelabrum* (Homoscleromorpha) and a 178aa sequence of a *pkd2* (Type II TRP) gene was found in the freshwater *Spongilla lacustris* (Demospongiae) (Figure 5a, Additional file 1: Figures S2, S3). We found a 978aa sequence of a Type II TRP (ML) in *Sycon coactum* (Calcarea), and several sequences with similarity to various Type I TRP channels were found in all 4 Porifera classes (Figure 5a-c, Additional file 1: Figure S5). These candidates were included in an alignment containing more than 100 representatives for all the TRP families across bilaterians (Figure 5a; Additional file 1: Figures S2-S3). The ability to retrieve protein sequences depends on the quality of the transcriptome and the divergence of sequences in transcriptomes. Negative results do not imply conclusive absence. Our phylogenetic analysis grouped sponge *pkd* sequences with Type II TRP and specifically *pkd2* channels genes from bilaterians with high support (91% bootstrap). Sponge *pkd* channel sequences showed similar domain architecture and proposed 3D protein folding to both mouse and *Chlamydomonas* sequences (Figure 5b), and other sponge sequences showed amino acids indicative of the TRP domain (Figure 5c; Additional file 1: Figure S5). Although the pharmacology of the sponge cilia is similar to that of cilia known to have *pkd2* channels, several TRP channels from *Chlamydomonas* have also been found to transduce mechanical signals so we cannot rule out the possibility that other TRP channels are involved in flow sensing in sponges.

## Conclusions

In the sponge, obstruction of the canals by particulates in the feeding current would cause changes in pressure across the system; the osculum is the single exit of the entire system and is expected to be sensitive to this change, so it is plausible that the cilia detect changes in water flow or pressure. The absence of motility of the cilia, and their specific localization to the inner lining of the sponge osculum strongly suggest a sensory role for the osculum; the pharmacology and ablation experiments also support the hypothesis that the cilia have a sensory function. The primary cilium, which extends out from the cell and has a high surface-area to volume ratio, is an ideal organelle for both sensing and transducing signals [3]. These cilia in the sponge osculum have all the characteristics of primary cilia.

While the role of cilia in sensing information may have evolved many times within eukaryotes, the sponge sensory system described here is certainly very similar to signalling via primary cilia in other metazoans [22]. The role of cilia in the sponge osculum suggests either a convergent role in sensing and transducing flow information into behaviour across all metazoa, or implies that primary cilia had an ancient evolutionary role in transducing sensory information, and in particular flow, in early multicellular animals. Given the unique position of Porifera as extant representatives of one of the first groups of multicellular animals [9], and in particular their lack of conventional nervous and coordination systems, the finding of such an organized array of sensory cells in sponges provides new insight into possible mechanisms of evolution of early sensory systems.

## Methods

### Collecting and culturing of sponges

Gemmules of the freshwater sponges *Ephydatia muelleri* and *Spongilla lacustris* were collected from Frederick Lake, BC and Rousseau Lake, BC, respectively, at a depth of 0–3 m and stored at 4°C in unfiltered lake water, aerated monthly, until use. The spicule skeleton was removed from the gemmules by gently rubbing between two pieces of corduroy, and the gemmules were then sorted, sterilized (using 1% H_2_O_2_ for 5 min), and rinsed in cold distilled water. Single gemmules placed onto ethanol sterilized glass coverslips in Petri dishes containing M-medium [33], hatched in 2–3 days; culture medium was changed every 2d post hatching (dph). Only 5-10 dph sponges with fully developed aquiferous canal systems were used in experiments.

### Fixation for fluorescence microscopy

Sponges on coverslips were fixed in 3.7% paraformaldehyde and 0.3% glutaraldehyde in 100 mM phosphate-buffered saline (PBS) for 12–24 h at 4°C. Preparations were rinsed three times in cold PBS, permeabilized with PBS + 0.1% Triton X-100 (PBTX) for two minutes and rinsed in PBS. Either whole juvenile sponges or individual oscula (pulled off of the sponge by pinching the base of the osculum with fine forceps) were labeled with mouse anti-acetylated alpha tubulin (Sigma-Aldrich, Oakville, ON) in 10% goat serum (GS) and PBS at 1:1000 at RT overnight. Preparations were rinsed in PBS and incubated in goat anti-mouse 488 (Molecular Probes, Burlington, ON) at 1:100 in 10% GS and PBS overnight. Nuclei were counterstained with Hoescht 33342 (Sigma-Aldrich) 1:100 in PBS for 10 min. Some preparations were stained for actin using Alexa 594 phalloidin (Molecular Probes) in BSA-PBS. Labelled oscula were sliced open using a microscalpel, mounted on a slide in 100% glycerol, and sealed with nail polish. Whole sponges on coverslips were inverted onto a slide in 100% glycerol, sealed with nail polish and viewed with a Zeiss Axioskop2 Plus. Confocal images were taken using a Zeiss LSM 710, and surface rendering was done using Imaris v7.2 (Bitplane, Zurich, Switzerland).

### Fixation for scanning and transmission electron microscopy (SEM, TEM)

Hatched sponges were fixed and prepared for electron microscopy as described previously [14]. For SEM oscula were removed from the sponges, dehydrated to 100% ethanol and critical point dried. Dried oscula were mounted on aluminum stubs using adhesive tabs and gold-coated prior to viewing using a scanning electron microscope (JEOL 6301 F field emission or a Zeiss EVO MA 15). For TEM oscula were dehydrated through 100% ethanol and embedded in epoxy (TAAB 812). Ultrathin sections (60 nm) were stained with uranyl acetate and lead citrate and viewed in a Hitatchi H-7000 or Phillips Morgagni (FEI) TEM and images captured with an AMT or Gatan digital camera respectively.

### Orientation analysis

To assess orientation of each cilia pair with respect to the direction of water flow along the osculum, a line was drawn between the base of the two cilia and the angle between that line and a line defining the long axis of the cell was calculated using ImageJ (v1.43r; NIH, Bethesda, MD). Circular statistics calculated with Oriana v. 3.13 (KCS, Wales, UK) gave the mean angle of the orientation of cilia pairs and a V-test was performed to determine difference from the long axis of the cell.

### Assessment of the possible sensory role

Stock solutions of 10 mM neomycin sulfate (Fisher BioReagents, New Jersey), 1 g/L (178.5 M) FM 1-43FX (Fixable analog; Molecular probes, Invitrogen), 10 mM of GdCl_3_ (Sigma-Aldrich), 20 mM Verapamil (Sigma-Aldrich), and 1 M Chloral hydrate (Sigma-Aldrich) were kept covered at 4°C and used at 300 μM, 35 μM, 5 μM, 10 μM, and 4 mM respectively. Neomycin sulfate and FM1-43FX were added to the Petri dish and the sponge was stimulated using agitation (vigorous shaking of the Petri dish for 30 s) 10 min later for *E. muelleri* or 2 hr later for *S. lacustris*. Gd^3+^ and Verapamil were added to the Petri dish 2 hr prior to stimulation with 75–90 μM L-Glutamate. Treatment in chloral hydrate was for 20 hr prior to stimulation with 75–90 μM L-Glutamate; during washout the M-medium was changed every 2d and the sponge was then stimulated with 75–90 μM L-Glutamate. Oscula were removed by pinching the base of the osculum with fine-tipped forceps, and the sponge was stimulated at 2 hr and then again at 24 hr using agitation. Care was taken to add each treatment to the side of the Petri dish away from the sponge, and the solution was mixed by pipetting gently 5–6 times. Images were captured every 10 s for 50 min, or until the sponge had completed an inflation/contraction cycle. Still images were captured in Northern Eclipse v.7 (Empix Imaging Inc., Mississauga, ON, Canada). Changes in canal diameter were measured every tenth image for the first 60 images, and then every 20^th^ image, using ImageJ (v1.43r; NIH). Due to high variation in changes in canal diameters within a single sponge, three canals in each sponge were measured for the neomycin and FM test and a nested ANOVA was run in R (v.2.4.1). The variance between canals within a single individual did not account for any of the variance in the dataset, therefore only one canal was measured per sponge in the remaining experiments and tested via a one-way ANOVA in R (v.2.4.1). All data were tested for normality using a Shapiro-Wilks test, with Gd^3+^ data log(x) transformed and chloral hydrate data square root transformed.

Cilia length of sponges treated with neomycin sulfate, FM1-43FX and Gd^3+^, for one hour each, were measured from fluorescence images with ImageJ (v1.43r). Untreated sponges were used as controls. Reversibility of Gd^3+^ treatment was demonstrated by washing out the blocker for 1 hr in culture medium prior to fixation. Cilia and flagella length of Gd^3+^-treated sponges were measured from SEM images. The measurements were log(x) transformed and analyzed using a nested ANOVA in R (v.2.4.1).

Texas-Red conjugated neomycin (TR-Neo) was made by shaking neomycin sulfate (50 mg/ml in K_2_CO_3_) and Texas Red (Molecular Probes, Invitrogen; 2 mg/ml in dimethylformamide) overnight [34], and added to M-medium to a final concentration of 300 μM neomycin sulfate. *S. lacustris* was treated for 2 min in TR-Neo followed by three rinses in M-medium, 5 min in 1 μM YO-PRO1 (Invitrogen) [31,32], and three more rinses in M-medium prior to viewing live using a 40X Zeiss water immersion objective.

Both whole *S. lacustris* and oscula removed from the sponge were treated in 4 mM chloral hydrate for 20 hr or 70 hr (medium changed daily to maintain concentration), and fixed for fluorescence microscopy with anti-tubulin and for SEM as described above. Click-iT EdU imaging kit (Invitrogen) was used to label newly synthesized cells post osculum removal. *E. muelleri* was incubated in 50 μM EdU in M-medium for 8 hr or 24 hr after the osculum was removed, fixed for fluorescence, and labeled using the Click copper-catalyzed covalent reaction. Sponges were labelled with acetylated alpha tubulin and Hoechst as described above.

### BioInformatics

The transcriptomes of 8 sponge species (*Ephydatia muelleri, Spongilla lacustris, Petrosia ficiformis, Chondrilla nucula, Ircinia fasciculata, Corticium candelabrum, Sycon coactum, Aphrocallistes vastus)* were sequenced using Illumina and assembled *de novo* in either Trinity or CLCGenomics Workbench 5.1 [35]. TRP sequences in these transcriptomes and also in the *Amphimedon queenslandica* genome [36] were detected using HMMer (Janelia.org) using HMM profiles formed with *pkd1* and *pkd2* sequences collected from NCBI or by blasting NCBI sequences against the transcriptome datasets using the tblastn suite in CLC Genomics Workbench. Sequence identity and domain conservation was confirmed by BLAST and NCBI’s conserved domain search as well as EMBL’s InterPro Scan; domain illustrations were conceived using DOG2.0 and 3D models projected using Phyre2 [37]. TRP channel and PKD channel sequences from bilaterians were downloaded from SwissProt following the (vertebrates) taxon sampling for TRP and PKD domains in Pfam [38]; SwissProts accession numbers are indicated in the sequence labels. *Chlamydomonas reinhardti* PKD2 ABR14113.1 was downloaded from NCBI. For phylogenetic analysis sequences were aligned in MAFFT [39] using the E-INSI algorithm, and positions shared by 85% of the taxa were selected using MEGA5.1 [40] for further phylogenetic analyses. Evolutionary relationships were inferred by ML using the evolutionary model LG [41] + GAMMA + Invariants as implemented in RAxML [42]. The statistical support of the branches was obtained by generating 1000 bootstrap pseudoreplicates. (The full alignment of 344aa and tree are shown in Additional file 1: Figures S2, S3.) The same dataset was analyzed under the Bayesian Inference framework/Phylobayes-MPI [43] under the CAT-GTR [44] model (Additional file 1: Figure S4). The tree search was conducted during 7,500 cycles, and a burning of 1000 trees (sub-sampling every 10 trees) was used to discard the trees before the search reached the likelihood optima.

### Availability of supporting data

Full alignments and trees are provided in supplemental data files. Sponge sequences described here have been deposited at DDBJ/EMBL/GenBank under the BioProjects PRJNA162903, PRJNA225591, PRJNA162899, PRJNA225584.

## Competing interests

The authors declare that they have no competing interests.

## Authors’ contributions

DAL and SPL conceived the experiments; SPL collected the sponges; DAL performed the experiments and carried out the statistical analysis; DAL and SPL carried out the electron microscopy; NF, AR, SPL and JP performed the molecular analysis; DAL and SPL wrote the paper. All authors read and acknowledged the final version of the manuscript.

## Supplementary Material

Additional file 1: Figure S1 Cilia in the oscula of various demosponges a. *Ephydatia muelleri,*b, c. *Spongilla lacustris,* d. *Neopetrosia vanilla,* e. *Haliclona mollis,* f. *Haliclona* sp,*.* g. *Neopetrosia problematica,* h. *Aphrocallistes vastus.* Scale bars 1 μm. **Figure S2**: Uncompressed tree showing the evolutionary relationships of sponge TRP Type I and II genes. Values at nodes indicate Bootstrap support. **Figure S3**: Full alignment of TRP sequences for the uncompressed tree from Figure 5a. **Figure S4**: Phylobayes alignment of data in 5c. **Figure S5**: Full alignment of sequences in Figure 5c and list of Sponge TRP Fastas. Click here for file

Additional file 2: Movie S1 Cilia in the osculum of a live sponge, *Ephydatia muelleri,* labeled using FM1-43. High-frequency time-lapse microscopy (images taken at 50 millisecond intervals with exposure of 50 milliseconds) indicates that the cilia are non-motile and only vibrate in the flow that passes out the osculum. Click here for file
