# Supplementary material for: Evolutionary origins of sensation in metazoans: functional evidence for a new sensory organ in sponges
Source: BMC Evol Biol. 2014 Jan 13;14:3. doi: 10.1186/1471-2148-14-3 (PMC3890488; doi:10.1186/1471-2148-14-3)
Supplement: Additional file 1: Figure S1 — Cilia in the oscula of various demosponges a. Ephydatia muelleri,b, c. Spongilla lacustris, d. Neopetrosia vanilla, e. Haliclona mollis, f. Haliclona sp,. g. Neopetrosia problematica, h. Aphrocallistes vastus. Scale bars 1 μm. Figure S2: Uncompressed tree showing the evolutionary relationships of sponge TRP Type I and II genes. Values at nodes indicate Bootstrap support. Figure S3: Full alignment of TRP sequences for the uncompressed tree from Figure 5a. Figure S4: Phylobayes alignment of data in 5c. Figure S5: Full alignment of sequences in Figure 5c and list of Sponge TRP Fastas. [file 1471-2148-14-3-S1.pdf]

SUPPLEMENTARY INFORMATION

Sensory cilia in sponges

Ludeman, DA[1], Farrar, N[1], Riesgo, A[2], Paps, J[3] and Leys, SP[1*]

[1]Department of Biological Sciences, University of Alberta, Edmonton, Alberta T6G 2E9, Canada.

[2]Department of Animal Biology, Universitat de Barcelona, Avinguda Diagonal 643, 08028, Barcelona, Spain

[3]Department of Zoology, University of Oxford, Oxford, OX1 3PS, United Kingdom.

*Author for correspondence

Address: CW 405 Biological Sciences Building, University of Alberta, Edmonton, Alberta T6G 2E9, Canada

Phone: (780) 492-6629

Fax: (780) 492-9234

Email: sleys@ualberta.ca

This .pdf file includes:

Supplementary Figure S1

Supplementary Figure Legends S2-S5

Supplementary Figures S2-S5

Supplementary Movie Legend S1

Supplementary Figure S1

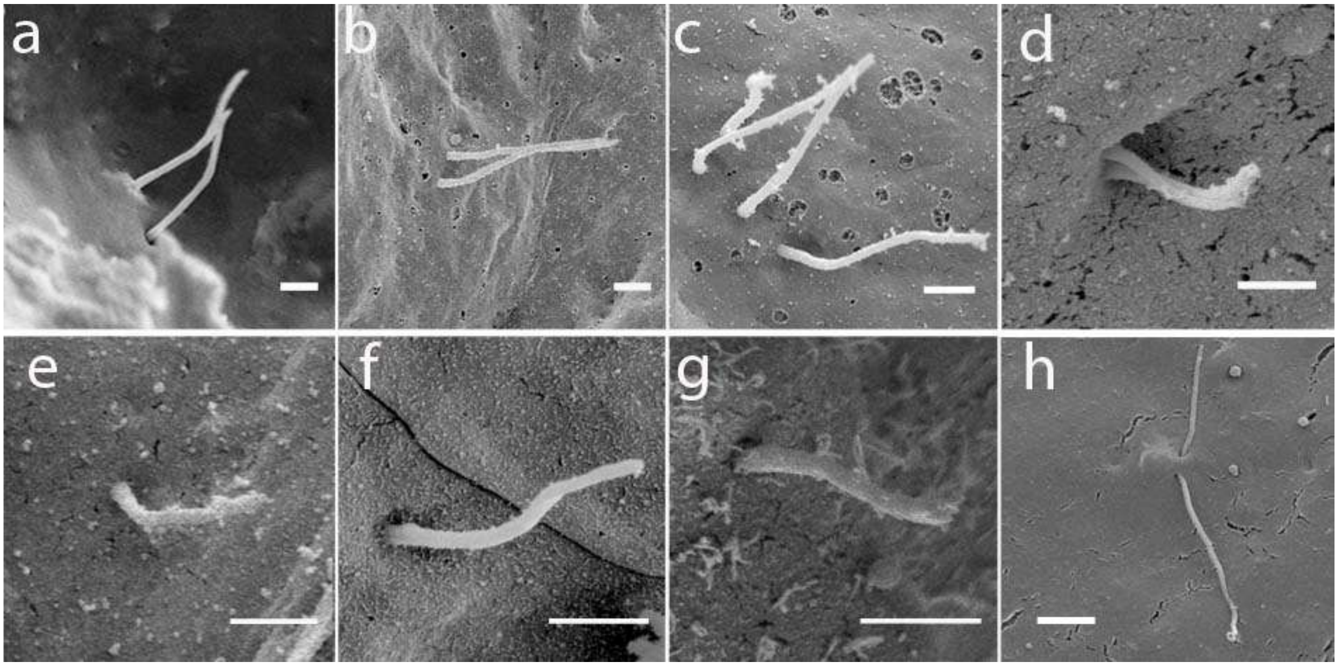

Supplementary Figure S1| Cilia in the oscula of various demosponges a. *Ephydatia muelleri,* **b, c.** *Spongilla lacustris,* **d.** *Neopetrosia vanilla,* **e.** *Haliclona mollis,* **f.** *Haliclona* sp,. **g.** *Neopetrosia problematica,* **h.** *Aphrocallistes vastus.* Scale bars 1 µm.

Supplementary Figure S2| Uncompressed tree showing the evolutionary relationships of sponge TRP Type I and II genes. Values at nodes indicate Bootstrap support.

Supplementary Figure S3| Full alignment of TRP sequences for uncompressed tree in from Fig 5a

Supplementary Figure S4| Phylobayes alignment of data in 5c

Supplementary Figure S5 Full alignment of sequences in Figure 5c and list of Sponge TRP Fastas

Supplementary Movie Legend

Supplementary Movie S1| Cilia in the osculum of a live sponge, *Ephydatia muelleri,* labeled using FM1-43. High-frequency time-lapse microscopy (images taken at 50 millisecond intervals with exposure of 50 milliseconds) indicates that the cilia are non-motile and only vibrate in the flow that passes out the osculum.

Supplementary Figure S2
Uncompressed form of tree
shown in figure 5a

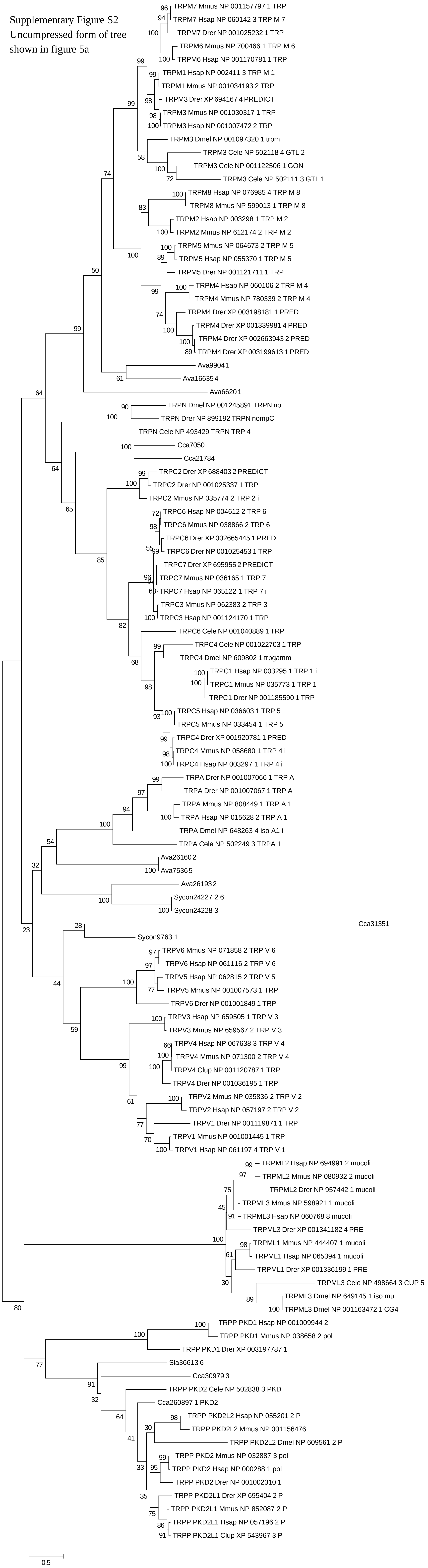

Supplementary figure S3: Definitive alignment used for tree shown in figure 5a

```
TRPC1_Hsap   _NP_003295 _1_TRP_1_i LFLLACDKGD YYMVKKILEN LDILQLLLDY G--------- ---------- ---------IL
TRPC1_Mmus   _NP_035773 _1_TRP_1LF LLACDKGDYY MVKKILESLD ILQLLLDYGS ADALLVAIDS EVVGAVDILL NHRKRSILAA
TRPC1_Drer   _NP_001185 590_1_TRP_ LFLLACEKGD YYMVKKLLEN LDILQLLLDH GATDALLVAI DSEVVGAVDI LLNHRRRSIL
TRPC2_Mmus   _NP_035774 _2_TRP_2_i TLLRAIQEGQ LGLVQQLLGH EVITDVLLAK FIHEALLVAV DTNQPAVVRR LLARLERETL
TRPC2_Drer   _NP_001025 337_1_TRP_ ELLGAIREGN LNLVSSLLGN EDTMASLLQK FIHEALLVAV DTNQPRVVKR LLDRLDQETL
TRPC2_Drer   _XP_688403 _2_PREDICT ELILAIQEGN MAWVDSLLGS EDIMTSLLLK FIHEALLVAV DTNQPRFVKH LLDRLDLETL
TRPC3_Hsap   _NP_001124 170_1_TRP_ RFLDAAEYGN IPVVRKMLEH LEVTELLLKK EIGDALLLAI SKGYVRIVEA ILNHPGFAIL
TRPC3_Mmus   _NP_062383 _2_TRP_3RF LDAAEYGNIP VVRKMLEHLE VTELLLKKEI GDALLLAISK GYVRIVEAIL GHPGFAILAA
TRPC4_Hsap   _NP_003297 _1_TRP_4_i AYLNAVEKGD YASVKKSLEN LELIELLLSF NVGDALLHAI RKEVVGAVEL LLNHKKPSIL
TRPC4_Mmus   _NP_058680 _1_TRP_4_i AYLNAVEKGD YASVKKSLEN LELIELLLSF NVGDALLHAI RKEVVGAVEL LLNHKKPSIL
TRPC4_Drer   _XP_001920 781_1_PRED SYLSAVEKGD YASVKLALEN LEIIELLLSF NVGDALLHAI RKEVVGAVEL LLNHKKPSIL
TRPC4_Dmel   _NP_609802 _1_trpgamm KFLLAVERGD MAGTRRMLEN LEMVELLINY NTKDALLHSI SEEFVEAVEV LLDHENVTIL
TRPC4_Cele   _NP_001022 703_1_TRP_ QFLLSCERGD IGSVRKLLEN IEMIELLLDH NTGDAILYAI GEENVEAVEI IVEHLKMDVL
TRPC5_Hsap   _NP_036603 _1_TRP_5AF LNAVEKGDYA TVKQALENLE IMELLLNHSV GDALLYAIRK EVVGAVELLL SYRRPSMLAA
TRPC5_Mmus   _NP_033454 _1_TRP_5AF LSAVEKGDYA TVKQALENLE IMELLLNHSV GDALLYAIRK EVVGAVELLL SYRKPSMLAA
TRPC6_Hsap   _NP_004612 _2_TRP_6RF LDAAEYGNIP VVRKMLEHLE ITELLLKKEV GDALLLAISK GYVRIVEAIL SHPAFAILAA
TRPC6_Mmus   _NP_038866 _2_TRP_6RF LDAAEYGNIP VVRKMLEHLE ITELLLKKEV GDALLLAISK GYVRIVEAIL NHPAFAILAA
TRPC6_Drer   _NP_001025 453_1_TRP_ RFLDAAEYGN IPVVRRMLEH LEVTELLLKK DIGDALLLAI SKGYTRIVEA ILSHRAFAIL
TRPC6_Drer   _XP_002665 445_1_PRED RFLEAAEYGN IPVIRRMLEH LEVTELLLKK EIGDALLLAI SKGYIRIVEA ILSHQAFAIL
TRPC6_Cele   _NP_001040 889_1_TRP_ RFLEAAELGN KPTLQECLEN MEVVELLLQQ PIGNALLCAI REGVYRLVEV LVNHPNITIL
TRPC7_Hsap   _NP_065122 _1_TRP_7_i RFLDSAEYGN IPVVRKMLEH LEVTELLLKK EVGDALLLAI SKGYVRIVEA ILNHPAFAIL
TRPC7_Mmus   _NP_036165 _1_TRP_7RF LDSAEYGNIP VVRKMLEHLE VTELLLKKEV GDALLLAISK GYVRIVEAIL SHPAFAILAA
TRPC7_Drer   _XP_695955 _2_PREDICT RFLDAAEYGN IPSVRKMLEH LEVTELLLKK EVGDALLLAI SKGYVRIVEA ILAHPAFEIL
TRPN_Dmel_   NP_0012458 91_TRPN_no PMHLAAENGH AHVIEILAGH MELVNNLLAA RGRSALHLAA ERGYLHVCDA LLTNKFINHL
TRPN_Cele_   NP_493429_ TRPN_TRP_4 PVHVAAERGD TSMVESLIGH SGVANILLKA RGRTALHLAA FNGHLSLVHL LLQHKFVNHL
TRPN_Drer_   NP_899192_ TRPN_nompC PLHIAAERGH TNVVEILTGH TEVVKILLQA RGKAAIHLAA QRGHQDIVDV LLSQKFVNHL
TRPM1_Hsap   _NP_002411 _3_TRP_M_1 PLVGLVVEGG PNVVSIVLVD IARSQIFVFH WLEQAMLDAL VLDRVDFVKL LIENGNMQRL
TRPM1_Mmus   _NP_001034 193_2_TRP_ PVVGLVVEGG PNVVSIVLVD IARSQIFVFH WLEQAMLDAL VLDRVDFVKL LIENGNMQRL
TRPM2_Hsap   _NP_003298 _1_TRP_M_2 PIVCVVLEGG PGTLHTIDVD IARSEIFMDQ WLHPTMTAAL ISNKPEFVKL FLENGQLKTL
TRPM2_Mmus   _NP_612174 _2_TRP_M_2 PIVCVVLEGG PGTLHTIYVD IARSEIFTDQ WLHPMMTAAL ISNKPEFVRL FLENGRLKTL
TRPM3_Hsap   _NP_001007 472_2_TRP_ PVVALIVEGG PNVISIVLVD IARSQIFIYQ WLEQAMLDAL VLDRVDFVKL LIENGSMHRL
TRPM3_Mmus   _NP_001030 317_1_TRP_ PVVALIVEGG PNVISIVLVD IARSQIFIYQ WLEQAMLDAL VLDRVDFVKL LIENGSMHRL
TRPM3_Drer   _XP_694167 _4_PREDICT PVVALIVEGG PNVISIVLVD IARSQIFIYQ WLEQSMLDAL VLDRVDFVKL LIENGSMHRL
TRPM3_Dmel   _NP_001097 320_1_trpm PVVCLVIEGG TNTIRAVLVD IARSEIFVYE WLDEAMMQAL EHDRIDFVKL LLENGSMKRL
TRPM3_Cele   _NP_001122 506_1_GON_ PVVCVVLEGG SCTIRSVLVD IARSDVFAME WLHNAMMEAL IHDRVDFVRL LLEQGNMQRL
TRPM3_Cele   _NP_502111 _3_GTL_1PL VCVVLEGGAF TIKMVHADIA RTEIFANEWL HNAMIEALSN DRIDFVHLLL ENGSMQRLEH
TRPM3_Cele   _NP_502118 _4_GTL_2PV VCTLLEGGIS SINAIHVDLA KSCLFSNKWL EKAMNDALYW DRVDFVECLL ENGSMKRLEN
TRPM4_Hsap   _NP_060106 _2_TRP_M_4 PVLLLLIDGD EKMLTRIEVD IAQSELFRGQ WLEASLMDAL LNDRPEFVRL LISHGSLGRL
TRPM4_Mmus   _NP_780339 _2_TRP_M_4 PVLLLLIDGD EKMLKRIEVD IAQSELFRGQ WLEASLMDAL LNDRPEFVRL LISHGSLGRL
TRPM4_Drer   _XP_003199 613_1_PRED PVLNMLIAGD TSMLEFLEVD IAKSELFNGD WLVDSMTDAL ENNKPQFVRL LIDNGNILRL
TRPM4_Drer   _XP_001339 981_4_PRED PVLNMLIAGD TSMLEFLEVD IAKSELFNGD WLKDSMTDAL ENNKPQFVRL LIDNGNISRL
TRPM4_Drer   _XP_002663 943_2_PRED PVLNMLIAGD TSMLEFLEVD IAKSELFNGD WLVDSMTDAL ENNKPQFVRL LIDSGNILRL
TRPM4_Drer   _XP_003198 181_1_PRED PVLCVLIAGD ATMLERVDVD IAKTELFNGH WLEESMTDAL VNDKPDFVRL FVESGNILRL
TRPM5_Hsap   _NP_055370 _1_TRP_M_5 PVLCLLVNGD PNTLERISVD IAKSEIFNGE WLEEVMVDAL VSNKPEFVRL FVDNGDVARL
TRPM5_Mmus   _NP_064673 _2_TRP_M_5 PVLCLLVNGD PNTLERISVD IAKSEIFNGE WLEEVMTDAL VSNKPDFVRL FVDSGDMARL
TRPM5_Drer   _NP_001121 711_1_TRP_ PVLCLLVHGE PRILQKMYVD IAKSEIFSGQ WLEEVMMEAL VNDKPDFVRL FVDNGNIKRL
TRPM6_Hsap   _NP_001170 781_1_TRP_ PVVGLVVEGG PNVILSVWVD IAKKHILIYH WLEQAMSDAL VMDRVDFVKL LIEYGNLHRL
TRPM6_Mmus   _NP_700466 _1_TRP_M_6 PVVGLVMEGG PNVILWVWMD IAKKHILTYH WLEQAMLDAL VMDRVDFVKL LIENGNLHRL
TRPM7_Hsap   _NP_060142 _3_TRP_M_7 PVVALIFEGG PNVILTVLVD IAKNHVFVYQ WLEQAMLDAL VMDRVAFVKL LIENGSMHRL
TRPM7_Mmus   _NP_001157 797_1_TRP_ PVVALIFEGG PNVILTVLVD IAKNHVFVYQ WLEQAMLDAL VMDRVSFVKL LIENGSMHRL
TRPM7_Drer   _NP_001025 232_1_TRP_ PVVALIFEGG PNVILTVLVD IAKDHVFVYQ LLEQAMLDAL VMDRVEFVKL LIENGSMHRL
TRPM8_Hsap   _NP_076985 _4_TRP_M_8 PIVCFAQGGG KETLKAINLD LANDEIFTNR WLQEVMFTAL IKDRPKFVRL FLENGLRVRL
TRPM8_Mmus   _NP_599013 _1_TRP_M_8 PIVCFAQGGG RETLKAINLD LASDEIFTNR WLQEVMFTAL IKDRPKFVRL FLENGLNLQVL
TRPA_Hsap_   NP_015628_ 2_TRP_A_1P LHIAVQGMNN EVMKVLLEHG YSRQLHINNN KATPLHLAVQ NGDLEMIKMC LDNGQIDILA
TRPA_Mmus_   NP_808449_ 1_TRP_A_1P LHIAVHGMYN EVIKVLTKNG YSRETHINNH KASPLHLAVQ SGDLDMIKMC LDNGHIDILA
TRPA_Drer_   NP_0010070 66_1_TRP_A PIHMAVSLGK NFVLEQLVEA GLSIDAHIND KCSSPLHLAV RGGNLDIIKL CIGYGKIDLL
TRPA_Drer_   NP_0010070 67_1_TRP_A PLHLAVTKQY NHLAEVLLEL GVSSTLHIND KKSTPLHLAV RGGNIEVIKL CILKGKVELL
TRPA_Dmel_   NP_648263_ 4_iso_A1_i PVHLATELNK VKSLRVMGQR GCTREEMISD SGNVPLHSAV HGGDIKAVEL CLKSGKISLL
TRPA_Cele_   NP_502249_ 3_TRPA_1PL HYAAMKSNFS ALHALIHDPV EAIKALNNNK KTPLRMAVEG NHPETLKKIL QMEKNSMMAV
TRPV1_Hsap   _NP_061197 _4_TRP_V_1 SIFEAVAQNN CQDLESLLQT DSLKELVNAT DGQTALHIAI ERRNMALVTL LVENGDVQSL
TRPV1_Mmus   _NP_001001 445_1_TRP_ SIFDAVAQSN CQELESLLKT DSLKQFVNAT DGQTALHIAI ERRNMALVTL LVENGDVQSL
TRPV1_Drer   _NP_001119 871_1_TRP_ RLFEAVSSGD VSKMQGLHKM GDLKNFINAT DGQTALHVAI ERRSMKFVQM LVKKGDVHSL
TRPV2_Hsap   _NP_057197 _2_TRP_V_2 RLFNAVSRGV PEDLAGLPDS GNPQPLVNAT DGHSALHIAI EKRSLQCVKL LVENGNVHSL
TRPV2_Mmus   _NP_035836 _2_TRP_V_2 RLFSVVSRGV PEELTGLLDS GNPQPLVNAT DGHSALHIAI EKRSLWCVKL LVENGNVHSL
TRPV3_Hsap   _NP_659505 _1_TRP_V_3 RIFAAVSEGC VEELVELLEN DILGRFINAT EGQTALNIAI ERRQGDIAAL LIAAGDVNAL
TRPV3_Mmus   _NP_659567 _2_TRP_V_3 RIFAAVSEGC VEELRELLEN DILDRFINAT EGQTALNIAI ERRQGDITAV LIAAGDVNAL
TRPV4_Hsap   _NP_067638 _3_TRP_V_4 ILFDIVSRGS TADLDGLLRT GNMREFINSR DGQTALHIAI ERRCKHYVEL LVAQGDVHSL
TRPV4_Clup   _NP_001120 787_1_TRP_ ILFDIVSRGS TTDLDGLLRT GNMREFINSR DGQTALHIAI ERRCKHYVEL LVAQGDVHSL
TRPV4_Mmus   _NP_071300 _2_TRP_V_4 ILFDIVSRGS TADLDGLLRT GNMREFINSR DGQTSLHIAI ERRCKHYVEL LVAQGDVHSL
TRPV4_Drer   _NP_001036 195_1_TRP_ MLFEAVSRAD PRALDGLLQT GNLREFINTR DGQMALHIAI ERRCKQYVEL LVEKGDVHSL
TRPV5_Hsap   _NP_062815 _2_TRP_V_5 PLLRASKEND LSVLRQLLDN LEAALVLMEP EGQTALHIAV VNQNVNLVRA LLTRRSVSSF
TRPV5_Mmus   _NP_001007 573_1_TRP_ PLLRAAKEND MCTLKKLQDN LDAAIMLMEP YGQTALHIAV MNQNVNLVRA LLARGSASSF
TRPV6_Hsap   _NP_061116 _2_TRP_V_6 PLLLAAKDND VQALNKLLDN LEAAMVLMEP EGQTALHIAV VNQNMNLVRA LLARRSVSSF
TRPV6_Mmus   _NP_071858 _2_TRP_V_6 PLLLAAKEND VQALSKLLDN LEAAMVLMEP EGQTALHIAV INQNVNLVRA LLARGSVSSF
TRPV6_Drer   _NP_001001 849_1_TRP_ PLFSATKENN AACIKKLLDN FEAAVALMNP EGLTALHIAV INQNPNLIWE MIKRGDVTAF
TRPML1_Hsa   p_NP_06539 4_1_mucoli KLMLQVVKIL VVTVQLIILNT IAFRHLFLLS DADDTF--AA YTQLYQAIFH AVDQYALPAL
TRPML1_Mmu   s_NP_44440 7_1_mucoli KLMLQVVKIL VVTVQLIILNT IAFRHLFLLS DSDDTF--AA YTQLYQAIFY AVDQYILPAL
```

```
TRPML1_Dre  r_XP_00133 6199_1_PRE KLALQLLKII IVTVQLVMNT DSFKHLFLRV ESQEPL--CL HTQVHEHIRY AIDQYILSYL
TRPML2_Hsa  p_NP_69499 1_2_mucoli KLGLQILKIV MVTTQLVRNT VAFKHLFLKS GDEDDYSCSV YTDAYESIFF AINQYQLKKV
TRPML2_Mmu  s_NP_08093 2_2_mucoli KLGLQILKIV MVTTQLVRNT VAFKHLFLKS GDEDDYSCSI YTNTYESIFF AIKQYHLKKV
TRPML2_Dre  r_NP_95744 2_1_mucoli KLAVQIFKIF MITLQLILNL MAFKNLFLKS GDEDEYSISI YTRVFDSLHY VLDQYQLQII
TRPML3_Hsa  p_NP_06076 8_8_mucoli KLAIQILKIA MVTIQLVLNT IAFKHLFLKM DMDDTY--AV YTDVYDQLIF AVNQYQLYAI
TRPML3_Mmu  s_NP_59892 1_1_mucoli KLAIQILKIA MVTIQLVLNT IAFKHLFLKM DMDDTY--AV YTEVYDQIIF AVTQYQLQAI
TRPML3_Dre  r_XP_00134 1182_4_PRE KLFLQIVKIA VVTVQLVSNL LTFRHLFLKS ENTNTY--AI YTDVYTHITH IVQQFMLPCI
TRPML3_Dme  l_NP_00116 3472_1_CG4 KFVVQIVKIF LVTMQLCLNR FAFSHLFLRD SAVGPF--AL YLEFFDTVQY AVNGYNVSKL
TRPML3_Dme  l_NP_64914 5_1_iso_mu KFVVQIVKIF LVTMQLCLNR FAFSHLFLRD SAVGPF--AL YLEFFDTVQY AVNGYNVSKL
TRPML3_Cel  e_NP_49866 4_3_CUP_5_ KLVLQVLKIV FVTMQLILTT TVMRHRFLKN DAEGRY--SV YDGLSEHLSF LINSYSIREV
TRPP_PKD1_  Hsap_NP_00 1009944_2_ DMSLAVEQGA PVVVSAAVVQ LRATNMLGSA DFVEPVGWLM VAPNPAAVNT SVTLSELATQ
TRPP_PKD1_  Mmus_NP_03 8658_2_pol YLSPSVEQGA PMVVSASVVH LRATNMLGSA NFVEPVESLI LSPNPAAVNM SLTLCELAAR
TRPP_PKD1_  Drer_XP_00 3197787_1_ MSSAPTEHNT PTVIRASLVI LKATNLLGQV NFLDPVQDLV LEPNPAAVNA MTNMTSVNRK
TRPP_PKD2_  Hsap_NP_00 0288_1_pol FLDTPVSKTE KTNFKTLS-- ---------- -YENLLLGVP RIRQLRVRNG SCSIPDLREI
TRPP_PKD2_  Mmus_NP_03 2887_3_pol FIDTPVSKTE KTNFKTLS-- ---------- -YENLLLGVP RLRQLRVRNG SCSIPDLREI
TRPP_PKD2_  Drer_NP_00 1002310_1_ FLDTPLSSGE PTNFKSLS-- ---------- -YENLLLGVP RLRQLRVRNE SCSVHDLREV
TRPP_PKD2_  Cele_NP_50 2838_3_PKD FVASTGASG- APAFGSCT-- ---------- -YENRLLGEP RIRMLKVTND SCTVMSFQEI
TRPP_PKD2L  1_Hsap_NP_ 057196_2_P FLHTPSDTG- -VSFQAIS-- ---------- -YENMLLGVP RLRQLKVRND SCVVHDFRDI
TRPP_PKD2L  1_Clup_XP_ 543967_3_P FLHTPSDTG- -VSFQAIS-- ---------- -YENLLLGVP RLRQLRVRND SCVVHDFRDI
TRPP_PKD2L  1_Mmus_NP_ 852087_2_P FLHTPSDSG- -VSFQTIS-- ---------- -YENLLLGAP RLRQLRVRND SCVVHDFRDI
TRPP_PKD2L  1_Drer_XP_ 695404_2_P FVNLPGSNG- -MSFSSIG-- ---------- -YENLLLGVP RVRQLKVKQN SCKVPDFKEI
TRPP_PKD2L  2_Hsap_NP_ 055201_2_P FLDTSVPGEE RTNFKSIR-- ---------- -YENILLGVP RVRQLKVRNN TCKVYSFQLM
TRPP_PKD2L  2_Mmus_NP_ 001156476_ FVDTSLPDDE RSSFRSIR-- ---------- -YENVLLGIP RVRQLRVRNN TCKVYAFQLV
TRPP_PKD2L  2_Dmel_NP_ 609561_2_P NREMVVAPSV TVGFEKLISD SIQANMSFHE GYENLLLGPP RLRQIRVRKE SCYVNAFIYF
Cca7050     LFLHACVHGS KATLQELLLE LAIAARVDHQ DGSTPLHYAC AAENDAVISL LIEAGDMNNL SIKSQHIYQD LYTLFERMAK
Cca21784    ---------- --------MQ TAIDKGVDHS DGNTPLHYAA AADNHEVIEL LIGDADTNCF AVLSNQRIEK LFVLYRHWGY
Cca260897_  1_PKD2FVNV KLESGSSSTF ESIT------ -------YEN KLLGRPRLRL LRVRSDSCSI HEFKEITDCY GDSRDYWATG
Sla36613_6  ---------- ---------- ---------- ---------- ---------- ---------- ---------- ----------
Sycon24227  _2_6SAQQPR RESNYSRVTE VSAEEAVMNI VENSQ----- ------EARH LLDGLIARGD LG-------- FVYVAHDAMR
Sycon24228  _3SAQQPRRE SNYSRVTEVS AEEAVMNIVE NSQ------- ----EARHLL DGLIARGDLG --------FV YVAHDAMRKM
Sycon9763_  1--------- ---------- ---------- ---------- ---------- ---------- ---------- ----------
Ava16635_4  PIVRIVIDNT LHVIEQVGVD LAEQNIFTEV WLFQHYFAIL ILNQVDFLEL MLERNIDHNL ERLYFVNLFI WNAAIAGMHH
Ava9904_1   PAVCLLLDNS IDGLRHTLPS LALEKIFIGK WVQRIFFSAL TTNNTGFVHR MLESK---YL SQLYKRQVFI WTVAIRGMKL
Ava7536_5   ---------- --------MA FVSKYALDQS TRKQVFHLNL LETNRDLTR- ---------- ---------- ----------
Ava6620_1   -IRYALFYGS REIVENIFNN SDIQQRLIKE ELFQRLMFAL TKDEEYVEKK IKGCKEFTSL EVVKFLYIMV WNAILYGVEQ
Cca31351    DIGVQEEVGQ MDTARTFS-- ---------- -----VEEKL TSFDESFVKF MNGNENVDVF KEKGGLALEM FTARLNVISL
Cca30979_3  DIVLKANGGS GIAFSDVS-- ---------- -PYLKLVGKP RLKQFRVEKG SCTVPILAEV TRCYNSTWLI WGTGVVDLDR
Ava26193_2  VLHAKNREGD TPFELAVN-- ---------- -KQDAMAAYL AGPDYEVRRL FTASGDPS-- ---------- ----------
Ava26160_2  ---------- ---------- ---------- ---------- ---------- ---------- ---------- ----------

TRPC1_Hsap  AAHRNNRLDI YRALLKELSL VIKYNQKEFV SQSNCQQFLN TVWIHTPFMK FIIHGASYFT FLLLLNLYRI DYLLILWIIG
TRPC1_Mmus  HRNNRLDIYR ALLKELSLVI KYNQKEFVSQ SNCQQFLNTV WIHTPFMKFI IHGASYFTFL LLLNLYRIDY LLILWIIGMI
TRPC1_Drer  AAHRNNRLDI YRALLKELSL VIKYNQKEFV AQSNCQQFLN TVWIHTPFIK FIIHSASYFT FLLLLNLYFI DYLLILWIIG
TRPC2_Mmus  ACQKDLRINT YAALLRRLAR KVNYNQKQFV AHPICQQVLS SIWLKIPVLK FLLHSASYLW FLIFLLGEVW ESLHMIWVTG
TRPC2_Drer  ACQKDLRINT YSALLRKLSK KVNYNQKQFV AHPICQQVLS SIWLKIPVIK FLLHSASYMW FLITLLGEIL HSFHMVWVVG
TRPC2_Drer  ACQKDLRINT YSALLRKLSK KVNYNQKQFV THPICQQVLS SIWLKVPVIK FLLHSASYMW FLIALLTEIL HSLHMVWVAG
TRPC3_Hsap  AAHCQKRINA YTALLAKLAN IIKYEVKKFV AHPNCQQQLL TIWLRSPFMK FVAHAASFII FLGLLVFNWT EMLIMVWVLG
TRPC3_Mmus  HCHKRINAYT ALLAKLANII KYEVKKFVAH PNCQQQLLTI WLRSPFMKFV AHAASFIIFL GLLVFNWTEM LIMVWVLGMM
TRPC4_Hsap  AAHTNNRLNI YTALLQELSK VIKYRQKEFV AQPNCQQLLA SRWIRKPFIK FICHTASYLT FLFLLLLAIV EWMILPWVLG
TRPC4_Mmus  AAHTNNRLNI YTALLQELSK VIKYRQKEFV AQPNCQQLLA SRWIRKPFIK FICHTASYLT FLFLLLLAIV EWMILPWVLG
TRPC4_Drer  AAHTNNRLNI YTALLQELSK VIKYRQKEFV AQPNCQQLLA SRWIRKPFIK FICHTASYLT FLFLLLLATV EWMILPWVLG
TRPC4_Dmel  AAHRDNRINA YTALLRRLSF LIKLRQKKFV AHSNVQQLLA SIWMRKPFIK FICHSASYFT FLFLLMLAFI EWLILAWVSG
TRPC4_Cele  AAHKDNRINA YYALLKRLSF IIQLSQKRFV AHPNCQQLLL DIWAKKPFIK FLSHSGSYIF FLILLIMAII ECAIFLWVLG
TRPC5_Hsap  HTNNRLNIYT ALLKELSKVI KYHQKEFVAQ PNCQQLLATL WIKKPFIKFI CHTASYLTFL FMLLLAVVEW MILPWVLGFI
TRPC5_Mmus  HTNNRLNIYT ALLKELSKVI KYHQKEFVAQ PNCQQLLATL WIKKPFIKFI CHTASYLTFL FMLLLAVVEW MILPWVLGFI
TRPC6_Hsap  HCQERINAYT ALLAVLANII KYEVKKFVAH PNCQQQLLSI WMRGPFMKFV AHAASFTIFL GLLVMNWMEM LIISWVIGMI
TRPC6_Mmus  HCQERINAYT ALLAVLANII KYEVKKFVAH PNCQQQLLSI WLRGPFMKFV AHAASFTIFL GLLVMNWMEM LIISWVIGMI
TRPC6_Drer  ASHCHERINA YAALLASLAN IIKYELKKFV AHPNCQQQLL SIWMRGPFLK FVAHAASFTI FLGLLVMNWM EMLIISWVIG
TRPC6_Drer  ASHCHERINA YTALLAVLAN IIKYELKKFV AHPNCQQQLL SIWIRGPFLK FVAHAASFTI FLGLLIMNWM EMLIISWVVG
TRPC6_Cele  AAQLNQRINT FSALLQRLAF EIKYEQKAFV SHPHCQQLLT SIWVRSPFMK FFYYSVSFAT FLGLLTWAWV ESLVFTWVIG
TRPC7_Hsap  AAHCQERMNA YTALLARLAN IIKYEVKKFV AHPNCQQQLL TMWLRSPFMK FVAHAVSFTI FLGLLVVNWT EMLIMKWVLG
TRPC7_Mmus  HCQERMNAYT ALLARLANII KYEVKKFVAH PNCQQQLLTM WLRSPFMKFV AHAVSFTIFL GLLVVNWTEM LIMKWVLGMI
TRPC7_Drer  AAHCKERMNA YTALLAQLAN IIKYEVKKFV AHPNCQQRLL TLWLRSPFMK FVAHAVSFTL FLCLLVLNWT EMLIMKWVLG
TRPN_Dmel_  AAMNGFHMSV VTALYIVLST KIENEQKEVI AHTVVQRYLQ ELWNKVPIIK FMSYLTSHIY LMIHLSIVWY EVGLLIWLSG
TRPN_Cele_  AAQHGHHIAV VTALYRDMSE KIENEQKEVV SYASVQRYLT EVWGRAPIIK FVCHIVSHVY FTILLTIVPV EWLLLLWLSG
TRPN_Drer_  SAQNGSHTAV VTALLTLCAL RIEGRQKGVV SQPAVQTYLT EVWNTIPIVK FMSHLVSHIF LLTLFILTWS ECLLLIWLCG
TRPM1_Hsap  EELYNTELMV WKAAYKAMAH EVAAKHRDFI AHTCSQMLLT DMWYNAPIVK FWFYTISYLG YLLLFNYVLQ EWIVISYIVS
TRPM1_Mmus  EELYNTELMV WKAAYKAMAH EVAAKHRDFI AHTCSQMLLT DMWYNAPIVK FWFYTISYLG YLLLFNYVPQ EWIVISYIVS
TRPM2_Hsap  LYLYENDLLI WAACLKELSK ELEAKDMKFV SHGGIQAFLT KVWFTAPVVV FHLNILSYFA FLCLFAYVWC ECAIYLWLFS
TRPM2_Mmus  LCLYENDLLI WAACLKELSK ELEAKDMKFV SHGGIQAFLT KVWFNAPVVI FHMNILSYFA FLCLFAYVWC EYLIYLWLFS
TRPM3_Hsap  EELYNTELMV WKAACKAMAH EVAAKHRDFI AHTCSQMLLT DMWYNAPIVK FWFYTLAYIG YLMLFNYITQ EWIVISYIFT
TRPM3_Mmus  EELYNTELMV WKAACKAMAH EVAAKHRDFI AHTCSQMLLT DMWYNAPIVK FWFYTLAYIG YLMLFNYITQ EWIVISYIFT
TRPM3_Drer  EELYNTELMV WKAACKAMAH EVAAKHRDFI AHTCSQMLLT DMWYNAPIVK FWFHTLAYVG YLMLFNYIPQ EWIVIAYIFT
TRPM3_Dmel  EELYNTELLI WKSSYKAMAH EVAANHRALL AHPCSQVILA DLWYTAPITK FWADSIAYMF FLIMFSFTWQ EWYSIAYITT
TRPM3_Cele  DELYNTELMI WKAAYKSLAT EVIVNNKHFL AHPCCQILLA DLWYSAPIST FWSWTISFIL FITFFTYTVI EYILIAYVAA
TRPM3_Cele  LYNTDLMIWK AAYVSLAKTA NNGHRKFLAH PCCQMLLSDL WYKAPITTYW LWFFAFIWFL ILLTYNWSEW YVFAYIFVWT
TRPM3_Cele  LYNMDLLIWK CAYKATAKIV LANTKTFLAH PCCQILLAEL WYSSPITKFW SWCIAFLIFL TTQTCIKYEW ITFIYTVTLS
```

TRPM4_Hsap	AQLYSADLLL	WSAALRVMAR	LMQADARAFF	AQDGVQSLLT	QKWWGAPVTI	FMGNVVSYLL	FLLLFSRVSL	ELLLYFWAFT		
TRPM4_Mmus	AQLYSADLLI	WSAALRVMAR	LMQADARAFF	AQDGVQSLLT	QKWWGAPVTA	FLGNVVSYLL	FLLLFAHVVS	ELLLYFWAFT		
TRPM4_Drer	EKLYNSSLFI	WTAGLVELSK	QIAADARIFF	SHDGVQSLLS	QIWWYAPVTS	FIGNLLMYFL	FLFLYAYVVS	EYVLYFWVFT		
TRPM4_Drer	EILYNSCLFI	WTAGLLELSK	QIAADARIFF	SHDGVQSLLS	QIWWYAPVTS	FIGNLLMYFL	FLFLYAYVVS	EYVLYFWVFT		
TRPM4_Drer	EKLYNSCLFI	WTAGLLELSK	QIAADAQIFF	SHDGVQSLLS	QIWWYAPVTS	FIGNLLMYFL	FLFLYAYVVS	EYVLYFWVFT		
TRPM4_Drer	ESLYSSSLFI	WSAGLRELSK	LTSADARLFF	SHDGVQTLLS	EIWWFAPVTS	FLGNVLMYFL	FLFLFAYVPL	ELVLYFWVFT		
TRPM5_Hsap	QELYRSDLFL	WAAALKEMSH	LTEADAKAFF	AHDGVQAFLT	RIWWGAPVTV	FLGNVVMYFA	FLFLFTYVGP	EVTLYFWVFT		
TRPM5_Mmus	QQLYHSDLFL	WAAAIKEMSH	LTEADAKAFF	AHDGVQAFLT	KIWWGAPVTV	FLGNVVMYFA	FLFLFTYVGS	EVTLYFWVFT		
TRPM5_Drer	QELYCSDLFL	WAAGMKEMAH	LTLAEAKCFF	AHDGVQALLT	KVWWSAPVTV	FMGNVIMYFA	FLILFSYVAA	EIILYFWVFT		
TRPM6_Hsap	EELYNTDLLV	WKAAYRAMAH	EVSGGLRPFV	SHTCTQMLLT	DMWYSAPIVK	FWFYTMAYLA	FLMLFTYTVQ	EWLVSIYIFT		
TRPM6_Mmus	EELYNTDLLV	WKAAYRAMAR	EVSGGLRPFV	SHSCTQMLLT	DMWYSAPFVK	FWFYTMAYLA	FLMLFTYTVH	EWLVIIYIFT		
TRPM7_Hsap	EELYNTELLI	WKAAYRSMAY	EVSSRLRPFV	AHTCTQMLLS	DMWYHAPIVK	FWFNTLAYLG	FLMLYTFVVQ	EWIVIAYIFT		
TRPM7_Mmus	EELYNTELLI	WKAAYRSMAY	EVSSRLRPFV	AHTCTQMLLS	DMWYHAPIVK	FWFNTLAYLG	FLMLYTFVVQ	EWIVIAYIFT		
TRPM7_Drer	EELYNTELLV	WKAALRSLGD	EVSSRLRPFV	AHTCTQMLLS	DMWYHAPIVK	FWFNTLFYIG	FLMLYSFVPQ	EWVVILYIFT		
TRPM8_Hsap	TELFSNALFI	WAAALKTLAK	VVEATDQHFI	AQPGVQNFLS	KQWFTSPFVV	FSWNVVFYIA	FLLLFAYVPP	ELVLYSLVFV		
TRPM8_Mmus	TELFSTALFI	WAAALKTLAK	VVEATDQHFI	AQPGVQNFLS	KQWFTSPFVV	FSWNVVFYIA	FLLLFAYVTP	ELILYALVFV		
TRPA_Hsap_	TASASHDKVV	NKAHLALHNK	VQNNRIELLN	HPVCKEYLLM	KWLAYGFRAH	MMNLGSYCLG	LIPMTILTCM	ILVFLSSIFG		
TRPA_Mmus_	TASASHDKVV	NKAHIALHNK	VQHNRIELLN	HPVCREYLLM	KWCAYGFRAH	MMNLGSYCLG	LIPMTLLICM	ILVFLSSIFG		
TRPA_Drer_	ATSCGAHVKV	VNKAHEAVHN	AVNFNRVNLL	THPVCKKYLE	MKWSAYGIKA	HLLNMTVYAL	GVFPLTYLSS	MLLVLAMNMY		
TRPA_Drer_	ASSCSAHAQV	VNDNHEAVRN	EVRYNRLELL	IHPLSRKYLE	MKWTAYGSKV	HFLNLAIYLL	GLLPLTYLVC	IIMVIVMNVY		
TRPA_Dmel_	AASRSGHTRV	VNVMDYAIYY	KVTHGRVELL	AHPLSQKYLQ	MKWNSYGKYF	HLANLLIYSI	FLVFVTIYFC	AVVIVVYILL		
TRPA_Cele_	THDSHDATTD	EKDIACENDA	DAEKLHLLNH	PLSKALLKYK	WNRLGRPMYY	FALFMYLVFI	VSLTQYVWKI	IIQTLAVCQI		
TRPV1_Hsap	AACTNQKIGV	LTCKLEVIAY	SETPNRHDML	LVEPLNRLLQ	DKWDRFVKRI	FYFNFLVYCL	YMIIFTMATG	EILSVLGGVY		
TRPV1_Mmus	AACTNQKIGV	LTCKLEVIAY	SETPNRHDML	LVEPLNRLLQ	DKWDRFVKRI	FYFNFFVYCL	YMIIFTTATG	EILSVSGGVY		
TRPV1_Drer	AACTNQKLGL	FTYKLEIVVY	GEIPNRLEML	QIEPLNRLIE	EKWDQFAHRM	FLFNFIVYVI	YLFIFTASTG	HIISITGAFY		
TRPV2_Hsap	AACTKQKIEI	FSCELEIIAF	HKSPHRHRMV	VLEPLNKLLQ	AKW-DLLIPK	FFLNFLCNLI	YMFIFTAVTG	HILILLGGIY		
TRPV2_Mmus	AACTKQKIEI	FSWKLEIIAF	HKSPHRHRMV	VLEPLNKLLQ	EKW-DRLIPR	FFFNFACYLV	YMIIFTIVLG	HILILLGGIY		
TRPV3_Hsap	AACTNQKAEI	LTTDLEITVY	NNIDNRHEML	TLEPLHTLLH	MKWKKFAKHM	FFLSFCFYFF	YNITLTLVLG	RMFVLIWAMC		
TRPV3_Mmus	AACTNQKAEI	LTTDLEIIVY	NNIDNRHEML	TLEPLHTLLH	TKWKKFAKYM	FFLSFCFYFF	YNITLTLVLG	RMFVLIWATC		
TRPV4_Hsap	AACTNQKIGI	FTCELEILVY	NKIENRHEML	AVEPINELLR	DKWRKFGAVS	FYINVVSYLC	AMVIFTLTAG	EVITLFTGVL		
TRPV4_Clup	AACTNQKIGV	FTCELEILVY	NKIENRHEML	AVEPINELLR	DKWRKFGAVS	FYINVVSYLC	AMVIFTLTAG	EIITLFTGVL		
TRPV4_Mmus	AACTNQKIGV	FTCELEILVY	NKIENRHEML	AVEPINELLR	DKWRKFGAVS	FYINVVSYLC	AMVIFTLTAG	EVITLFTGVL		
TRPV4_Drer	AACTNQKIGV	FTCELEILVY	NKIENRHEML	AVEPINELLR	AKWQKFAAVT	FYISVFSYLV	TMIIFTLVGG	EIITVGSGLF		
TRPV5_Hsap	AACVNSNTVM	FSWELELVVS	SDKREARQIL	EQTPVKELVS	FKWNKYGRPY	FCILAALYLL	YMICFTTCVG	ELVSIVGAVI		
TRPV5_Mmus	AACVGSNTVM	FSWELELVVS	SKKKEARQIL	EQTPVKELVS	LKWKKYGQPY	FCLLGALYIF	YMVCFTTCVG	ELVTVIGAVI		
TRPV6_Hsap	AACVNSNTVM	FSSELELIIT	TKKREARQIL	DQTPVKELVS	LKWKRYGRPY	FCMLGAIYLL	YIICFTMCVG	ELVTVIGAII		
TRPV6_Mmus	AACVGSNIVM	FSSDLELIVT	TKKREARQIL	DQTPVKELVS	LKWKRYGRPY	FCVLGAIYVL	YIICFTMCVG	ELVSIVGAVI		
TRPV6_Drer	AACVGNN--V	FSRELEIIAT	SHKKEARRIL	ELTPVRQLIT	LKWNLYGKHY	FRSLMVIYLV	YISIFTVCIG	EIISLIGAII		
TRPML1_Hsa	CQRYYHNLTL	K---------	-ITFDNKAHS	GRIPISLET-	---VFQ--HG	DNSFRLLFDV	VVILTCSLLC	ASLLRGFLLF		
TRPML1_Mmu	CQRYYHNLTL	K---------	-ITFDNKAHS	GRIPIRLET-	---VSR--HG	DNSFRLLFDV	VVILTCSLLC	ASLLRGFLLF		
TRPML1_Dre	CQRYFKNITL	N---------	-ILFDNKAHS	GKVKLSLEN-	---VSG--HG	DSYARVAFDV	LVAVVCGLLC	GSILKGIMLY		
TRPML2_Hsa	CKQHYKFFRL	E---------	-IIFDNKAHS	GKIKIYFDS-	---IFGSTQK	NAQYVLVFDA	FVIVICLALC	TSIVLALRLF		
TRPML2_Mmu	CKQHYKFFRL	D---------	-ITFDNTAHS	GKIKIYLNS-	---ISGSTQR	STHYLLVFDV	FVIMICLALC	TSIVLALRLF		
TRPML2_Dre	CKKSYKFFEL	D---------	-IDFDNSCHS	GKMKLSLGF-	---ISGTAQK	NTLYLLIFDG	FVILVCLILC	TSIILAVKLF		
TRPML3_Hsa	CQHFYKNLTL	D---------	-ITFDNKAHS	GRIKISLDN-	---VSGSIQK	NTHYMMIFDA	FVILTCLVLC	ISVIRGLQLF		
TRPML3_Mmu	CQHFYRNLSL	D---------	-ITFDNKAHS	GRIKISLDN-	---VSGSIQK	NTHYMMIFDA	FVILTCLALC	ASVIRGLQLF		
TRPML3_Dre	CQQFYHNFTL	H---------	-IQFDNKAHS	GRILVDLDN-	---MTGASAR	NMYMMVLFDA	VVILILSTLC	ISVKAGVLLF		
TRPML3_Dme	CLQNYRDVEV	N---------	-ITFNNRDHD	GQMLLSLDA-	---IS-DANF	DSMLRSVLNI	FVLLTCALLC	TALWRAYLLF		
TRPML3_Dme	CLQNYRDVEV	N---------	-ITFNNRDHD	GQMLLSLDA-	---IS-DANF	DSMLRSVLNI	FVLLTCALLC	TALWRAYLLF		
TRPML3_Cel	LIDRISFLPE	D---------	-IKFDNSRHT	GQVHVTLST-	---KGVGWSF	DTLLIGGTDI	FVLILCILLC	CALIKAHLLY		
TRPP_PKD1_	SIQANVRILM	RWAARSSANS	AVWHDNKGLS	PAWFLQHVIN	AVWSRAVFLE	LTRYSPAVGL	VTLRL---TS	-VCLLLFAVH		
TRPP_PKD1_	SIQANVRILM	RQAAQSSHNP	VVWHDNKGLS	PAWFLQHIIN	AVWSRAVFVE	LTRYSPAVGL	VTLRL---TS	-VCLLLFALY		
TRPP_PKD1_	AACLQLRILM	LTGAKAVLPP	ALWHDNKGLS	PAWLLQYVLA	AVWTRALFLE	FSLYNINTDL	FSFLL---LT	-LILLVLLIY		
TRPP_PKD2_	KECYDVGSSH	WATGYLDLSR	T------REE	TAAQVASLKK	NVWTRATFID	FSVYNANINL	FCVVRLLVAC	EIIFCFFIFY		
TRPP_PKD2_	KECYDVGSSH	WASGYLDLSR	T------REE	TAAQLAGLRR	NFWTRAAFID	FSVYNANINL	FCVVRLLAAC	EIIFCFFIIY		
TRPP_PKD2_	YDCYNVESSY	WSTGYQDLSR	T------REK	SANQLQELKN	NLWTRAVFLD	FSIYNGNVNL	FCIVRLLVMC	EVSFCLFVLY		
TRPP_PKD2_	KECFANNLKT	VASGVQRLPV	A-----GSTE	AQSAIATLKA	NRWSRAIIVD	FALYNANINL	FCVVKLLFIF	EGIFCGFILY		
TRPP_PKD2L	LSCYDVGFSH	WTSGYLDLPG	S------RQG	SAEALRALQE	GLWTRVVFID	FSVYNANINL	FCVLRLVVGC	EVIFCVFIFY		
TRPP_PKD2L	LSCYDVGSSH	WTSGYLDLPG	S------RQA	SAEALQDLQE	GLWTRVVFID	FSVYNANINL	FCVLRLVVGC	EIIFCIFIVY		
TRPP_PKD2L	LNCYDVGSSH	WTSGYLDLPG	S------RQA	SAEALQGLQE	GLWTRVVFID	FSVYNANINL	FCILRLVVGC	EVVFCVFIFY		
TRPP_PKD2L	SDCFDVGSSH	WTTGYQDLTT	T------RDE	SAALLEDLRE	NLWTRVLFID	FTAYNANINL	FCVIRLVVGC	EIVFCVFILY		
TRPP_PKD2L	SECYGKSPWH	WGVNIFTLSK	S------KSE	TKNKFIDLRL	NSWTRVIFID	FSLYNANVNL	FCIIRLVASC	EITFCIFLFV		
TRPP_PKD2L	SDCYSKAPWH	WGVDIVTLSK	S------KSE	TKAKFVDLRL	NNWTRAVFID	FSLYNANVNL	FCIIRLLASC	EVIFCIFLFV		
TRPP_PKD2L	NTCYAASTPI	WAFTTVNLDY	D------KDR	NVKIINDLKD	IHWSRLCLVE	FNLFNENTDI	FQSIKLIAVI	YIFWYIMVIY		
Cca7050	EISTGRKRFI	ATDAVQYELN	RVWRAAPTIR	YRVEAFAYVA	FLILLMVEKL	EWITLAYVLA	LLYNEFVTLV	REGHFYFTRL		
Cca21784	NISDRCKKFI	AHASVLQALK	RSWIAEPIII	YTAHVISYSV	FVVLLLIDGL	LLVIFIFVFG	LILQEIRSFL	RLERFYFSGL		
Cca260897_	THLLPES---	---RRQAARV	IAELKDNMWS	RVVFLDFTVY	NANINLFCVV	KLIVFCEVVF	CLFIIYYIVE	EVLEIYLHRM		
Sla36613_6	----------	----------	----------	----------	----------	----------	----------	----------		
Sycon24227	KMNKKLECKD	KELQRHNVVR	MLIYNKWVEY	GERMAILNLG	LYLLFMLCVT	FSVLEVISLL	GVMVNLYDEA	KECYREKTAY		
Sycon24228	NKKLECKDKE	LQRHNVVRML	IYNKWVEYGE	RMAILNLGLY	LLFMLCVTFS	VLEVISLLGV	MVNLYDEAKE	CYREKTAYVT		
Sycon9763_	----------	----------	----------	----------	----------	----------	----------	----------		
Ava16635_4	HVTGRCMTFL	SHPCVQSLLD	EQWYTSPSIK	FITYVLAFIL	YLTLYSFV--	EWIFIVWSLT	YILEEVRQLF	AGNSAYLNDR		
Ava9904_1	KIKGDCKHFI	AHPLIQDYAN	QKWYNAPKIK	YWLHFLSHLV	YTILFTIVII	ELIVWVWTFT	IIMEECSQLL	HEKRGYISSA		
Ava7536_5	-VQFKRFELV	LHPIFKKLLE	MKWRKYGFLF	AIIQTVFYFT	FWSLFTIH--	-FVCIAFLVF	QIVEEFTEIF	EQNNPYTADF		
Ava6620_1	ESTAGKQLFF	THEGVQGHVR	NVWYISPSTT	FYIHSLVYFI	FLVLYTVHTF	DVIYFLYMVA	YILDEVFQIE	DKTFEYLSSG		
Cca31351	E--------F	SHTPMEKMVT	SVW-------	----------	FWLIPPTP--	-VIYEKWVKI	FLGDMVEKHL	YEG-------		
Cca30979_3	N-----NRSI	AGETISGLQE	SRWTRAVTVQ	FTVYNANSNL	FSIGLLLVAC	EAAFVFFLFY	YTVKAIRAIH	KVKGAFIVEP		

```
Ava26193_2    -ARSGYEEIV YHDTVRLLLR MKWKKFGRFR FLLQFLTYLL HILAMSVSLC EIVYYVFLLW NLAAEIFQMF RHRLRYLLDW
Ava26160_2    ---------- ---------- ---------- ---------- ---------- ---------- ---------- ----------

TRPC1_Hsap    MIWSDIKRLW YEGEDFLEES RNQLSFVMNS LYLATFALKV VATLVAEGLF AFANVLSYLR LFFMYTTSSI LGPLQISMGQ
TRPC1_Mmus    WSDIKRLWYE GEDFLEESRN QLSFVMNSLY LATFALKVVA TLVAEGLFAF ANVLSYLRLF FMYTTSSILG PLQISMGQML
TRPC1_Drer    MVWSDVKRLW YEGEDFLEES RNQLSFVMNS LYLATFALKI VATLVAEGLF AFAIVLSYLR LFFMYTTSSI LGPLQISMGQ
TRPC2_Mmus    FLWFECKEVW IEGRSYLLDW WNFLDVVILS LYLASFALRL LLQFLAEVLF AVTSMLSFTR LAYILPAHES LGTLQISIGK
TRPC2_Drer    FFWFECKEVW IEGRSYFLDW WNCLDVMVLS MYLASFALRL VIQFISEVLF AVTSMLSFTR LAYILPAHES LGTLQISIGK
TRPC2_Drer    FFWFECKEVW IEGKSYFLDL WNILDMMVLS MYLASFTLRI LIQLIAETLF AVTSMLSFTR LAYILPAHES LGTLQISMGR
TRPC3_Hsap    MMWSECKELW LEGREYILQL WNVLDFGMLS IFIAAFTARF LAQIISEGLY AIAVVLSFSR IAYILPANES FGPLQISLGR
TRPC3_Mmus    WSECKELWLE GREYIVQLWN VLDFGMLSIF IAAFTARFLA QIISEGLYAI AVVLSFSRIA YILPANESFG PLQISLGRTV
TRPC4_Hsap    FIWGEIKQMW DGGQDYIHDW WNLMDFVMNS LYLATISLKI VATLVAEALF AIANIFSSLR LISLFTANSH LGPLQISLGR
TRPC4_Mmus    FIWGEIKQMW DGGQDYIHDW WNLMDFVMNS LYLATISLKI VATLVAEALF AIANIFSSLR LISLFTANSH LGPLQISLGR
TRPC4_Drer    FIWTEIKQMW DGGQDYIHDW WNLMDFVMNS LYLATISLKI VATLVAEAVF AIANIFSSLR LISLFTANSH LGPLQISLGR
TRPC4_Dmel    LIWSEVKQLW DVGQEYLNDM WNVIDFVTNS LYVATVALRV VSMLISEGLF SAANIFSSLK LVYIFSVNPH LGPLQVSLSR
TRPC4_Cele    LIWVEIKQLW ECGYNYCRNL WNILDFITNS LYLCTTALRV VATLLSECFF ATANIFSSLK LVHIFTVSPH LGPLKISLGR
TRPC5_Hsap    WGEIKEMWDG GTEYIHDWWN LMDFAMNSLY LATISLKIVA TLIAEALFAI SNILSSLRLI SLFTANSHLG PLQISLGRML
TRPC5_Mmus    WGEIKEMWDG GTEYIHDWWN LMDFAMNSLY LATISLKIVA TLIAEALFAI SNILSSLRLI SLFTANSHLG PLQISLGRML
TRPC6_Hsap    WAECKEIWTQ GKEYLFELWN MLDFGMLAIF AASFIARFMA QIISEGLYAI AVVLSFSRIA YILPANESFG PLQISLGRTV
TRPC6_Mmus    WAECKEIWTQ GKEYLFELWN MLDFGMLAIF AASFIARFMA QIISEGLYAI AVVLSFSRIA YILPANESFG PLQISLGRTV
TRPC6_Drer    MIWAECKEIW SQGREYLLEP WNLLDFGMLA IFVASFISRI MAQLVSEGLY AIAVVLSFSR IAYILPANES FGPLQISLGR
TRPC6_Drer    MIWAECKEIW SLGREYLLEP WNLLDFGMLA IFVTSFIARF MAQLISEGLY AIAVVLSFSR IAYILPANES FGPLQISLGR
TRPC6_Cele    MLWSEIKQLW EEGKRYMRQW WNWLDFLMIC LYLCTISIRL SAMLVAEALF AVGNVFSFAR IIYLFQTNPY LGPLQISLGC
TRPC7_Hsap    MIWSECKEIW EEGREYVLHL WNLLDFGMLS IFVASFTARF MAQIISEGLY AIAVVLSFSR IAYILPANES FGPLQISLGR
TRPC7_Mmus    WSECKEIWEE GREYVLHLWN LLDFGMLSIF VASFTARFMA QIISEGLYAI AVVLSFSRIA YILPANESFG PLQISLGRTV
TRPC7_Drer    MIWTECKEVW ADGREYIMHL WNVLDFGMLS VFVASFTARL MAQLISEGLY SIAVVLSFSR IAYVLPANES FGPLQISLGR
TRPN_Dmel_    LLLFELTNPS DKS------G LGSIKVLVLL LGMAGVGVHV SAVYCRNQCF ALAFLLACVQ ILDFLSFHHL FGPWAIIIGD
TRPN_Cele_    NLVSELSTVG GGS------G LGIVKVLILV LSAMAIAVHV LALYLKNQLF AFALLFAFVE YLDFLTVHHL FGPWAIIIRD
TRPN_Drer_    MLVSELTFPG ERT------G LAWIRLLLLG FSAAALLCHL LALFARNVLL AVAMTLGFIQ LLEFLTFHHL FGPWAIIIRD
TRPM1_Hsap    LALEKIREIL MSEKVWLQEY WNITDLVAIS TFMIGAILRL QNMGYGRVIY CVDIIFWYIR VLDIFGVNKY LGPYVMMIGK
TRPM1_Mmus    LALEKIREIL MSEKVWLQEY WNITDLVAIS MFMVGAILRL QSMGYGRVIY CVDIILWYIR VLDIFGVNKY LGPYVMMIGK
TRPM2_Hsap    LVCEEMRQLF YDPALYFSDF WNKLDVGAIL LFVAGLTCRL IPLYPGRVIL SLDFILFCLR LMHIFTISKT LGPKIIIVKR
TRPM2_Mmus    LVCEETRQLF YDPSLYFSDF WNKLDVGAIL LFIVGLTCRL IPLYPGRIIL SLDFIMFCLR LMHIFTISKT LGPKIIIVKR
TRPM3_Hsap    LGIEKMREIL MSEKVWLQEY WNVTDLIAIL LFSVGMILRL QDRSDGRVIY CVNIIYWYIR LLDIFGVNKY LGPYVMMIGK
TRPM3_Mmus    LGIEKMREIL MSEKVWLQEY WNVTDLIAIL LFSVGMILRL QDRSDGRVIY CVNIIYWYIR LLDIFGVNKY LGPYVMMIGK
TRPM3_Drer    NGIEKMREIL MSEKVWLQEY WNITDLMAIL IFSIGMVLRL QDMSYGRVIY CVNIIYWYIR LLDIFGVNKY LGPYVMMIGK
TRPM3_Dmel    LGFEKVREII SSESVWAWNM WNPCDGAAII LFVIGLAFRF REMDIGRVIY CVDSIYWYLR ILNILGVNKY LGPLVTMMGK
TRPM3_Cele    FGLEQVRKII MSDRTYVCSF WNCVTILAII FYIVGFFMRC FGVAYGRVIL ACDSVLWTMK LLDYMSVHPK LGPYVTMAGK
TRPM3_Cele    LEIGRKIMMD TRVFFFQYRN GLLAFGLLTY LIAYFIRLSP KTLGRILIIC NSVIWSLKLV DYLSVQQGLG PYINIVAEMI
TRPM3_Cele    VEHIRKLMTS EKVFYAKWYN IWTSAALLFF LVGYGFRLVP HSWGRVLLSF SNVLFYMKIF EYLSVHPLLG PYIQMAAKMV
TRPM4_Hsap    LLCEELRQGL SGGRLYLADS WNQCDLVALT CFLLGVGCRL TPYHLGRTVL CIDFMVFTVR LLHIFTVNKQ LGPKIVIVSK
TRPM4_Mmus    LLCEELRQGL GGGHLYLSDT WNQCDLLALT CFLLGVGCRL TPFDLGRTVL CLDFMIFTLR LLHIFTVNKQ LGPKIVIVSK
TRPM4_Drer    LVCEEIQEAS IAGIVYAQDM WNKFDVLAIC LFIAGLCCRM FSFNMGRGIL CVDYMVFTLR LIHIFAIHKE LGPKIIILGK
TRPM4_Drer    LVCEEIREAS IAGFVYAQDM WNKFDVLAIS LFITGLCCRM FSFNMGRGIL CVDYMVFTLR LIHIFAIHRQ LGPKIIILGK
TRPM4_Drer    LVCQEIQEAF IAGFLYAQDV WNKFDVLAIS LFITGLCCRM FSFNMGRGIL CVDYMVFTLR LIYIFAIHKE LGPKIIILGK
TRPM4_Drer    LACEEIRQSF FVGKVYIQDI WNKCDITALG LFVLGLLCRM FPYNFGRAVM CVDYTVFTLR LIHIFAVHKQ LGPKIIIVGK
TRPM5_Hsap    LVLEEIRQGF FTDTLYVGDN WNKCDMVAIF LFIVGVTCRM LPFEAGRTVL AMDFMVFTLR LIHIFAIHKQ LGPKIIVVER
TRPM5_Mmus    LVLEEIRQGF FTDTLYVEDN WNKCDMVAIF LFIVGVTCRM VPFEAGRTVL AIDFMVFTLR LIHIFAIHKQ LGPKIIIVER
TRPM5_Drer    LVLEEIRQSF FTDKLYVEDN WNKCDMVAIS LFVVGLSCRM AMYEAGRTVL ALDFMVFTLR LIHIFAIHKQ LGPKIIIVER
TRPM6_Hsap    NAIEVVREIC ISEKVWISEY WNLTETVAIG LFSAGFVLRW GDHTAGRLIY CIDIIFWFSR LLDFFAVNQH AGPYVTMIAK
TRPM6_Mmus    NAIEKVREIC ISEKMWLSEY WNLMETVAIG LFAVGFGLRW GHQTAGRLIY CIDIIFWFSR LMDFFAVNQH AGPYVTMIAK
TRPM7_Hsap    YAIEKVREIF MSEKVWFSDY FNISDTIAII SFFIGFGLRF GAFVAGRLIY CLNIIFWYVR LLDFLAVNQQ AGPYVMMIGK
TRPM7_Mmus    YAIEKVREVF MSEKVWFSDY FNVSDTIAII SFFVGFGLRF GAFVAGRLIY CLNIIFWYVR LLDFLAVNQQ AGPYVMMIGK
TRPM7_Drer    LAVEKIREMF MSEKVWFSDY FNISDFLAIL MFFVGFGLRL VSFIAGRIVY CLNIIFWYVR LLDILAVNQQ AGPYVMMIGK
TRPM8_Hsap    LFCDEVRQWY VNGVNYFTDL WNVMDTLGLF YFIAGIVFRL HSLYSGRVIF CLDYIIFTLR LIHIFTVSRN LGPKIIMLQR
TRPM8_Mmus    LFCDEVRQWY MNGVNYFTDL WNVMDTLGLF YFIAGIVFRL HSLYSGRVIF CLDYIIFTLR LIHIFTVSRN LGPKIIMLQR
TRPA_Hsap_    YCKEAGQIFQ QKRNYFMDIS NVLEWIIYTT GIIFVLPL-- --HLQWQCGA IAVYFYWMNF LLYLQRFENC GIFIVMLEVI
TRPA_Mmus_    YCKEVIQIFQ QKRNYFLDYN NALEWVIYTT SIIFVLPL-- --YMQWQCGA IAIFFYWMNF LLYLQRFENC GIFIVMLEVI
TRPA_Drer_    AVGKEILQMF QQRLNYLRDL SNYMDWAAAI CALLFVVPL- ---SWHWQAG ALAALTSWLN LLLYLQRFER IGIYVVMFRE
TRPA_Drer_    SICKEVVQLA QQRVNYFTDF SNPADWSAAI SALVFVVPM- ---TWQWEAG AYAILTSWIG FLLYFQRFER IGIYVVMFNG
TRPA_Dmel_    NSMRELIQIY QQKLHYILET VNLISWVLYI SALVMVTPA- --NTIHYSAA SIAVFLSWFR LLLFLQRFDQ VGIYVVMFLE
TRPA_Cele_    LVECFQLFQR KFAYLVNWEN WIDCFIYSTA LITVYDFSEC QNWQWILAAL CIFFGWINLL FMIRKMPRFG IFVVMFVDIV
TRPV1_Hsap    FFFRGIQYFL QRRTLFVDSY SEMLFFLQSL FMLATVVLYF SHLKEYVASM VFSLALGWTN MLYYTRGFQQ MGIYAVMIEK
TRPV1_Mmus    FFFRGIQYFL QRRSLFVDSY SEILFFVQSL FMLVSVVLYF SHRKEYVASM VFSLAMGWTN MLYYTRGFQQ MGIYAVMIEK
TRPV1_Drer    FFIRGLIDMV RKRSLIIDGY TDQLFFVQGL LFLASVVLYC YGQYEYLAFL VLCLALSWIN LLYFSRGSKN LGIYNVMIQK
TRPV2_Hsap    LLVGQLWYFW RRHISFIDSY FEILFLFQAL LTVVSQVLCF LAIEWYLPLL VSALVLGWLN LLYYTRGFQH TGIYSVMIQK
TRPV2_Mmus    LLLGQLWYFW RRRISFMDSY FEILFLVQAL LTVLSQVLRF VETEWYLPLL VSSLVLGWLN LLYYTRGFQH TGIYSVMIQK
TRPV3_Hsap    ISVKEGIAIF LLRSILSDAW FHFVFFIQAV LVILSVFLYL FAYKEYLACL VLAMALGWAN MLYYTRGFQS MGMYSVMIQK
TRPV3_Mmus    ISVKEGIAIF LLRSILSDAW FHFVFFVQAV LVILSVFLYL FAYKEYLACL VLAMALGWAN MLYYTRGFQS MGMYSVMIQK
TRPV4_Hsap    FFFTNIKDLF MKKSLFIDGS FQLLYFIYSV LVIVSAALYL AGIEAYLAVM VFALVLGWMN ALYFTRGLKL TGTYSIMIQK
TRPV4_Clup    FFFTNIKDLF MKKSLFIDGS FQLLYFIYSV LVIVSAALYL AGIEAYLAVM VFALVLGWMN ALYFTRGLKL TGTYSIMIQK
TRPV4_Mmus    FFFTSIKDLF TKKSLFVDGS FQLLYFIYSV LVVVSAALYL AGIEAYLAVM VFALVLGWMN ALYFTRGLKL TGTYSIMIQK
TRPV4_Drer    FFVTNIKDLF LKKSIFVDGS FQLLYFIYSV LVVGSAALYL SGIEAYVSVM VFALTLGGMN PLYFTRGLKL TGTYSIMIQK
TRPV5_Hsap    ILLLEIPDIF RVGKTILGGP FHVIIITYAS LVLVTMVMRL TNTNGEVVPM SFALVLGWCS VMYFTRGFQM LGPFTIMIQK
TRPV5_Mmus    ILLLEIPDIF RVGQTVLGGP FHVIIITYAS LVLLTMAMRL TNVNGEVVPM SMALVLGWCS VMYFARGFQM LGPFTIMIQK
TRPV6_Hsap    ILLVEVPDIF RMGQTILGGP FHVLIITYAF MVLVTMVMRL ISASGEVVPM SFALVLGWCN VMYFARGFQM LGPFTIMIQK
TRPV6_Mmus    ILLVEIPDIF RLGQTILGGP FHVIIITYAF MVLVTMVMRL TNVDGEVVPM SFALVLGWCN VMYFARGFQM LGPFTIMIQK
```

```
TRPV6_Drer     ILLIEIPGIL AVGQTALGGL FHVTLISYAL LVLLLCGLRV TGIQGELIPM AFSLILGWFS LVYFARGFEM LGPYVIVIQK
TRPML1_Hsa     MWRQRGRVIS LWERLEFVNG WYILLVTSDV LTISGTIMKI GIYDVCSILL GTSTLLVWVG VIRYLTFFHN YNILIATLRV
TRPML1_Mmu     MWRRRGREIS LWERLEFVNG WYILLVTSDV LTISGTVMKI GIYDVCSILL GTSTLLVWVG VIRYLTFFHK YNILIATLRV
TRPML1_Dre     FRVSLGRSVS LGDRLEFING WYLLLIISDV LTIIASFIKI AIYDVCSILM GTSTLLVWVG VIRYFSFFQK YNILIVTLRA
TRPML2_Hsa     FLEKYKRPVC DTDQWEFING WYVLVIISDL MTIIGSILKM EIYDLCSIFL GTSTLLVWVG VIRYLGYFQA YNVLILTMQA
TRPML2_Mmu     FLEKYKQRVC GADQWEFVNG WYVLVTISDL MTIIGSILKM EIYDVCSILL GTSTLFVWVG VIRYLGYFQT YNVLILTMQA
TRPML2_Dre     CLDNYNHKVC EEDQREFLNG WYVLVIISDV MAIIGSILKM EIYDVCSILL GTSTLMVWVG VIRYLGYFEK YNVLILTMRA
TRPML3_Hsa     FLLHYKKEVS VSDQMEFVNG WYIMIIISDI LTIIGSILKM EIYDVCSILL GTSTMLVWLG VIRYLGFFAK YNLLILTLQA
TRPML3_Mmu     FLLHYKKEVS ASDQMEFING WYIMIIISDI LTIVGSVLKM EIYDVCSILL GTSTMLVWLG VIRYLGFFAK YNLLILTLQA
TRPML3_Dre     SLRRYRKCVS LSERLEFING WYLLIIVSDV LTISGSVLKI VIYDLCSILL GTGTMLVWIG VLRYMGXXCV VQILIITLRV
TRPML3_Dme     FRSQFGKELS FDGRLEFVNF WYIMIIFNDV LLIIGSALKE QIWDTCSLFL GIGNLLVWFG VLRYLGFFKT YNVVILTLKK
TRPML3_Dme     FRSQFGKELS FDGRLEFVNF WYIMIIFNDV LLIIGSALKE QIWDTCSLFL GIGNLLVWFG VLRYLGFFKT YNVVILTLKK
TRPML3_Cel     FENVLKNKIT VTDQLDFLNL WYVMIVVNDA LIIIGTVAKI SIFTLTSIFL GMGALLVYVG VLRYFGFFSQ YNILMLTLKR
TRPP_PKD1_     FAVAEARTWH REGRWRVLRL GAWARWLLVA LTAATALVRL AQSSAARGLA ASLLFLLLVK AAQQLRFVRQ WSVFGKTLCR
TRPP_PKD1_     FSMAEVQTWR KDGCACTARP DTWARCLLVI LTAATGLVRL AQGSVARGLA ASLLFLLLVK AAQQLRFVRQ WSVFGKTLCR
TRPP_PKD1_     FSVREGLLVW KQGRCYFLRV WNLASVCSLL LAVCVASLHL SRNQLLTQMS ATLLFLLVLK ASHQLRFLRE WGVFGRTLRK
TRPP_PKD2_     YVVEEILEIR IHKLHYFRSF WNCLDVVIVV LSVVAIGINI YRQIQFNNIA AVTVFFVWIK LFKFINFNRT MSQLSTTMSR
TRPP_PKD2_     YVVEEILEIR IHRLSYFRSF WNCLDVVIVV LSVVAMVINI YRQIQFNNIS AVMVFLVWIK LFKFINFNRT MSQLSTTMSR
TRPP_PKD2_     YLVEEALEIR LHRLRYFKSL WNCLDVLIVA LSVPAIIMNI CRQVHFNNLA AIIVFLSWVK LFKFINFNKT MNQLSTTMSR
TRPP_PKD2_     FIFEELFAIG RHRLHYLTQF WNLVDVVLLG FSVATIILSV NRENSYLNIK ACVVFVAWVK VFKFISVNKT MSQLSSTLTR
TRPP_PKD2L     YVVEEILELH IHRLRYLSSI WNILDLVVIL LSIVAVGFHI FRQTQYNNMN AVNLFFAWIK IFKYISFNKT MTQLSSTLAR
TRPP_PKD2L     YMVEEILELH IHRLHYLSSI WNILDLVVIM LSIVAVGFHI FRQTQYNNMN AVNLFFAWIK IFKYISFNKT MTQLSSTLAR
TRPP_PKD2L     YVVEEILEIH LHRLRYLSSV WNILDLVVIL LSIVAVGFHI FRQTQYNNMN AVNLFFAWIK IFKYISFNKT MTQLSSTLAR
TRPP_PKD2L     YIVEEILEFR IHKLSHFTSI WNILDIVVIL LAVVAIVFSA FRQTQYNNMN AVNLFFAWIK IFKYISFNKT MTQLTSTLAR
TRPP_PKD2L     FTTQEVKKIK EFKSAYFKSI WNWLELLLLL LCFVAVSFNT YYHIYYNNII AITIFFAWIK IFKFISFNKT MSQLSSTLSR
TRPP_PKD2L     FIIQELRKVN EFKSAYFRSV WNWLEMLLLL LCFLAVSFYA YCHIYYNNVI AITIFFAWIK IFKFISFNET MSQLSSTLSR
TRPP_PKD2L     YTIYEITEIR KSGKIYFCSM LNILDCAILL GCYLALVYNI WHNIIYVDMM AILAFLVWIK IFKFISFNKT LVQFTTTLKR
Cca7050        GHKTNCIIIT MFIIFYIVRL IVLRLTSYLL AVASTMSCIR LLQYLRVHRV IGPIQKSYGK VVQNMACFVV ILFIYLMAFA
Cca21784       DYKMDISMLV LFVLFFILRA IALRGSCYFL GPATVLACVR VLRYLSWHPV IGPVQRAISK IVEEILLFLV ILMVFLTAFA
Cca260897_     KYFKNVWNCL DILMILIAIV CMAFNIYRQV QFNHMSAVLI FFAWFKLFKY ISFNKTMNQL SSTLSKCAKD VLGFCVMFFI
Sla36613_6     ---------- ---------- -----YCYLL AFVVFMSTLK FINMLKFNRR MGLLTETIKA SVADMKGFAI TFFIYVMAFA
Sycon24227     VTDGNNYLQV GSILLILAIV PLRWLDQDAQ WHVACFAYIF VCLRILQLIS VTRMVGIYLQ ILTRILKDIS RFVIIFGIFL
Sycon24228     DGNNYLQVGS ILLILAIVPL RWLDQDAQWH VACFAYIFVC LRILQLISVT RMVGIYLQIL TRILKDISRF VIIFGIFLLT
Sycon9763_     ---------- ---------- ---------- ---------- ---------- ---------- ---------- --------SF
Ava16635_4     WNQFDTAFAL FFIMGTVLRL IPLSYSRHMY AVIGFVLFFR LLQFLVIIKD SGPFVYMVFR MLKNLGSFLI LALIFLLAYG
Ava9904_1      WNILDLALIL LFLLAFSIKL GCSAWVHRVY ALIILILYIR IFQYLIMSAY FGVIVLIIFA LAEEVVYFVF VLLLSMIGFG
Ava7536_5      WNYIDWLAYI LMVATIVSHV ADADWHARIG VVNIIVLWFR FLKYLRPFEF IGWFITIVIY LKADIIRFAI FLMTLLIPYS
Ava6620_1      WNILDIAIAI GFVATISLRI SVNVIARLVF GLFGVFLYMR FFQYVLVIRG LGPLIYTCYI AFKRMLKFLV IFVLVSLGFG
Cca31351       ---------- ---------- ---------Y TFMIPTGWIH AVYFLHSFNI EGQLIVSEIE AKKVPLKFRY PFFVEMLWHV
Cca30979_3     WNWVEMLVIV LGWTTTGVYI ARDTIYLTGS SLVVFFSFLK ILRLLQFNRR MLLLAGTLRR SGMQLIAFAI AFLIVMVAFA
Ava26193_2     YNYLDMGAIV CSLLIIPLRF SQENAQWFFA AIAYLLNVMR GYKFAVVLRT TGAYVEIIGS ILYDIVPFSI VFIMFLFGFS
Ava26160_2     ---------- ---------- ADADWHARIG VVNIIVLWFR FLKYLRPFEF IGWFITIVIY LKADIIRFAI FLMTLLIPYS

TRPC1_Hsap     MLQDFGKFLG MFLLVLFSFT IGLTQLY--- DTFHSFIGTC FALFWYIFSL VAIFVTRLQS FVGAVIVGTY NVVVVIVLTK
TRPC1_Mmus     QDFGKFLGMF LLVLFSFTIG LTQLY---DT FHSFIGTCFA LFWYIFSLVA IFVTRLQSFV GAVIVGTYNV VVVIVLTKL
TRPC1_Drer     MLQEFGKFLG LFLLVLISFT IGLTQLYGKD DTFHTFMGTC YALFWYIFSL VNLFVTRLRS FVGALIVGTY NIVVVIVLTK
TRPC2_Mmus     MIDDMIRFMF ILMIILTAFL CGLNNIYVPY EKLGNFNETF QFLFWTMFGM EEHTVVDVPE FVGRAMYGIF TIVMVIVLLN
TRPC2_Drer     MIDDMMRFMF ILVIIGTAFL CGINNIYVPY INLGKLNETF SFLFWTMFGM ADQTYVDLAE LVGRILYGIY TLIIVIVLLN
TRPC2_Drer     MIDDMMRFMF ILMIIGTAFL CGINNIYVPY IHLGRLNETF NFLFWTMFGM ANQGYVDLAE FVGRIFYGIF TLLIVIVLLN
TRPC3_Hsap     TVKDIFKFMV LFIMVFFAFM IGMFILYSYY GAFTTVEESF KTLFWSIFGL SEVTSVVFIE NIGYVLYGIY NVTMVVVLLN
TRPC3_Mmus     KDIFKFMVLF IMVFLAFMIG MFILYSYYGA FTTVEESFKT LFWSIFGLSE VTSVVFIENI GYVLYGIYNV TMVVVLLNML
TRPC4_Hsap     MLLDILKFLF IYCLVLLAFA NGLNQLYFYY EAFSTLFETL QSLFWSIFGL INLYVTNFTE FVGATMFGTY NVISLVVLLN
TRPC4_Mmus     MLLDILKFLF IYCLVLLAFA NGLNQLYFYY EAFSTLFETL QSLFWSIFGL INLYVTNFTE FVGATMFGTY NVISLVVLLN
TRPC4_Drer     MLLDILKFLF IYCLVLLAFA NGLNQLYFYY NAFSTLFETL QSLFWSIFGL ISLYVTNFTE FVGATMFGTY NVISLVVLLN
TRPC4_Dmel     MVMDIMKFFF LYVLVLFAFG SGLNQLLWYY DRFSNLFETT QTLFWAVFGL IDLDSFEFTR FWGMLMFGTY SVINIVVLLN
TRPC4_Cele     MVIDIVKFFM VYALVLFAFA CGLNQLLWYY SSCSSIYHTA ETLFWALFGL VDLTHFRLSE WTGKTIFGSY CCCSIIVLLN
TRPC5_Hsap     LDILKFLFIY CLVLLAFANG LNQLYFYYTA FSTLFETLQS LFWSVFGLLN LYVTNFTEFV GATMFGTYNV ISLVVLLNML
TRPC5_Mmus     LDILKFLFIY CLVLLAFANG LNQLYFYYTA FSTLFETLQS LFWSVFGLLN LYVTNFTEFV GATMFGTYNV ISLVVLLNML
TRPC6_Hsap     KDIFKFMVIF IMVFVAFMIG MFNLYSYYGA FTTVEESFKT LFWAIFGLSE VKSVVFIENI GYVLYGVYNV TMVIVLLNML
TRPC6_Mmus     KDIFKFMVIF IMVFVAFMIG MFNLYSYYGA FTTVEESFKT LFWAIFGLSE VKSVVFIENI GYVLYGVYNV TMVIVLLNML
TRPC6_Drer     TVKDIFKFMV IFILVFLAFM IGMFNLYSYY GAFTTVEESF KTLFWAIFGL SEVKSVVFIE NIGYVLYGVY NVTVVIVLLN
TRPC6_Drer     TVKDIFKFMV IFIMVFVAFM IGMFNLYSYY GAFTTVEESF KTLFWAIFGL SEVKSVVFIE NIGYVLYGVY NVTMVIVLLN
TRPC6_Cele     MLVDVAKFCF IFVLIISSFS IGLAQLYWYY PVFSSIADSY LTLLWSLFSI TKPEDTDITQ WVGQGMFIMY HCTSIIVLLN
TRPC7_Hsap     TVKDIFKFMV IFIMVFVAFM IGMFNLYSYY GAFTTVEESF KTLFWSIFGL SEVISVVFIE NIGYVLYGVY NVTMVVVLLN
TRPC7_Mmus     KDIFKFMVIF IMVFVAFMIG MFNLYSYYGA FTTVEESFKT LFWSIFGLSE VISVVFIENI GYVLYGVYNV TMVVVLLNML
TRPC7_Drer     TVKDIFKFMV IFIMVFLAFM IGMFNLYSYY GAFTTVEESF KTLFWSIFGL SEVISVVFIE NIGYILYGVY NVTMVVVLLN
TRPN_Dmel_     LLKDLARFLA VLAIFVFGFS MHIVALNQSF NYFSDPINSF ELLFFAVFGQ TTTEQTQWVE YLFKIVFGIY MLVSVVVLIN
TRPN_Cele_     LMYDLARFLV ILMLFVAGFT LHVTSIFQPA QRLASPSQTL EMLFFSLFGL VEPDSMPFAK IILKLLFGIY MMVTLIVLIN
TRPN_Drer_     LIKDLCRFAV ILMLFHTAFT LSLTALCQPL PTVPGPLNMS VLLFFALFGL TEPDKIPATA VLAKMVFGVY LVVTFIVLIN
TRPM1_Hsap     MMIDMLYFVV IMLVVLMSFG VARQAILHPE ESWKLARNIF YMPYWMIYGE VFADQIDPGA WLTPALMACY LLVANILLVN
TRPM1_Mmus     MMIDMLYFVV IMLVVLMSFG VARQAILHPE ESWKLARNIF YMPYWMIYGE VFADQIDPGA WLTPALMACY LLVANILLVN
TRPM2_Hsap     MMKDVFFFLF LLAVWVVSFG VAKQAILIHN EVDWLFRGAV YHSYLTIFGQ IP-GYIDFPE WLTVLLLCLY LLFTNILLLN
TRPM2_Mmus     MMKDVFFFLF LLAVWVVSFG VAKQAILIHN EVDWIFRGVV YHSYLTIFGQ IP-TYIDFPE WLTVTLLCLY LLFANILLLN
TRPM3_Hsap     MMIDMMYFVI IMLVVLMSFG VARQAILFPN ESWKLAKNIF YMPYWMIYGE VFADQIDTGA WIVPAIMACY LLVANILLVN
TRPM3_Mmus     MMIDMMYFVI IMLVVLMSFG VARQAILFPN ESWKLAKNIF YMPYWMIYGE VFADQIDTGA WIVPAIMACY LLVANILLVN
TRPM3_Drer     MMIDMMYFVI IMLVVLMSFG VARQAILNPN ESWMLARNIF FMPYWMIYGE VFADQIDTGA WIVPAIMACY LLVANILLVN
TRPM3_Dmel     MVKNMIYFVV LLAVVLMSFG VSRQAILYPN KTWSLIKEVT FQPYFMLYGE VFAGDIDTGH WVTPITMSMY LLIANILLIN
```

```
TRPM3_Cele   MIQNMSYIIV MLVVTLLSFG LARQSITYPD THWILVRNIF LKPYFMLYGE VYADEIDPGY WIPPLLMTFF LLIANILLMS
TRPM3_Cele   PTMIPLCVLV FITLYAFGLL RQSITYPYDH WILVRNIFLQ PYFMLYGEVY AAEIDPGYWI APVGLTVFML ATNVLLMNVM
TRPM3_Cele   WSMCYICVLL LVPLMAFGVN RQALTEPNKH WLLVRNIFYK PYFMLYGEVY AGEIDPGYFI PPLLMVIFLL VANILLLNIL
TRPM4_Hsap   MMKDVFFFLF FLGVWLVAYG VATEGLLRPR DFPSILRRVF YRPYLQIFGQ IPQEDMDYAN WLVVLLLVIF LLVANILLVN
TRPM4_Mmus   MMKDVFFFLF FLCVWLVAYG VATEGILRPQ DLPSILRRVF YRPYLQIFGQ IPQEEMDYAN WLVVLLLIVF LLVANILLLN
TRPM4_Drer   MIKDAFFFLF FLLVWLIAYG VANQALLYQY DPARVFRRVL YRPYLHIFGQ IPVEEIDSHN WLVVVLLVIF LLVTNILLIN
TRPM4_Drer   MIKDAFFFLF FLLVWLSAYG VANQALLYQY DPARTFRRVL YRPYLHIFGQ IPVEEIDSHN WLVVVLLVIF LLVTNILLVN
TRPM4_Drer   MIKHAFFFLF FLLVWLIAYG VANQALLYQY DPARVFRRVL YRPYLHIFGD IPVEEIDSHN WLVVILLVIF LLVTNILLIN
TRPM4_Drer   MVKDVFFFLF FLGVWLMAYG VANQALLYSY DPGWVARRVF YRPYMHIYGQ IPLDEIDDAN WLVLILLSVY LLVTNILLVN
TRPM5_Hsap   MMKDVFFFLF FLSVWLVAYG VTTQALLHPH DLEWIFRRVL YRPYLQIFGQ IPLDEIDYAN WLVILLLVTF LLVTNVLLMN
TRPM5_Mmus   MMKDVFFFLF FLSVWLVAYG VTTQALLHPH DLEWIFRRVL YRPYLQIFGQ IPLDEIDYAN WLVILLLVTF LLVTNVLLMN
TRPM5_Drer   MIKDVFFFLF FLSVWLIAYG VTTQALLHPN DIDWVFRRAL YRPYLHIFGQ IPLEEIDYAN WLVILLLVIY LLVTNVLLLN
TRPM6_Hsap   MTANMFYIVI IMAIVLLSFG VARKAILSPK ESWSLARDIV FEPYWMIYGE VYAGEIDPGS FLTPFLQAVY LFVQYIIMVN
TRPM6_Mmus   MAANMFYIVI IMAIVLLSFG VARKAILSPK ESWRLARDIV FEPYWMMYGE VYASDIDPGS FLTPFLQAVY LFVQYIIMVN
TRPM7_Hsap   MVANMFYIVV IMALVLLSFG VPRKAILYPH ESWTLAKDIV FHPYWMIFGE VYAYEIDPGT WLTPFLQAVY LFVQYIIMVN
TRPM7_Mmus   MVANMFYIVV IMALVLLSFG VPRKAILYPH ESWSLAKDIV FHPYWMIFGE VYAYEIDPGT WLTPFLQAVY LFVQYIIMVN
TRPM7_Drer   MVANMFYIVV IMAVVLLSFG VPRKAILYPE ESWTLAKDVV FQPYWMMYGE VYAYEIDTGV WLTPLLQAVY LFVQYILMVN
TRPM8_Hsap   MLIDVFFFLF LFAVWMVAFG VARQGILRQN EWRWIFRSVI YEPYLAMFGQ VP-SDVDFPE WITIPLVCIY MLSTNILLVN
TRPM8_Mmus   MLIDVFFFLF LFAVWMVAFG VARQGILRQN EWRWIFRSVI YEPYLAMFGQ VP-SDVDFPE WITIPLVCIY MLSTNILLVN
TRPA_Hsap_   LKTLLRSTVV FIFLLLAFGL SFYILLN--- PFSSPLLSII QTFSMMLGDI NYRESFAHPV LSFAQLVSFT IFVPIVLMNL
TRPA_Mmus_   FKTLLRSTGV FIFLLLAFGL SFYVLLN--- AFSTPLLSLI QTFSMMLGDI NYRDAFAYPV LTFGQLIAFT MFVPIVLMNL
TRPA_Drer_   ISRTLLSIIV LFFYLILGFA LSFYALMI-- -HFGRMFLSL LQTFVMMVGE MNYQDNFPFP DLTLAIFVWF VLLVPILLMN
TRPA_Drer_   IVRTLVCIMV LFVFLLLAFG LAFYALML-- -EFSSISLAL AQTFVMTVGE LNYQSTFAFP AITYFVFVFF VLLMPILLMN
TRPA_Dmel_   ILQTLIKVLM VFSILIIAFG LAFYILLSKI DSFSNIPMSL LRTFSMMLGE LDFVGTYKVP MTSFLILSVF MILMPILLMN
TRPA_Cele_   KTFFRFFPVF VLFIIAFSSS FYVILQ---E FSTIFMSPLK TTVMMIGEFE FTGIFAHTAV ACALFFFFCI IMTILLMNLL
TRPV1_Hsap   MIRDLCRFMF VYIVFLFGFS TAVVTLIEDG NSYNSLYSTC LELFKFTIGM GDLEFTEDFK AVFIILLLAY VILTYILLLN
TRPV1_Mmus   MIRDLCRFMF VYLVFLFGFS TAVVTLIEDG NSYNSLYSTC LELFKFTIGM GDLEFTEDFK AVFIILLLAY VILTYILLLN
TRPV1_Drer   MVGEIRRFLV VYMVFLIGFS AALVTLLDQE ISYKNIYFTT LELFKFTIGM GDLEFTDKYK EVFYVLLIVY IVMTYILMLN
TRPV2_Hsap   VIRDLLRFLL IYLVFLFGFA VALVSLSQEA RQYRGILEAS LELFKFTIGM GELAFQEHFR GMVLLLLLAY VLLTYILLLN
TRPV2_Mmus   VIRDLLRFLL VYLVFLFGFA VALVSLSREA SPYGGILDAS LELFKFTIGM GELAFQERFR GVVLLLLLAY VLLTYVLLLN
TRPV3_Hsap   VIHDVLKFLF VYIVFLLGFG VALASLIEKC KSYGSFSDAV LELFKLTIGL GDLNIQQKYP ILFLFLLITY VILTFVLLLN
TRPV3_Mmus   VIHDVLKFLF VYILFLLGFG VALASLIEKC KSYGSFSDAV LELFKLTIGL GDLNIQQTYP ILFLFLLITY VILTFVLLLN
TRPV4_Hsap   ILKDLFRFLL VYLLFMIGYA SALVSLLNPC NDSETFSTFL LDLFKLTIGM GDLEMLSKYP VVFIILLVTY IILTFVLLLN
TRPV4_Clup   ILKDLFRFLL VYLLFMIGYA SALVSLLNPC NDSETFSTFL LDLFKLTIGM GDLEMLSKYP VVFIILLVTY IILTFVLLLN
TRPV4_Mmus   ILKDLFRFLL VYLLFMIGYA SALVTLLNPC NDSETFSAFL LDLFKLTIGM GDLEMLSKYP VVFILLLVTY IILTFVLLLN
TRPV4_Drer   ILKDLFRFLL VYVLFMIGYA SALVSLLTIC NDTNTFSEFL LDLFKLTIGI GDLDMLKQYP AVFLILLVTY IILTFVPLLN
TRPV5_Hsap   MIGDLMRFCW LMAVVILGFA SAFYIIFQTE PQFYDYPMAL FTTFELFLTV IDAPNYDDLP FMFSIVNFAF AIIATLLMLN
TRPV5_Mmus   MIGDLLRFCW LMAMVILGFA SAFYIIFQTE PEFSDYPTAM FSTFELFLTI IDGPNYRDLP FMYSVTYATF AIIATLLMLN
TRPV6_Hsap   MIGDLMRFCW LMAVVILGFA SAFYIIFQTE PHFYDYPMAL FSTFELFLTI IDGPNYNDLP FMYSITYAAF AIIATLLMLN
TRPV6_Mmus   MIGDLMRFCW LMAVVILGFA SAFYIIFQTE PHFYDYPMAL FSTFELFLTI IDGPNYDDLP FMYSVTYAAF AIIATLLMLN
TRPV6_Drer   SIGDITKFMW LSIIFLIGSS AALWIFYMTQ PQYRSFPITL FSQFEVSVGQ IDLPDHTFTH PVVYWTHVCF SLISNVLLFN
TRPML1_Hsa   ALPSVMRFCC CVAVIYLGYC FCGWIVLGPY VKFRSLSMVS ECLFSLINGD DMFVTFALVW LFSQLYLYSF ISLFIYMVLS
TRPML1_Mmu   ALPSVMRFCC CVAVIYLGYC FCGWIVLGPY VKFRSLSMVS ECLFSLINGD DMFVTFALVW LFSQLYLYSF ISLFIYMVLS
TRPML1_Dre   AFPNVIRFCC CAAAIYMGYC FCGWIVLGPY AKFRSLSTVS ECLFSLINGD DMFATFSLVW VFSQIYLYTF ISLFIYMVLS
TRPML2_Hsa   SLPKVLRFCA CAGMIYLGYT FCGWIVLGPY DKFENLNTVA ECLFSLVNGD DMFATFALVW LFSRLYLYSF ISLFIYMILS
TRPML2_Mmu   SLPKVLRFCA CAGMIYLGYT FCGWIVLGPY EKFENLNIVA ECLFSLVNGD DMFATFALVW LFSRLYLYSF ISLFIYMVLS
TRPML2_Dre   ALPKVLRFCC CAGMIYLGYT FCGWIVLGPY EKFEDLSRVA ECLFSLVNGD DMFATFAMVW LFSRVYLYSF ISLFIYMVLS
TRPML3_Hsa   ALPNVIRFCC CAAMIYLGYC FCGWIVLGPY DKFRSLNMVS ECLFSLINGD DMFATFALVW LFSRIYLYSF ISLFIYMILS
TRPML3_Mmu   ALPNVMRFCC CAAMIYLGYC FCGWIVLGPY EKFRSLNRVS ECLFSLINGD DMFSTFALVW LFSRVYLYSF ISLFIYMILS
TRPML3_Dre   ALPNVIRFTC CAAMIYLGYC FCGWIVLGPY TKFRTLNTVS ESLFSLINGD DMFATFKVVW LFSRLYLYTF VSLFIYMVLS
TRPML3_Dme   AAPKILRFLI AALLIYAGFV FCGWLILGPY MKFRSLATTS ECLFALINGD DMFATFAWLW WFCQIYLYSF ISLYIYVVLS
TRPML3_Dme   AAPKILRFLI AALLIYAGFV FCGWLILGPY MKFRSLATTS ECLFALINGD DMFATFAWLW WFCQIYLYSF ISLYIYVVLS
TRPML3_Cel   SAPNIMRFMT CAIVLYAGFL IAGWVIIGPY MKFRTLAESS EALFSLLNGD DMFATFYVIK VFGTVYIYLF VSLFIYVVLS
TRPP_PKD1_   ALPELLGVTL GLVVLGVAYA QLAILLVSSC D----SLWSV AQAL--LVLC -------LSP LLC----VGL WALRLWGALR
TRPP_PKD1_   ALPELMGATL GLVLLGVAYA QMAILLISSG D----TLYNM ARAF--LVLC -------LSP LLC----VGL WALRVWGALR
TRPP_PKD1_   SFWELLMVAV TLLVFLLAYS HTGHLLFHSV E----GYGTV SSTCFKLIGS -------PCS GSCFIFYISF TLLRLVLLWI
TRPP_PKD2_   CAKDLFGFAI MFFIIFLAYA QLAYLVFGTQ DDFSTFQECI FTQFRIILGD INFAEIEANR VLGPIYFTTF VFFMFFILLN
TRPP_PKD2_   CAKDLFGFTI MFSIIFLAYA QLAYLVFGTQ DDFSTFQECI FTQFRIILGD INFAEIEANR VLGPLYFTTF VFFMFFILLN
TRPP_PKD2_   CAKDLMGFAI MFFIVFLAYA QLAYLVFGTQ NDFSTFQACI FTQFRIILGD FDFSEIEADS VLGPIYFTTF VFFIFMILLN
TRPP_PKD2_   SAKDIGGFAV MFAVFFFAFA QFGYLCFGTQ ADYSNLYNSA FALLRLILGD FNFSALECNR FFGPAFFIAY VFFVSFILLN
TRPP_PKD2L   CAKDILGFAV MFFIVFFAYA QLGYLLFGTQ ENFSTFIKCI FTQFRIILGD FDYNAIDANR ILGPAYFVTY VFFVFFVLLN
TRPP_PKD2L   CAKDILGFAV MFFIVFFAYA QLGYLLFGTQ ENFSTFIKCI FTQFRIILGD FDYNAIDANR ILGPAYFVTY VFFVFFVLLN
TRPP_PKD2L   CAKDILGFAI MFFIVFFAYA QLGYLLFGTQ ENFSTFVKCI FTQFRIILGD FDYNAIDANR ILGPVYFVTY VFFVFFVLLN
TRPP_PKD2L   CALDIFGFAI MFFIVFFAYA QLGYLLFGTE ETFSTFNKCI FTQFRIILGD FDYDAIDANR VLGPIYFFSY VFFVFFVLLN
TRPP_PKD2L   CVKDIVGFAI MFFIIFFAYA QLGFLVFGSQ DDFSTFQNSI FAQFRIVLGD FNFAGIQANP ILGPIYFITF IFFVFFVLLN
TRPP_PKD2L   CMKDIVGFAI MFFIIFSAYA QLGFLVFGSQ DDFSTFQNSI FAQFRIVLGD FNFAGIQANW ILGPIYFITF IFFVFFVLLN
TRPP_PKD2L   CSKDLAGFSL MFGIVFLAYA QLGLLLFGTK PDFRNFITSI LTMIRMILGD FQYNLIEANR VLGPIYFLTY ILLVFFILLN
Cca7050      SGLVNIYGAT FGFESMQSSA QSLFWSLFGL LDVGDLSEEA TAGKIVFGLW LLQAMIILLN MLIALVTNKF DEFQSNADTE
Cca21784     AGITNVYRGT FDFSGVWISG ASLFWALFGL LERQDMEAEE TAGQITMAVW LVSSVVILLN MLIAVVTNKF DEVEENADTE
Cca260897_   VFFAFAQLGY LVFGAQRDFS TFGDSVFTLF RIILGDFDFI ALEANRILGP IFFVTYVFFV FFVLLNMFLA IINDTYGEVK
Sla36613_6   MFAFLLFGTQ AAFGSFGSTI TSLFLFVLGT YDFESMQAQP FLGPVFFFLF IFIVFFGLQA MFLTIIIEAF ETVRTDVKQK
Sycon24227   LTYAGSLFLA LRGEVELGLN HFTSNYGFIL VTGLMIQQSI VSFSWLGIVI NMLFLFFVLV VLLNILIAQL SDTYADVKAD
Sycon24228   YAGSLFLALR GEVELGLNHF TSNYGFILVT GLMIQQSIVS FSWLGIVINM LFLFFVLVVL LNILIAQLSD TYADVKADAQ
Sycon9763_   SGVTHAVENE APEFFSYADT FVYLFRLAIG ITEFSSTYPV PGWAVAFFCI YVVLGFLLLI QLLIAMMNAT HQRVEKDREA
Ava16635_4   VASQTILYPN PYAFVFGNIF FRPYFQVFGE FFLEHIQSSN FFVYFFLVIW LILSNILLIN IIIAKFNNTF VEIESNAAIY
Ava9904_1    AAMQAVVASD LAQFLLFTIF FRPYWQLFGE FFLPQLGTRH PLPYFILALW LIFSNVLLVN LLIAKFNNIY LIVEAKSSIL
Ava7536_5    IGVWVIYQFQ RDFNTFYSTV FTVFRIIVID -------TDE WMTYFIIGSY ISIVSIVSLN LFIALLSNTF QTVYDNSKAT
```

```
Ava6620_1    IIEVTLTSTN NGWNLLRILL FSPYYQIFGE FGLANIRSTT FIAYVFLIIL QLIANVLLLN LMIAYFTKIF NEISENADSI
Cca31351     LSQYA----- -----EHVEM FGLLSDNDGW DELSAIDAVH PTGINQLSMY EIRGLLSLVE LLVKMLLRGY NIQYNYMSTV
Cca30979_3   SLHMGVFGAS YRFSSMFSAL QYQIEMILGK FEANRSRDYP IAAPLIFIMF VLTGAMLLSN LFIVLINETF SAVKRDNQTQ
Ava26193_2   GATYLALKFE VASSLTYLVS EWWFVFFNGL VIESPVFGFG WFSLILHATF LFFVIVVFLN LIIAQMSDTY QNVWSDAQRK
Ava26160_2   IGVWVIYQFQ RDFNTFYSTV FTVFRIIVID -------TDE WMTYFIIGSY ISIVSIVSLN LFIALLSNTF QTVYDNSKAT

TRPC1_Hsap   LLVAMLHKSF QLIANHEDKE WKFARAKLWL SYFDDKLRNE IRDL
TRPC1_Mmus   VAMLHKSFQL IANHEDKEWK FARAKLWLSY FDDKLRNEIR DL
TRPC1_Drer   LLVAMLHKSF RQIANHEDKE WKFARAKLWL SYFDDKLRNE MRDL
TRPC2_Mmus   MLIAMITNSF QKIEDDADVE WKFARSKLYL SYFREGVQSE VASV
TRPC2_Drer   MLIAMITNSF QKIEDDADVE WKFARSKLYL SYFREGIHTE MKQI
TRPC2_Drer   MLIAMISNSF QRIEDDADVE WKFARSKLYL SYFREGVHTE MKDF
TRPC3_Hsap   MLIAMINSSY QEIEDDSDVE WKFARSKLWL SYFDDGIRYE LLED
TRPC3_Mmus   IAMINSSYQE IEDDSDVEWK FARSKLWLSY FDDGIRYELL ED
TRPC4_Hsap   MLIAMMNNSY QLIADHADIE WKFARTKLWM SYFEEGIRFE VLGL
TRPC4_Mmus   MLIAMMNNSY QLIADHADIE WKFARTKLWM SYFEEGIRFE VLGL
TRPC4_Drer   MLIAMMNNSY QHIADHADIE WKFARTKLWM SYFEEGIRYE VIGM
TRPC4_Dmel   LLIAMMNHSY QLISERADVE WKFARSKLWI SYFEEGIRCE LVEI
TRPC4_Cele   MLIAMMSNSY QYISDQADIE WKFARSRLFL EYFDDTL--- ----
TRPC5_Hsap   IAMMNNSYQL IADHADIEWK FARTKLWMSY FDEGIRYEVL DL
TRPC5_Mmus   IAMMNNSYQL IADHADIEWK FARTKLWMSY FDEGIRYEVL DL
TRPC6_Hsap   IAMINSSFQE IEDDADVEWK FARAKLWFSY FEEGIRYELL EE
TRPC6_Mmus   IAMINSSFQE IEDDADVEWK FARAKLWFSY FEEGIRYELL EE
TRPC6_Drer   MLIAMINNSF QEIEDDADVE WKFARAKLWF TYFEEGIRYE LLEE
TRPC6_Drer   MLIAMINNSF QEIEDDADVE WKFARAKLWF SYFEEGIRFE LLEE
TRPC6_Cele   MLIAMMSHSF QIINDHADLE WKFHRTKLWM AHFDEGIRYE LRDD
TRPC7_Hsap   MLIAMINNSY QEIEEDADVE WKFARAKLWL SYFDEGIRYE LLEE
TRPC7_Mmus   IAMINNSYQE IEEDADVEWK FARAKLWLSY FDEGIRYELL EE
TRPC7_Drer   MLIAMINHSY QEIEEDADVE WKFARAKLWL SYFDEGIRYE LLEE
TRPN_Dmel_   LLIAMMSDTY QRIQAQSDIE WKFGLSKLIR NMHRTTSSDA ESGS
TRPN_Cele_   LLIAMMSDTY QRIQAQSDKE WKFGRAILIR QMNKKSRDVD LAMA
TRPN_Drer_   LLIAMMSDTY QRIQAQSDTE WKFGRAVLIR DMSRKSTDE- ----
TRPM1_Hsap   LLIAVFNNTF FEVKSISNQV WKFQRYQLIM TFHDRPLSNR MVNA
TRPM1_Mmus   LLIAVFNNTF FEVKSISNQV WKFQRYQLIM TFHDRPLSGR MVSA
TRPM2_Hsap   LLIAMFNYTF QQVQEHTDQI WKFQRHDLIE EYHGRPTLHW IVRT
TRPM2_Mmus   LLIAMFNYTF QEVQEHTDQI WKFQRHDLIE EYHGRPVLHW IVTT
TRPM3_Hsap   LLIAVFNNTF FEVKSISNQV WKFQRYQLIM TFHERPLIGR MATA
TRPM3_Mmus   LLIAVFNNTF FEVKSISNQV WKFQRYQLIM TFHERPLIGR MATA
TRPM3_Drer   LLIAVFNNTF FEVKSISNQV WKFQRYQLIM TFHERPMIGR IAVA
TRPM3_Dmel   LLIAVFNNIF NEVNSVSHQV WMFQRFTVVM EYQQKPMSEQ ILSH
TRPM3_Cele   MLIAIFNHIF DATDEMSQQI WLFQRYKQVM EYESTPVQNE ILEC
TRPM3_Cele   VAGCTYIFEK HIQSTREIFL FERYGQVMEY ESTPMHKEQM NL
TRPM3_Cele   IAIFNNIYND SIEKSKEIWL FQRYQQLMEY HDSPISDEVV QI
TRPM4_Hsap   LLIAMFSYTF GKVQGNSDLY WKAQRYRLIR EFHSRPCLGW VAEA
TRPM4_Mmus   LLIAMFSYTF SKVHGNSDLY WKAQRYSLIR EFHSRPCLTW MAEA
TRPM4_Drer   LLIATFSYTF TKVQEHSDKY WKFQRYNLIV EYHSRPCLQW IMET
TRPM4_Drer   LLIATFSYTF SKVQERSDTY WKFQRYNLIV EYHSRPCLQW IMET
TRPM4_Drer   LLIATFSYTF TKVQEHSDKY WKFQRYNLIV EYHSRP---W IMET
TRPM4_Drer   LLIAMFSYTF NKVQERSDVY WKFQRYNLIV EYHSRPCLSW IVEA
TRPM5_Hsap   LLIAMFSYTF QVVQGNADMF WKFQRYNLIV EYHERPCVSS VADV
TRPM5_Mmus   LLIAMFSYTF QVVQGNADMF WKFQRYHLIV EYHGRPCLSS MTDT
TRPM5_Drer   LLIAMFSYTF QVVQENADIF WKFQRYNLIV EYHSRPCLSW IAEC
TRPM6_Hsap   LLIAFFNNVY LDMESISNNL WKYNRYRYIM TYHEKPLSAL TVDT
TRPM6_Mmus   LLIACFNNIY LDIKSISNKL WKYNRYRYIM TYHQKPLSAI TVDT
TRPM7_Hsap   LLIAFFNNVY LQVKAISNIV WKYQRYHFIM AYHEKPLSAL TVDT
TRPM7_Mmus   LLIAFFNNVY LQVKAISNIV WKYQRYHFIM AYHEKPLSAL TVDT
TRPM7_Drer   LLIAFFNNVY LQVKSISNLV WKYQRYHFIL AYHDKPLSAL TVDT
TRPM8_Hsap   LLVAMFGYTV GTVQENNDQV WKFQRYFLVQ EYCSRL---- ----
TRPM8_Mmus   LLVAMFGYTV GIVQENNDQV WKFQRYFLVQ EYCNRL---- ----
TRPA_Hsap_   LIGLAVGDIA EVQKHASLKR IAMQVELHTS LEKKLQIKLI IQK
TRPA_Mmus_   LIGLAVGDIA EVQKHASLKR IAMQVELHTN LEKKLQIKLI IQK
TRPA_Drer_   LLIGLAVGDI AEVQTNACLK RIAMQIELHT NLEERLQLKL IVQK
TRPA_Drer_   LMIGLAVGDI AEVQRNAELK RIAMQIDLHT ALEEKLQLKL IIQK
TRPA_Dmel_   LLIGLAVGDI ESVRRNAQLK RLAMQVVLHT ELERKLQVRL IVQK
TRPA_Cele_   VGLAVDDIKG VQEKAELKRL AMQVDLVLQI EASLQLKNIQ EN
TRPV1_Hsap   MLIALMGETV NKIAQESKNI WKLQRAITIL DTEKSFAEVY LRQF
TRPV1_Mmus   MLIALMGETV NKIAQESKNI WKLQRAITIL DTEKSFTEVQ LKHY
TRPV1_Drer   MLIALMNQSV EMMSVESTSI WKLQRAITTL DMEWILLSSS V---
TRPV2_Hsap   MLIALMSETV NSVATDSWSI WKLQKAISVL EMENGY---- ----
TRPV2_Mmus   MLIALMSETV NSVATDSWSI WKLQKAISVL EMENGY---- ----
TRPV3_Hsap   MLIALMGETV ENVSKESERI WRLQRARTIL EFEKML---- ----
TRPV3_Mmus   MLIALMGETV ENVSKESERI WRLQRARTIL EFEKML---- ----
TRPV4_Hsap   MLIALMGETV GQVSKESKHI WKLQWATTIL DIERSFEPLD SMGN
TRPV4_Clup   MLIALMGETV GQVSKESKHI WKLQWATTIL DIERSFEPLD NMGN
TRPV4_Mmus   MLIALMGETV GQVSKESKHI WKLQWATTIL DIERSFEPLD NLGN
TRPV4_Drer   MLIALMGETV GQVSKESKKI WKLQWATTIL DIERSFTEPL TGRH
TRPV5_Hsap   LFIAMMGDTH WRVAQERDEL WRAQVVATTV MLERKLGSEG DGEE
```

```
TRPV5_Mmus   LFIAMMGDTH WRVAQERDEL WRAQVVATTV MLERKMGGEG DGEE
TRPV6_Hsap   LLIAMMGDTH WRVAHERDEL WRAQIVATTV MLERKLGGES WEYQ
TRPV6_Mmus   LLIAMMGDTH WRVAHERDEL WRAQVVATTV MLERKLGGEG WEYQ
TRPV6_Drer   LLVAMMSDTQ WRVTQERDEL WRTQVVATTL MLERKL--EC EKEE
TRPML1_Hsa   LFIALITGAY DTIKHPGGAG AEESELQAYI A---Q-CPTS GKFR
TRPML1_Mmu   LFIALITGAY DTIKHPGGTG TEKSELQAYI E---Q-CPTS GKFR
TRPML1_Dre   LFIALITGAY DTITQQTQDV PQVSELHRFI A---E-CPTS GNFR
TRPML2_Hsa   LFIALITDSY DTIKKFQQNG FPETDLQEFL K---E-CSSK EEYQ
TRPML2_Mmu   LFIALITDSY HTIKKYQQHG FPETDLQKFL K---E-SGSK DGYQ
TRPML2_Dre   LFIALITDAY ETIKGYQTTG FPMTELHWFL KGQKE-CQGQ EEME
TRPML3_Hsa   LFIALITDTY ETIKQYQQDG FPETELRTFI S---E-CPNS GKYR
TRPML3_Mmu   LFIALITDTY ETIKHYQQDG FPETELRKFI A---E-CPNS GKYR
TRPML3_Dre   LFITLITDTY DTIKHQQLDG EPVSDLQAFV L---Q-CPDS GEFS
TRPML3_Dme   LFIAVIMDAY DTIKAYYKDG FPTTDLKAFV GTR---TISS GVFM
TRPML3_Dme   LFIAVIMDAY DTIKAYYKDG FPTTDLKAFV GTR---TISS GVFM
TRPML3_Cel   LFIAIIMDAY EVVKDRYSDG LRAIEKRGCL RDFVE-PSAY APSN
TRPP_PKD1_   LGAVILRWRY HALRGELRPA WEPQDYEMVE LFLRRLVEQQ LHSL
TRPP_PKD1_   LGAILLRWRY HALRGELRPA WEPQDYEMVE LFLRRLVEQQ LQSL
TRPP_PKD1_   LMSALLR-NY RRARAELRPA VDLQDYEMVE LFLRRLLEYR IERL
TRPP_PKD2_   MFLAIINDTY SEVKSDLQQK AEMELSDLIR KGYHKAIIDA VIVK
TRPP_PKD2_   MFLAIINDSY SEVKSDLQQK AEMELSDLIR KGCQKAIIDA VIVK
TRPP_PKD2_   MFLAIINDTY SEVKADMQQR SEMEITDLIK KSYNRAIIDA VIVK
TRPP_PKD2_   MFLAIINDSY VEVKAELRKK DGEGILDWFM NKVRGLIIEG VNAT
TRPP_PKD2L   MFLAIINDTY SEVKEELGQK DELQLSDLLK QGYNKTVIDA VGSK
TRPP_PKD2L   MFLAIINDTY SEVKEELGQK DELQLSDLLK QGYNKTVVDA VGSK
TRPP_PKD2L   MFLAIINDTY SEVKEELGQK DQLQLSDFLK QSYNKTVIDA VGSK
TRPP_PKD2L   MFLAIINDTY SEVKSELSQK DEFQIADLIK QSYAKTILDF VMEK
TRPP_PKD2L   MFLAIINDTY SEVKADYGRR LDFELGKMIK QSYKNV--EC LTKR
TRPP_PKD2L   MFLAIINDTY SEVKADYGRR PDFELGKIIQ KSCFNVVLNQ LMRK
TRPP_PKD2L   MFLAIIMETY NTVKGEI-TQ GRSHLGSYIY RKLSGMLMDD ILKR
Cca7050      WKASFGATVI DVQKSQ---- ----
Cca21784     WKFTRAELLM QVKHTH---- ----
Cca260897_   AEIRDDDEMT VTDYFKKSYN KL--------
Sla36613_6   NDYEVFDYIL GKFKNI---- ----
Sycon24227   AQRELEQNWA ASLRNLEQGT TRQSLDEQ
Sycon24228   RELEQNWAAS LRNLEQGTTR QSLDEQ
Sycon9763_   VWX------- ---------- -----
Ava16635_4   WKYKFFNSVK EFRDKPSRRI LIRY
Ava9904_1    WKFTRYSAIE EFRLKPVCHT LLDH
Ava7536_5    ATMEKARILL SFERKLLMGE LEKR
Ava6620_1    YLTQFLEVVD EYQRKSVGVK SVNL
Cca31351     WKEERLKTVS SDFLS-DCRF CLDM
Cca30979_3   NKYEVVEYIK ELVGL----- ----
Ava26193_2   LYKNRAWILA RIEHNSILQA RDKQ
Ava26160_2   ATMEKARILL SFERKL---- ----
```

Fig S4 phylobayes analysis of the data in Fig 5a

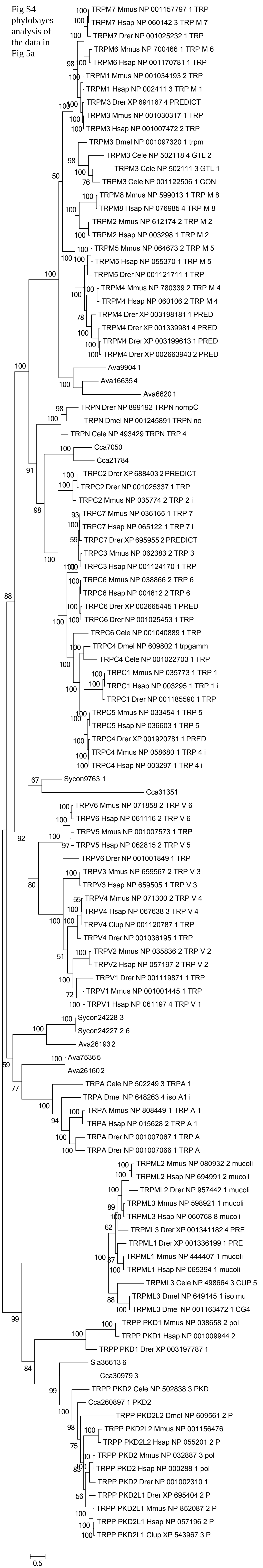

Supplementary figure S5: Full alignment for sequences shown in figure 5c

```
TRPC1_Hsap_NP_003295.1      ---------- ---------- ---------- ---------- ---------- ----------
TRPC1_Mmus_NP_035773.1      ---------- ---------- ---------- ---------- ---------- ----------
TRPC4 Hsap_NP_003297.1      ---------- ---------- ---------- ---------- ---------- ----------
TRPC4_Mmus_NP_058680.1      ---------- ---------- ---------- ---------- ---------- ----------
TRP_Spur XP_793901          ---------- ---------- ---------- ---------- ---------- ----------
TRP_Cint XP_002124651.2     ---------- ---------- ---------- ---------- ---------- ----------
TRP_Skow_XP_002733765.1     ---------- ---------- ---------- ---------- ---------- ----------
TRP_Lflor__emb|CAA11261.1   ---------- ---------- ---------- ---------- ---------- ----------
TRP_Bflo_XP_002611405.1     ---------- ---------- ---------- ---------- ---------- ----------
TRP4_Sman_XP_002576849.1    ---------- ---------- ---------- ---------- ---------- ----------
TRP_Nvec4_XP_001640409      ---------- ---------- ---------- ---------- ---------- ----------
TRP_Nvec1_XP_001637374.1    ---------- ---------- ---------- ---------- ---------- ----------
Cca7050                     ---------- ---------- ---------- ---------- ---------- ----------
Cca21784                    ---------- ---------- ---------- ---------- ---------- ----------
Ava16635_4                  VNPLHINRDR HGSLASNNIS LLKYTLMSEN SEQLSAGSAY FTASPRDNYT TYDMSKQASL
Ava9904_1                   ---------- ---------- ---------- ---------- -------NKT KYSFT-----
Ava6620_1                   ---------- ---------- ---------- ---------- ---------- ----------

TRPC1_Hsap_NP_003295.1      ---------- ---------- ---------- ---------- ---------- ----------
TRPC1_Mmus_NP_035773.1      ---------- ---------- ---------- ---------- ---------- ----------
TRPC4 Hsap_NP_003297.1      ---------- ---------- ---------- ---------- ---------- ----------
TRPC4_Mmus_NP_058680.1      ---------- ---------- ---------- ---------- ---------- ----------
TRP_Spur XP_793901          ---------- ---------- ---------- ---------- ---------- ----------
TRP_Cint XP_002124651.2     ---------- ---------- ---------- ---------- ---------- ----------
TRP_Skow_XP_002733765.1     ---------- ---------- ---------- ---------- ---------- ----------
TRP_Lflor__emb|CAA11261.1   ---------- ---------- ---------- ---------- ---------- ----------
TRP_Bflo_XP_002611405.1     ---------- ---------- ---------- ---------- MRRHNYDKEH IMIVTMIGGE
TRP4_Sman_XP_002576849.1    ---------- ---------- ---------- ---------- ---------- ----------
TRP_Nvec4_XP_001640409      ---------- ---------- ---------- ---------- ---------- ----------
TRP_Nvec1_XP_001637374.1    ---------- ---------- ---------- ---------- ---------- ----------
Cca7050                     ---------- ---------- ---------- ---------- ---------- ----------
Cca21784                    ---------- ---------- ---------- ---------- ---------- ----------
Ava16635_4                  DLQVMDNPTP SPTGSQTDRL ISSSLTPQTS ERNVTKSLNN LEASRLRPRL PFVKQAMGNK
Ava9904_1                   ---------- ---------- ---------- ---------- --------RL DFISRA----
Ava6620_1                   ---------- ---------- ---------- ---------- ---------- ----------

TRPC1_Hsap_NP_003295.1      ---------- ---------- -----MMAAL YPSTDLSGAS SSSLPSS--- ----------
TRPC1_Mmus_NP_035773.1      ---------M GAPPPSPGLP PSWAAMMAAL YPSTDLSGVS SSSLPSS--- ----------
TRPC4 Hsap_NP_003297.1      ---------- ---------- ---------- ---------- ---------- ----------
TRPC4_Mmus_NP_058680.1      ---------- ---------- ---------- ---------- ---------- ----------
TRP_Spur XP_793901          ---------- ---------- ---------- ---------- ---------- ----------
TRP_Cint XP_002124651.2     ---------- ---------- ---------- ---------- ---------- ----------
TRP_Skow_XP_002733765.1     ---------- ---------- ---------- ---------- ---------- -MMELSSSTL
TRP_Lflor__emb|CAA11261.1   ---------- ---------- ---------- ---------- ---------- ----------
TRP_Bflo_XP_002611405.1     CVSCTSLQSN GHGNKPTHGV PPIATNMAAT IPDEVLQEED ADRIPLRVVH HESDLSEAEK
TRP4_Sman_XP_002576849.1    ---------- ---------- ---------- ---------- ---------- ----------
TRP_Nvec4_XP_001640409      ---------- ---------- ---------- ---------- ---------- ----------
TRP_Nvec1_XP_001637374.1    ---------- ---------- ---------- ---------- ---------- ----------
Cca7050                     ---------- ---------- ---------- ---------- ---------- ----------
Cca21784                    ---------- ---------- ---------- ---------- ---------- ----------
Ava16635_4                  TFDFNSPNSG AVPFSGSLPR KRVMLQRIES HANSTLDEEF ARELKNSMDT MLQEEPGSPL
Ava9904_1                   ---------- ---------- ---------- ---------- ---------- ----------
Ava6620_1                   ---------- ---------- ---------- ---------- ---------- ----------

TRPC1_Hsap_NP_003295.1      ---------- ---------- ---------- ------PSSS SPNEVMALKD -----VREVK
TRPC1_Mmus_NP_035773.1      ---------- ---------- ---------- ------PSSS SPNEVMALKD -----VREVK
TRPC4 Hsap_NP_003297.1      ---------- ---------- ---------- ------MAQF YYKRNVNAPY RDRIPLRIVR
TRPC4_Mmus_NP_058680.1      ---------- ---------- ---------- ------MAQF YYKRNVNAPY RDRIPLRIVR
TRP_Spur XP_793901          ---------- ---------- ---------- ---------- ---------- ----------
TRP_Cint XP_002124651.2     ---------- ---------- ---------- ---------- ---------- ----------
TRP_Skow_XP_002733765.1     ELVTPPTRGD SPMMFPRNAN Y----QNRGV ASGLLNAAFT QRRSASVISG VSSIPLQLFS
TRP_Lflor__emb|CAA11261.1   ---------- ---------- ---------- ---------- ---------- ----------
TRP_Bflo_XP_002611405.1     RFLVSVERGD YATVRKILED YGPGEQSGRL FVCLFNMAAT IPDEVLQEED ADRIPLRVVH
TRP4_Sman_XP_002576849.1    ---------- ---------- ---------- ---------- ---------- ----------
TRP_Nvec4_XP_001640409      ---------- ---------- ---------- ---------- ---------- ----------
TRP_Nvec1_XP_001637374.1    ---------- ---------- ---------- ---------- ---------- ----------
Cca7050                     ---------- ---------- ---------- ---------- ---------- ----------
Cca21784                    ---------- ---------- ---------- ---------- ---------- ----------
Ava16635_4                  NNQEALNRAD TEVIKRQTIL MSRHENKENI TSVNENETQS LSSK-TKSSI FSKMKIDHAS
Ava9904_1                   ---------- ---------- --------TM TSCSLQEKDW PQIKPSRSRV FSRLSITSGN
Ava6620_1                   ---------- ---------- ---------- ---------- ---------- ----------

TRPC1_Hsap_NP_003295.1      E--------- ---------- ---------- ---------- ---------- ----------
TRPC1_Mmus_NP_035773.1      E--------- ---------- ---------- ---------- ---------- ----------
TRPC4 Hsap_NP_003297.1      A--------- ---------- ---------- ---------- ---------- ----------
TRPC4_Mmus_NP_058680.1      A--------- ---------- ---------- ---------- ---------- ----------
TRP_Spur XP_793901          ---------- ---------- ---------- ---------- ---------- ----------
```

```
TRP_Cint XP_002124651.2     ---------- ---------- ---------- ---------- ---------- ----------
TRP_Skow_XP_002733765.1     E--------- ---------- ---------- ---------- ---------- ----------
TRP_Lflor__emb|CAA11261.1   ---------- ---------- ---------- ---------- ---------- ----------
TRP_Bflo_XP_002611405.1     H--------- ---------- ---------- ---------- ---------- ----------
TRP4_Sman_XP_002576849.1    ---------- ---------- ---------- ---------- ---------- ----------
TRP_Nvec4_XP_001640409      ---------- ---------- ---------- ---------- ---------- ----------
TRP_Nvec1_XP_001637374.1    ---------- ---------- ---------- ---------- ---------- ----------
Cca7050                     ---------- ---MSTDCLD VDMLVMLCQS DVDLLLSDEN NITAMHHMVE KHRPTDAHLV
Cca21784                    ---------- ---------- ---------- ---------- ---------- ----------
Ava16635_4                  PGDHVEMNKL FTVPD-SISN TEITPLLDQH TGNVENEIEK TVDGHIHFPT RHGRKDANYL
Ava9904_1                   EKAEFLEEQL QNVLSVTYQG SDISEIPCVH SKALKKSDEI EIDTDFEYSC GNVKFEGAYE
Ava6620_1                   ---------- ---------- ---------- ---------- ---------- ----------

TRPC1_Hsap_NP_003295.1      ---------- ---------- ---------- ---------- ---------- ----------
TRPC1_Mmus_NP_035773.1      ---------- ---------- ---------- ---------- ---------- ----------
TRPC4 Hsap_NP_003297.1      ---------- ---------- ---------- ---------- ---------- ----------
TRPC4_Mmus_NP_058680.1      ---------- ---------- ---------- ---------- ---------- ----------
TRP_Spur XP_793901          ---------- ---------- ---------- ---------- ---------- ----------
TRP_Cint XP_002124651.2     ---------- ---------- ---------- ---------- ---------- ----------
TRP_Skow_XP_002733765.1     ---------- ---------- ---------- ---------- ---------- ----------
TRP_Lflor__emb|CAA11261.1   ---------- ---------- ---------- ---------- ---------- ----------
TRP_Bflo_XP_002611405.1     ---------- ---------- ---------- ---------- ---------- ----------
TRP4_Sman_XP_002576849.1    ---------- ---------- ---------- ---------- ---------- ----------
TRP_Nvec4_XP_001640409      ---------- ---------- ---------- ---------- ---------- ----------
TRP_Nvec1_XP_001637374.1    ---------- ---------- ---------- ---------- ---------- ----------
Cca7050                     HTLASERP-- ---------- ---------- ---------- ---------- ----------
Cca21784                    ---------- ---------- ---------- ---------- ---------- ----------
Ava16635_4                  IIPPQASPS- ---------D LLDVLSKTWK ---------- ----LPSPKI IISLPGTSQ-
Ava9904_1                   VADPAKKPKI VSITPKCEVN QLPKIHKYFK YDATHREYFV HHFRLCKPNL TLILPSTTDH
Ava6620_1                   ---------- ---------- ---------- ---------- ---------- ----------

TRPC1_Hsap_NP_003295.1      ---------- ---------- ---------- ---------- ---------- ----------
TRPC1_Mmus_NP_035773.1      ---------- ---------- ---------- ---------- ---------- ----------
TRPC4 Hsap_NP_003297.1      ---------- ---------- ---------- ---------- ---------- ----------
TRPC4_Mmus_NP_058680.1      ---------- ---------- ---------- ---------- ---------- ----------
TRP_Spur XP_793901          ---------- ---------- ---------- ---------- ---------- ----------
TRP_Cint XP_002124651.2     ---------- ---------- ---------- ---------- ---------- ----------
TRP_Skow_XP_002733765.1     ---------- ---------- ---------- ---------- ---------- ----------
TRP_Lflor__emb|CAA11261.1   ---------- ---------- ---------- ---------- ---------- ----------
TRP_Bflo_XP_002611405.1     ---------- ---------- ---------- ---------- ---------- ----------
TRP4_Sman_XP_002576849.1    ---------- ---------- ---------- ---------- ---------- ----------
TRP_Nvec4_XP_001640409      ---------- ---------- ---------- ---------- ---------- ----------
TRP_Nvec1_XP_001637374.1    ---------- ---------- ---------- ---------- ---------- ----------
Cca7050                     ---------- ---------- ---------- ---------- ---------- ----------
Cca21784                    ---------- ---------- ---------- ---------- ---------- ----------
Ava16635_4                  ------LSQG SRFRQMLKEN LIRIAATTKA WFLTEGVNKG ISAFVGSCLQ SHAYKRFAKK
Ava9904_1                   CPENAIRNRD PNYMKNFLSD MRQIIRNTDT WVLTNGLNWG LTRIIGENIA TRVLSPFTAE
Ava6620_1                   ---------- ---------- ---------- ---------- ---------- ----------

TRPC1_Hsap_NP_003295.1      ---------- ---------- ---------- ---------- ---------- -------ENT
TRPC1_Mmus_NP_035773.1      ---------- ---------- ---------- ---------- ---------- -------ENT
TRPC4 Hsap_NP_003297.1      ---------- ---------- ---------- ---------- ---------- -------ESE
TRPC4_Mmus_NP_058680.1      ---------- ---------- ---------- ---------- ---------- -------ESE
TRP_Spur XP_793901          ---------- ---------- ---------- ---------- ---------- -------MPG
TRP_Cint XP_002124651.2     ---------- ---------- ---------- ---------- ---------- ----------
TRP_Skow_XP_002733765.1     ---------- ---------- ---------- ---------- ---------- -------GNR
TRP_Lflor__emb|CAA11261.1   ---------- ---------- ---------- ---------- ---------- MSKKPPRKRD
TRP_Bflo_XP_002611405.1     ---------- ---------- ---------- ---------- ---------- -------ESD
TRP4_Sman_XP_002576849.1    ---------- ---------- ---------- ---------- ---------- ----------
TRP_Nvec4_XP_001640409      ---------- ---------- ---------- ---------- ---------- ----------
TRP_Nvec1_XP_001637374.1    ---------- ---------- ---------- ---------- ---------- ----------
Cca7050                     ---------- ---------- ---------- ---------- ---------- -------YDF
Cca21784                    ---------- ---------- ---------- ---------- ---------- ----------
Ava16635_4                  EQNQRDWFIG TGFGDYDAPI PIIGMVNPEN IYKGTLFQNP TDSITYGKDN SSLCFPDRNL
Ava9904_1                   T--------- ---------I ELIGIQKFEK L--PATVRNC ISRLSMKAQL EEDTIP-RGC
Ava6620_1                   ---------- ---------- ----FVKKDS HTVMARNEEL IPLREREEIQ EVLPIRHSDN

TRPC1_Hsap_NP_003295.1      LN--EKLFLL ACDKGDYYMV KK-------- ---------- ---------- ----------
TRPC1_Mmus_NP_035773.1      LN--EKLFLL ACDKGDYYMV KK-------- ---------- ---------- ----------
TRPC4 Hsap_NP_003297.1      LSPSEKAYLN AVEKGDYASV KK-------- ---------- ---------- ----------
TRPC4_Mmus_NP_058680.1      LSPSEKAYLN AVEKGDYASV KK-------- ---------- ---------- ----------
TRP_Spur XP_793901          SNHKEFGFIL ARSPMTETER PR-------- ---------- ---------- ----------
TRP_Cint XP_002124651.2     ---------- ---------- MV-------- ---------- ---------- ----------
TRP_Skow_XP_002733765.1     LTAKERMFME AAEKGDNATI QK-------- ---------- ---------- ----------
TRP_Lflor__emb|CAA11261.1   LNNEERQFLG AVARGDVGSV KQ-------- ---------- ---------- ----------
TRP_Bflo_XP_002611405.1     LSEAEKRFLV SVERGDYATV RK-------- ---------- ---------- ----------
TRP4_Sman_XP_002576849.1    ---------- ---------- ---------- ---------- ---------- ----------
```

```
TRP_Nvec4_XP_001640409      ---------- ---------- ---------- ---------- ---------- ----------
TRP_Nvec1_XP_001637374.1    ---------- ---------- ---------- ---------- ---------- ----------
Cca7050                     NTIRDSLFLH ACVHGSKATL QE-------- ---------- ---------- ----------
Cca21784                    ---------- ---------- ---------- ---------- ---------- ----------
Ava16635_4                  LDANHSHFIV LSKSISGNSL DQ-WSC---- ---------- ---------- --------IE
Ava9904_1                   LEENHSLFLS LKDNLDRDEI ETFWDT---- ---------- ---------- --------IE
Ava6620_1                   LTMKHWEYVE AARLDNREQQ PNFWKIPFAF GHSLVDDSKD DRLVTHYKHV PQKSIHQETK

TRPC1_Hsap_NP_003295.1      ---------- ---------- ---------- ---------- ---------- ----------
TRPC1_Mmus_NP_035773.1      ---------- ---------- ---------- ---------- ---------- ----------
TRPC4 Hsap_NP_003297.1      ---------- ---------- ---------- ---------- ---------- ----------
TRPC4_Mmus_NP_058680.1      ---------- ---------- ---------- ---------- ---------- ----------
TRP_Spur XP_793901          ---------- ---------- ---------- ---------- ---------- ----------
TRP_Cint XP_002124651.2     ---------- ---------- ---------- ---------- ---------- ----------
TRP_Skow_XP_002733765.1     ---------- ---------- ---------- ---------- ---------- ----------
TRP_Lflor__emb|CAA11261.1   ---------- ---------- ---------- ---------- ---------- ----------
TRP_Bflo_XP_002611405.1     ---------- ---------- ---------- ---------- ---------- ----------
TRP4_Sman_XP_002576849.1    ---------- ---------- ---------- ---------- ---------- ----------
TRP_Nvec4_XP_001640409      ---------- ---------- ---------- ---------- ---------- ----------
TRP_Nvec1_XP_001637374.1    ---------- ---------- ---------- ---------- ---------- ----------
Cca7050                     ---------- ---------- ---------- ---------- ---------- ----------
Cca21784                    ---------- ---------- ---------- ---------- ---------- ----------
Ava16635_4                  ET-------- ---------- ---------- ---------- ---------- ----------
Ava9904_1                   HTQIIKRIED IKAATCRESI ILDLEQKWDK NREAPNTPKT KTSLVELSTK RNSEVGEPKP
Ava6620_1                   DEGDLVKKPS TKFYDIVEIP ILDDPSNIDF LKYAKSLKQI LEVGKDLAYD RFNYRSKIDF

TRPC1_Hsap_NP_003295.1      ---------- ---------- ---------- ---------- ---------- ----------
TRPC1_Mmus_NP_035773.1      ---------- ---------- ---------- ---------- ---------- ----------
TRPC4 Hsap_NP_003297.1      ---------- ---------- ---------- ---------- ---------- ----------
TRPC4_Mmus_NP_058680.1      ---------- ---------- ---------- ---------- ---------- ----------
TRP_Spur XP_793901          ---------- ---------- ---------- ---------- ---------- ----------
TRP_Cint XP_002124651.2     ---------- ---------- ---------- ---------- ---------- ----------
TRP_Skow_XP_002733765.1     ---------- ---------- ---------- ---------- ---------- ----------
TRP_Lflor__emb|CAA11261.1   ---------- ---------- ---------- ---------- ---------- ----------
TRP_Bflo_XP_002611405.1     ---------- ---------- ---------- ---------- ---------- ----------
TRP4_Sman_XP_002576849.1    ---------- ---------- ---------- ---------- ---------- ----------
TRP_Nvec4_XP_001640409      ---------- ---------- ---------- ---------- ---------- ----------
TRP_Nvec1_XP_001637374.1    ---------- ---------- ---------- ---------- ---------- ----------
Cca7050                     ---------- ---------- ---------- ---------- ---------- ----------
Cca21784                    ---------- ---------- ---------- ---------- ---------- ----------
Ava16635_4                  ---------- -VSNHLKIGK EPL------- ---------- ---------- -----PIVRI
Ava9904_1                   DGDLIEMGKL EKANFVIFED EPTRVGRTID SGPRIPSEFQ NLWFKQIEFK DPDINPAVCL
Ava6620_1                   FNMIIYIPEI TKTGAGLLAV PNTAVAILGK VITDLLTKNK ALIILDARAK GPITNLVLES

TRPC1_Hsap_NP_003295.1      ---------- ---------- ----ILEENS SG-DLNINCV DVLG------ --------RN
TRPC1_Mmus_NP_035773.1      ---------- ---------- ----ILEENS SG-DLNINCV DVLG------ --------RN
TRPC4 Hsap_NP_003297.1      ---------- ---------- ----SLEEAE IYFKININCI DPLG------ --------RT
TRPC4_Mmus_NP_058680.1      ---------- ---------- ----SLEEAE IYFKININCI DPLG------ --------RT
TRP_Spur XP_793901          ---------- ---------- ---------- ---------- ---------- ----------
TRP_Cint XP_002124651.2     ---------- ---------- ----NLLRAF TNRNIGLGVI GFNG------ --------NV
TRP_Skow_XP_002733765.1     ---------- ---------- ----CLKHPD P---VNVNVT NILG------ --------RS
TRP_Lflor__emb|CAA11261.1   ---------- ---------- ----ALAEAE ERF-IDISCK DSLG------ --------RS
TRP_Bflo_XP_002611405.1     ---------- ---------- ----ILEDYG P--EMDINCT DPLS------ --------RT
TRP4_Sman_XP_002576849.1    ---------- ---------- ---------- ---------- --MG------ ----------
TRP_Nvec4_XP_001640409      ---------- ---------- ---------- ---------- ---------- ----------
TRP_Nvec1_XP_001637374.1    ---------- ---------- ---------- ---------- ---------- ----------
Cca7050                     ---------- ---------- ---------- ---------- ---------- ----------
Cca21784                    ---------- ---------- ---------- ---------- ---------- ----------
Ava16635_4                  VIDNTLHVIE QVGQSMNKNV ATV-VISSNE LMRKIHSYIF TLTV------ --------RD
Ava9904_1                   LLDNSIDGLR HTLHAIRNRV PTILVIGSGI AADMIHQIM- -LLS------ --------RD
Ava6620_1                   LERAKHDGVK QITERIQNRL NFL-GFYDKP LKQDIDPEAP NALGNKRYLL STDGCRELKS

TRPC1_Hsap_NP_003295.1      AVTITIENEN LDILQL---- ---------- ---------- --LLD-Y--- ----------
TRPC1_Mmus_NP_035773.1      AVTITIENES LDILQL---- ---------- ---------- --LLD-Y--- ----------
TRPC4 Hsap_NP_003297.1      ALLIAIENEN LELIEL---- ---------- ---------- --LLS-F--- ----------
TRPC4_Mmus_NP_058680.1      ALLIAIENEN LELIEL---- ---------- ---------- --LLS-F--- ----------
TRP_Spur XP_793901          ------TRGG IDIIVV---- ---------- ---------- --LLE-H--- ----------
TRP_Cint XP_002124651.2     AAGIAVELKD DLLIKL---- ---------- ---------- --IVVEH--- ----------
TRP_Skow_XP_002733765.1     ALQISVDNEN IEIVEL---- ---------- ---------- --LLAQP--- ----------
TRP_Lflor__emb|CAA11261.1   SLVIAIENEN DDLVSL---- ---------- ---------- --LLN-Y--- ----------
TRP_Bflo_XP_002611405.1     ALLIAIENEN LELVGL---- ---------- ---------- --LVS-Y--- ----------
TRP4_Sman_XP_002576849.1    ---------- ---------- ---------- ---------- --LIGRF--- ----------
TRP_Nvec4_XP_001640409      ---------- IEMIRL---- ---------- ---------- --LLK-N--- ----------
TRP_Nvec1_XP_001637374.1    ---------- ---------- ---------- ---------- ---------- ----------
Cca7050                     ---------- ---------- ---------- ---------- --LLQ----- ----------
Cca21784                    ---------- ---------- ---------- ---------- ---------- ----------
Ava16635_4                  QKMASTK--- QDFLKLCCGF LKNEAQSEEI LTNIGFILDK PNLFTMF--- ----------
```

```
Ava9904_1                    HEVISISNEV REYLKQNYPL QALRLPEDLL RRVISSVVDN EELFTFY--- ----------
Ava6620_1                    MASSSISKVL KGCIVLRHDH NDGKLVKEHL LYETSCLLKI PGLKIEFIVE PGKRTCHEVQ

TRPC1_Hsap_NP_003295.1       ---------- ---------- ---------- ---------- ---------- ----------
TRPC1_Mmus_NP_035773.1       ---------- ---------- ---------- ---------- ---------- ----------
TRPC4_Hsap_NP_003297.1       ---------- ---------- ---------- ---------- ---------- ----------
TRPC4_Mmus_NP_058680.1       ---------- ---------- ---------- ---------- ---------- ----------
TRP_Spur_XP_793901           ---------- ---------- ---------- ---------- ---------- ----------
TRP_Cint_XP_002124651.2      ---------- ---------- ---------- ---------- ---------- ----------
TRP_Skow_XP_002733765.1      ---------- ---------- ---------- ---------- ---------- ----------
TRP_Lflor__emb|CAA11261.1    ---------- ---------- ---------- ---------- ---------- ----------
TRP_Bflo_XP_002611405.1      ---------- ---------- ---------- ---------- ---------- ----------
TRP4_Sman_XP_002576849.1     ---------- ---------- ---------- ---------- ---------- ----------
TRP_Nvec4_XP_001640409       ---------- ---------- ---------- ---------- ---------- ----------
TRP_Nvec1_XP_001637374.1     ---------- ---------- ---------- ---------- ---------- ----------
Cca7050                      ---------- ---------- ---------- ---------- ---------- ----------
Cca21784                     ---------- ---------- ---------- ---------- ---------- ----------
Ava16635_4                   ---------- ---------- ---------- ---------- ---------- ----------
Ava9904_1                    ---------- ---------- ---------- ---------- ---------- ----------
Ava6620_1                    IDFLDPHYKE TDDRVYNFYR KEPELYQMIL QKADEYFYRD ESDFKGWNTM TTALFLRDQY

TRPC1_Hsap_NP_003295.1       ---------- --------GC Q--------- ---------- ---------- ----------
TRPC1_Mmus_NP_035773.1       ---------- --------GC QSADALLVAI DS-------- ---------- ----------
TRPC4_Hsap_NP_003297.1       ---------- --------NV YVGDALLHAI RK-------- ---------- ----------
TRPC4_Mmus_NP_058680.1       ---------- --------NV YVGDALLHAI RK-------- ---------- ----------
TRP_Spur_XP_793901           ---------- --------GI QLGDALLRAV DE-------- ---------- ----------
TRP_Cint_XP_002124651.2      ---------- --LIKQPSMA RMGDALLLAI SK-------- ---------- ----------
TRP_Skow_XP_002733765.1      ---------- --------NV KIGDALLYAI RE-------- ---------- ----------
TRP_Lflor__emb|CAA11261.1    ---------- --------DV DLEDSLLHAI RE-------- ---------- ----------
TRP_Bflo_XP_002611405.1      ---------- --------NV RVGDALLHAI KR-------- ---------- ----------
TRP4_Sman_XP_002576849.1     ---------- ---------- ---------- ---------- ---------- ----------
TRP_Nvec4_XP_001640409       ---------- --------GA DVGAALLQAV SK-------- ---------- ----------
TRP_Nvec1_XP_001637374.1     ---------- ---------- ---------- ---------- ---------- ----------
Cca7050                      ---------- --LGASIDGK DGASGLPVML LAASSAVNQT AIENVNILMA NGADLHVTSS
Cca21784                     ---------- ---------- ---------- ---------- ---------- ----------
Ava16635_4                   ---------- --DMNMTYGA DLSKAIVVAL LKSRKSTGKE KLKEALDLVI DWNRVDLAEQ
Ava9904_1                    ---------- --HPLRHGIH ELNKAILLAL LKG--YPGDN KRLYGLILSF RWKRPSLALE
Ava6620_1                    RVRRALERRT REESVKFDQN QIGRIIRYAL FYGSREIVEN IF-------- ----------

TRPC1_Hsap_NP_003295.1       ---------- ---------- ---------- ---------- ---------- ------KLME
TRPC1_Mmus_NP_035773.1       ---------- ---------- --------EV VGAVDILLNH -----RPKRS SRPTIVKLME
TRPC4_Hsap_NP_003297.1       ---------- ---------- --------EV VGAVELLLNH ------KKPS GEKQVPPI--
TRPC4_Mmus_NP_058680.1       ---------- ---------- --------EV VGAVELLLNH ------KKPS GEKQVPPI--
TRP_Spur_XP_793901           ---------- ---------- --------QF IYAAQIICEH IKQKNIPEFL ----------
TRP_Cint_XP_002124651.2      ---------- ---------- --------GY LRIAEILMNH PSFCVNQRLT TSPGELMLID
TRP_Skow_XP_002733765.1      ---------- ---------- --------GV YKMVEMMVNH PS--ISREML GGEWS-KMTK
TRP_Lflor__emb|CAA11261.1    ---------- ---------- --------EY VVAVEMILTH -----QLKKF GEDYL-----
TRP_Bflo_XP_002611405.1      ---------- ---------- --------EF VGAVELLLN- ---------C TEDKTQYM--
TRP4_Sman_XP_002576849.1     ---------- ---------- ---------- ---------- ---------- ----------
TRP_Nvec4_XP_001640409       ---------- ---------- --------DN VLYVKALLAY EN--NN-DRF SRRSSSCV--
TRP_Nvec1_XP_001637374.1     ---------- ---------- ---------- ---------- ---------- ----------
Cca7050                      N--------- -------GQN LCHIAAILCN VQLLELAIA- ---------- ----------
Cca21784                     ---------- ---------- ---------- ---MQTAID- ---------- ----------
Ava16635_4                   NIFTESMVW- --GDTDLFQH -YFAILILNQ VDFLELMLER ---------- ----------
Ava9904_1                    KIFIGREKWI SEDDTRAVQR IFFSALTTNN TGFVHRMLES ---------- ----------
Ava6620_1                    ---------- ---------- --------NN SDIQQRLIKK ---------- ----------

TRPC1_Hsap_NP_003295.1       ------RIQN PEYSTTMDVA PVILAAH--- RNNYEILTML LKQDVSLPKP HAVGCECTLC
TRPC1_Mmus_NP_035773.1       ------RIQN PEYSTTMDVA PVILAAH--- RNNYEILTML LKQDVSLPKP HAVGCECTLC
TRPC4_Hsap_NP_003297.1       ------LLDK QFSEFTPDIT PIILAAH--- TNNYEIIKLL VQKGVSVPRP HEVRCNCVEC
TRPC4_Mmus_NP_058680.1       ------LLDK QFSEFTPDIT PIILAAH--- TNNYEIIKLL VQKGVSVPRP HEVRCNCVEC
TRP_Spur_XP_793901           ------KCRA LNGDFHPDIT PIVLAAH--- HNNYDIIKIL LEYGARIEDP EYY-------
TRP_Cint_XP_002124651.2      PNSDFYAYDN DGTRFSPDIT PIILAAQ--- CQEFDIVYEL IRRGATIQHP HPYRCQCTEC
TRP_Skow_XP_002733765.1      ------DPQE ESSDYSPDIS PVILAAH--- CNQFEILQLL LTRGATISTP HNVTCGCEHC
TRP_Lflor__emb|CAA11261.1    ------QIEF KSNTFTADIT PIILAGH--- IDNYEIIKML LDRGYRIPKP HDLTCHCDDC
TRP_Bflo_XP_002611405.1      ------KYVQ KESDFTEDMT PIILAAH--- KNNYEILKLL LQKGHPIPKP HDVRCTCNEC
TRP4_Sman_XP_002576849.1     ---------- QSSSFTPDIN GIILAAH--- RDNYVILKLL LDRGDRIYKP HDLRCACNIC
TRP_Nvec4_XP_001640409       ------NLSK NTNDSNRYMT PLILASH--- NNSYDIVKLL LSKGHTIDRV HDRGCLCPWC
TRP_Nvec1_XP_001637374.1     ---------- ---------- PLVVAAQ--- LGNYEVLNML VSKGFQLVKP HNVLCRCEEC
Cca7050                      ------ARVD HTYQDKEGST PLHYACA--A PENDAVISLL IEAGLDMNEP NSDGLTPLNL
Cca21784                     ------KGVD HTKSDREGNT PLHYAAA-AV DDNHEVIELL IGDACDTNAA NSKAQTPLCF
Ava16635_4                   ------NVID HAAFMKQNLE RLYFVANDRK RNNDLLFKQL VDRSNIDQHP NVPHSTIKTL
Ava9904_1                    ------KIIN FRYF-----G NMYYLSQ--- -----LYKRT FDKLSDSEED IIANKMLRLN
Ava6620_1                    ------REEN LTSVFKKSGQ ELILFQR--- ------LMFA LTKDEEYVEK KIKGCKAEFT

TRPC1_Hsap_NP_003295.1       SAKNKKDSL- ---------- ---------- ---------- ---------- ----------
TRPC1_Mmus_NP_035773.1       SAKNKKDSL- ---------- ---------- ---------- ---------- ----------
```

```
TRPC4_Hsap_NP_003297.1      VSSSDVDSL- ---------- ---------- ---------- ---------- ----------
TRPC4_Mmus_NP_058680.1      VSSSDVDSL- ---------- ---------- ---------- ---------- ----------
TRP_Spur_XP_793901          AFSTETNTL- ---------- ---------- ---------- ---------- ----------
TRP_Cint_XP_002124651.2     TAMRRDDAL- ---------- ---------- ---------- ---------- ----------
TRP_Skow_XP_002733765.1     TEKQHEDSL- ---------- ---------- ---------- ---------- ----------
TRP_Lflor__emb|CAA11261.1   LRGSLVDVL- ---------- ---------- ---------- ---------- ----------
TRP_Bflo_XP_002611405.1     AEGYRTDAL- ---------- ---------- ---------- ---------- ----------
TRP4_Sman_XP_002576849.1    TKARRENGL- ---------- ---------- ---------- ---------- ----------
TRP_Nvec4_XP_001640409      QSLGR---I- ---------- ---------- ---------- ---------- ----------
TRP_Nvec1_XP_001637374.1    ----QADNF- ---------- ---------- ---------- ---------- ----------
Cca7050                     SIKSQHVNNV IPLIRAMVDI TKC------- ---------- ---------- ----------
Cca21784                    AVLSNQVTNI VPLIRSSADI RKA------- ---------- ---------- ----------
Ava16635_4                  KPQNKNSNLP NQT------- ---------- --NGQIPEDQ QKIPIAE--- ----------
Ava9904_1                   FSESKHRKDP RQILYEIGKL IKELMGNDYV CFYNHLPISR KKREYEEENI KKNVNSCYNF
Ava6620_1                   ASLE------ ---------- ---------- ---------- ---------- ----------

TRPC1_Hsap_NP_003295.1      ---------- ---------- ---------- ---------- RHSRFRLDIY RCLASP---A
TRPC1_Mmus_NP_035773.1      ---------- ---------- ---------- ---------- RHSRFRLDIY RCLASP---A
TRPC4_Hsap_NP_003297.1      ---------- ---------- ---------- ---------- RHSRSRLNIY KALASP---S
TRPC4_Mmus_NP_058680.1      ---------- ---------- ---------- ---------- RHSRSRLNIY KALASP---S
TRP_Spur_XP_793901          ---------- ---------- ---------- ---------- QHSLGMLNIY RALASQ---A
TRP_Cint_XP_002124651.2     ---------- ---------- ---------- ---------- NFCLSRLNAY KGLASP---V
TRP_Skow_XP_002733765.1     ---------- ---------- ---------- ---------- RHSLHRINTY KALASP---A
TRP_Lflor__emb|CAA11261.1   ---------- ---------- ---------- ---------- GHSRARINAY RALASP---S
TRP_Bflo_XP_002611405.1     ---------- ---------- ---------- ---------- RHSRQRLNVY RALASP---S
TRP4_Sman_XP_002576849.1    ---------- ---------- ---------- ---------- QHSTLRINTY RALTSP---S
TRP_Nvec4_XP_001640409      ---------- ---------- ---------- ---------- GTSLHRLYTY RALASPVYMS
TRP_Nvec1_XP_001637374.1    ---------- ---------- ---------- ---------- RESQRRLDIY RGLANP---V
Cca7050                     ---------- ---------- ---------- ---------- GLQDCIYQDL QILAQP---E
Cca21784                    ---------- ---------- ---------- ---------- GLQKLRIEKL QLLARP---E
Ava16635_4                  ---IGKLLKY VLK------- --YSFKDVYV ENEGMHDIIE NHPIDNLFIW AVLTQRWRMA
Ava9904_1                   LRHIATLMKQ CLKRFRVRKL TAQTKKLKLV RAEKAQRLFE QFPEEQVFIW CLLFHRWEMA
Ava6620_1                   ---VVKFLQL AIEKLMGDGF LWNIPDVQRH DMQLNLTHYE VYFAEYIMVW GALSQRYEVA

TRPC1_Hsap_NP_003295.1      LIMLT----- ---------- ---------- -----EEDPI LRAFELSADL K---------
TRPC1_Mmus_NP_035773.1      LIMLT----- ---------- ---------- -----EEDPI LRAFELSADL K---------
TRPC4_Hsap_NP_003297.1      LIALS----- ---------- ---------- -----SEDPF LTAFQLSWEL Q---------
TRPC4_Mmus_NP_058680.1      LIALS----- ---------- ---------- -----SEDPF LTAFQLSWEL Q---------
TRP_Spur_XP_793901          YISLT----- ---------- ---------- -----SLDPI NTGFERCVKL R---------
TRP_Cint_XP_002124651.2     YMSIV----- ---------- ---------- -----TRDPV RHALKLCHEL S---------
TRP_Skow_XP_002733765.1     WISLT----- ---------- ---------- -----SSDPI LTAFKLSWEL E---------
TRP_Lflor__emb|CAA11261.1   LIALS----- ---------- ---------- -----SKDPI LTAFELSWEL R---------
TRP_Bflo_XP_002611405.1     LIMLS----- ---------- ---------- -----SDDPI LTAFQLSWEL RGSVTTRLFN
TRP4_Sman_XP_002576849.1    FIILT----- ---------- ---------- -----SNDPI LTAFELSWEL K---------
TRP_Nvec4_XP_001640409      LTFLS----- ---------- ---------- -----CKDPV RQAFELNKEL V---------
TRP_Nvec1_XP_001637374.1    FISMT----- ---------- ---------- -----SNDPF LTAFELSSEL E---------
Cca7050                     IIEA------ ---------- ---------- -----SPKPL EYTLMLSSFF E---------
Cca21784                    MIQA------ ---------- ---------- -----SAEPL KFVLELSYLY R---------
Ava16635_4                  EVLLSNTNSV I--------- ---------- FNAV-AAKAL IAGMHHHLKD D---------
Ava9904_1                   ILILKYCNKT C--------- ---------- LTTVLSAARL IRGMKLKYQS R---------
Ava6620_1                   ELFLEYSGGH LQELLTRDRE FYRTQKGVYQ IANALAISRL LYGVEQELRF D---------

TRPC1_Hsap_NP_003295.1      ---------- ---ELSLVEV EFRNDYEELA RQCKMFAKDL LAQ---ARNS RELEV--ILN
TRPC1_Mmus_NP_035773.1      ---------- ---ELSLVEV EFRNDYEELA RQCKMFAKDL LAQ---ARNS RELEV--ILN
TRPC4_Hsap_NP_003297.1      ---------- ---ELSKVEN EFKSEYEELS RQCKQFAKDL LDQ---TRSS RELEI--ILN
TRPC4_Mmus_NP_058680.1      ---------- ---ELSKVEN EFKSEYEELS RQCKQFAKDL LDQ---TRSS RELEI--ILN
TRP_Spur_XP_793901          ---------- ---KLCQINP EFSVEFNTLA GQCEQFAADI LGH---IRNQ AEQTC--VLY
TRP_Cint_XP_002124651.2     ---------- ---KMATMEK EFKKDYLKLV HQLYDYLEKL LDI---CRNS EEVNA--MLS
TRP_Skow_XP_002733765.1     ---------- ---HLAMREN EFKDVYQQLS HQSKTYAVDL LEQ---CRSS EEVIA--VLN
TRP_Lflor__emb|CAA11261.1   ---------- ---NLSEMEN EFRAEYEELG ITCQNFAVDL LEQ---IRGS KELEV--ILN
TRP_Bflo_XP_002611405.1     SFHHQFLAKT FLLTLSTLEN EFKSEYEQLS KQCKKFASDL LSH---TRGT RELQV--ILN
TRP4_Sman_XP_002576849.1    ---------- ---KLGELEN EFRTEYEELE AKCQKFATEM LAQ---TRSS SELSI--VLN
TRP_Nvec4_XP_001640409      ---------- ---ELADIEY EFRSDYLQLS QQCEEFAVAL LDQ---CRDM SEIEQ--MMS
TRP_Nvec1_XP_001637374.1    ---------- ---VIARRED EYEKDYLQLS QQCSQFTIGL LDE---CRTS REQRC--ILN
Cca7050                     ---------- ---RMAKERM RLKESYMALS VQLEDAAIDL IEG---LHSS QLQGL--VLH
Cca21784                    ---------- ---HWGYNHP LQKARYEALS MEMEELAVSM VDS---APPV LLLKV--LTN
Ava16635_4                  ---------- -----QIVSE NDLGLLVEVA DKFEMTAVGM MEE-AYKRDK EQAHL--ILQ
Ava9904_1                   ---------- -----TDVDE LARDRLENKA NDFENLAITL LGEFSYSAHS DLKLK--LLL
Ava6620_1                   ---------- -----TAVST DDRETLIQKA AYFEELAVGL LDN---AIGK NEFTLNELFN

TRPC1_Hsap_NP_003295.1      ---------- ------HTSS -D-------- --EPLDKRGL LEE------- ----------
TRPC1_Mmus_NP_035773.1      ---------- ------HTSS -D-------- --EPLDKRGL LEE------- ----------
TRPC4_Hsap_NP_003297.1      ---------- ------YRDD ---------- --NSLIEE-- -QS------- ----------
TRPC4_Mmus_NP_058680.1      ---------- ------YRDD ---------- --NSLIEE-- -QS------- ----------
TRP_Spur_XP_793901          ---------- ------HDPC ---------- -FYSIGES-- ---------- ----------
TRP_Cint_XP_002124651.2     GNYSDSWKGL ARKQSGRTSS NEITNNHLMK SFSNVADE-- HID------- ----------
TRP_Skow_XP_002733765.1     ---------- ------RDND SE-------- --DGFAEEDV MGG------- ----------
```

```
TRP_Lflor__emb|CAA11261.1   ---------- ------HDCR ---------- -SPDQDEE-- -KE------- ----------
TRP_Bflo_XP_002611405.1     ---------- ------HSDD ---------- ----MVES-- ATS------- ----------
TRP4_Sman_XP_002576849.1    ---------- ------HYTG ADVSGCHVSG INQSLSED-- ISE------- ----------
TRP_Nvec4_XP_001640409      ---------- ------VPDM -D-------- --GALTKN-- --G------- ----------
TRP_Nvec1_XP_001637374.1    ---------- ------YPGE ---------- -NGSVEDYA- ---------- ----------
Cca7050                     ---------- ---------- ---------- ---------- ---------- ----------
Cca21784                    ---------- ---------- ---------- ---------- ---------- ----------
Ava16635_4                  ---------- ------QNNR L--------- ---------- ---------- ----------
Ava9904_1                   ---------- ------KNRR VECSELIGLE VAACQDDKPP VEGDNLLMRE QDDSTTYIWP
Ava6620_1                   ---------- ---------- ---------- ---------- ---------- -----TKIPF

TRPC1_Hsap_NP_003295.1      RMNLSRLKLA IKYNQKEFVS QSNCQQFLNT VW-------- -FGQMSGYRR KPTCKKIMTV
TRPC1_Mmus_NP_035773.1      RMNLSRLKLA IKYNQKEFVS QSNCQQFLNT VW-------- -FGQMSGYRR KPTCKKIMTV
TRPC4_Hsap_NP_003297.1      GNDLARLKLA IKYRQKEFVA QPNCQQLLAS RW-------- -YDEFPGWRR RHWAVKMVTC
TRPC4_Mmus_NP_058680.1      GNDLARLKLA IKYRQKEFVA QPNCQQLLAS RW-------- -YDEFPGWRR RHWAVKMVTC
TRP_Spur_XP_793901          GIGPYKVKIA IHHEQRKFVA HPHCQQHLTQ LW-------- -YEGLPSWYM TNWISSTLST
TRP_Cint_XP_002124651.2     HPRLYQLKLA IKYGVKGFVA NPNCQLQLIA IW-------- -NRDLSWLRQ LSIGYKFLLA
TRP_Skow_XP_002733765.1     KITLIRLKLA LKFEQKRFVA HAHTQQLLTS IW-------- -YEGLPGWRK HNAFVKLLIC
TRP_Lflor__emb|CAA11261.1   LKKLSRLKLA IKYKQKKFVA HPNCQQLLAA KW-------- -YEGLGNFRR KPILKQLTVA
TRP_Bflo_XP_002611405.1     SNSLARLKLA IKYHQKEFVS QPNCQQLLSS MW-------- -YEGFPSWRR KHWAVKVLIC
TRP4_Sman_XP_002576849.1    RMQLSRLKLA IRYGQKQFVA HPHCQQLLAA IW-------- -YEGLPGFRQ KPVFAQLTTI
TRP_Nvec4_XP_001640409      SKSLGVLDYA INNKNGRFVA HPYCQMMLNS VI-------- -YGDFHTWEK MGVTLKILFA
TRP_Nvec1_XP_001637374.1    ENSLGLVNSA ISYNQKEFVA HPFCQHLVMQ HI-------- -FGDITGWRT NHFLYRIAYV
Cca7050                     ---GNVLEMA ISTGRKRFIA TDAVQYELNR VW-------- -HE------W QEPSYKALLK
Cca21784                    ----EIVEMA ISDRCKKFIA HASVLQALKR SW-------- -AG------E NSLPLRLILS
Ava16635_4                  YDDNNCIEMA VTGRCMTFLS HPCVQSLLDE QW-------- -KKPL---YH QNKLWKKLLS
Ava9904_1                   WHENNAFDMA IKGDCKHFIA HPLIQDYANQ KW-------- -YSKI---RR LNKEWKLIMC
Ava6620_1                   WNHSTAIELA STAGKQLFFT HEGVQGHVRN VWNGIAKLQE QYEKTNSDQN INIGVQLKNS

TRPC1_Hsap_NP_003295.1      LTVGIFWPVL S---LCYLIA PKS------- ---------- ---------- --------QF
TRPC1_Mmus_NP_035773.1      LTVGIFWPVL S---LCYLIA PKS------- ---------- ---------- --------QF
TRPC4_Hsap_NP_003297.1      FIIGLLFPVF S---VCYLIA PKS------- ---------- ---------- --------PL
TRPC4_Mmus_NP_058680.1      FIIGLLFPVF S---VCYLIA PKS------- ---------- ---------- --------PL
TRP_Spur_XP_793901          SAIGFGFPIL C---LLYIIY PWG------- ---------- ---------- --------NI
TRP_Cint_XP_002124651.2     VGMSLFMPVL S---IAYFVA PRS------- ---------- ---------- --------KV
TRP_Skow_XP_002733765.1     LAVILLLPLM A---LCYLLF PHT------- ---------- ---------- --------KI
TRP_Lflor__emb|CAA11261.1   FGILFGFPIL S---GIYMIA PHS------- ---------- ---------- --------KF
TRP_Bflo_XP_002611405.1     ASIALAFPLL G---ICYLCA PKS------- ---------- ---------- --------SL
TRP4_Sman_XP_002576849.1    GTLCSLFPLL A---TFYMLA PHS------- ---------- ---------- --------KL
TRP_Nvec4_XP_001640409      LLFTIFIPVW A---IVYFFT PTS------- ---------- ---------- --------CL
TRP_Nvec1_XP_001637374.1    LTQVIIFPVL A---VIYFFM PFL------- ---------- ---------- --------EV
Cca7050                     STIIFLLQAI ARPFVILVVW PLI------- ---------- -----VLNIQ VFGYTFSWNI
Cca21784                    LTFVFILKAM VRPFVFFCIW PFL------- ---------- -------LVR DSCCCITWDS
Ava16635_4                  LIFPFLIYFL I---SFSDPQ PSLP------ ---------- ---------- -------LGY
Ava9904_1                   FFIPFLIPFV L---ELHSRR KRLEHHMNPQ KDPVTKETRF RQKSFCHFFL FLFFDFFRGI
Ava6620_1                   LRHPFSKKSL N---FFY--- ---------- ---------- ---------- ----------

TRPC1_Hsap_NP_003295.1      GRIIHTPFMK FIIHGASYFT FLLLLNLYSL V--------- ---------- ----------
TRPC1_Mmus_NP_035773.1      GRIIHTPFMK FIIHGASYFT FLLLLNLYSL V--------- ---------- ----------
TRPC4_Hsap_NP_003297.1      GLFIRKPFIK FICHTASYLT FLFLLLLASQ H--------- ---------- ----------
TRPC4_Mmus_NP_058680.1      GLFIRKPFIK FICHTASYLT FLFLLLLASQ H--------- ---------- ----------
TRP_Spur_XP_793901          GRLMRVPHVQ FVCHISSMLV FLLLLGLQSS ---------- ---------- ------FTPK
TRP_Cint_XP_002124651.2     VRTMKSPFCK FATQGMSYAQ FLMLLILNCM DRVEGFEFSS T--------- ------FTFL
TRP_Skow_XP_002733765.1     GQLLRTPFMK FMNHSISFGF FLLLLVLAST VRFEGTRPAS PSDTWVD--- ----------
TRP_Lflor__emb|CAA11261.1   GKLCRKPFIK FLLHSASYLS FLVLLILVSQ R--------- ---------I ENPFFFLFPM
TRP_Bflo_XP_002611405.1     GKLIRKPFLK FICHTTSYLF FLALLYLASN PSLVGDEADD RPDQQGPVPN TVEWMIFVWV
TRP4_Sman_XP_002576849.1    GSMMRKPFIK FLCHSSSYLS FLALLTLAAT R--------- ---------- ---VEFLLTN
TRP_Nvec4_XP_001640409      SQKLATPILK FIMHTGAFGL FLLLLILSSI QRFISSNT-- ---------- ----------
TRP_Nvec1_XP_001637374.1    GRKIKRPFVK FINHTSSFVV FLILLAVSSH HQFE------ ---------- ----------
Cca7050                     LRPRAAPTIR YRVEAFAYVA FLILLMVEAA DH-------- ---------- ----------
Cca21784                    LRPIAEPIII YTAHVISYSV FVVLLLIDAL KL-------- ---------- ----------
Ava16635_4                  LKFYTSPSIK FITYVLAFIL YLTLYSFVVL FQWHPSPN-- ---------- ----------
Ava9904_1                   YSFYNAPKIK YWLHFLSHLV YTILFTIVGV TPYRPIRNRT T--------- ----------
Ava6620_1                   -TYYISPSTT FYIHSLVYFI FLVLYTVHVL FTWEPSHKNG ---------- ----------

TRPC1_Hsap_NP_003295.1      YNEDKK-NTM GPALERIDYL LILWIIGMI- ---------- ---------- ----------
TRPC1_Mmus_NP_035773.1      YNEDKK-NTM GPALERIDYL LILWIIGMI- ---------- ---------- ----------
TRPC4_Hsap_NP_003297.1      IDRSDL-NRQ GPPPTIVEWM ILPWVLGFI- ---------- ---------- ----------
TRPC4_Mmus_NP_058680.1      IDRSDL-NRQ GPPPTIVEWM ILPWVLGFI- ---------- ---------- ----------
TRP_Spur_XP_793901          YDDDPDVDQR AEVPKPTEWL ILHGMAMELM EIPPTTMLYS TMEIPTAFTD DEFTTSGETP
TRP_Cint_XP_002124651.2     LHQTSR-DGR HTQFNWCEVF IMLYIAGLL- ---------- ---------- ----------
TRP_Skow_XP_002733765.1     -------SPR GREPNFIEVL IALWVFGFI- ---------- ---------- ----------
TRP_Lflor__emb|CAA11261.1   KEEKKISELR GSPATIVELM ILGYVIGLI- ---------- ---------- ----------
TRP_Bflo_XP_002611405.1     LERRQV-RPA EPVPNTVEWM IFVWVLGMI- ---------- ---------- ----------
TRP4_Sman_XP_002576849.1    SLDQRM-HDR GPTPSLTESA LVIYVI---- ---------- ---------- ----------
TRP_Nvec4_XP_001640409      -------FLL KFLFNFSDIL ITIWIFGLI- ---------- ---------- ----------
TRP_Nvec1_XP_001637374.1    -------IRF RKMPSGLEWL IFSWILGVA- ---------- ---------- ----------
```

```
Cca7050                     -------EFS KPFMTKLEWI TLAYVLALL- ---------- ---------- ----------
Cca21784                    -------DRI EAILDGLLLV IFIFVFGLI- ---------- ---------- ----------
Ava16635_4                  ---------- ---LNPCEWI FIVWSLTYI- ---------- ---------- ----------
Ava9904_1                   -------DIA NDPLLIIELI VWVWTFTII- ---------- ---------- ----------
Ava6620_1                   ---------- ---FVTFDVI YFLYMVAYI- ---------- ---------- ----------

TRPC1_Hsap_NP_003295.1      ---------- ----W----- ---------- ------SDIK RLW------- --YEGLEDFL
TRPC1_Mmus_NP_035773.1      ---------- ----W----- ---------- ------SDIK RLW------- --YEGLEDFL
TRPC4 Hsap_NP_003297.1      ---------- ----W----- ---------- ------GEIK QMW------- --DGGLQDYI
TRPC4_Mmus_NP_058680.1      ---------- ----W----- ---------- ------GEIK QMW------- --DGGLQDYI
TRP_Spur XP_793901          VTVTDPATTM EMSNWEAVIV RIERLHKDVM DIDEEQTDIG QSL------- --AKGIGD-I
TRP_Cint XP_002124651.2      ---------- ----W----- ---------- ------NEVR ELW------- --DEGPRNYI
TRP_Skow XP_002733765.1      ---------- ----W----- ---------- ------GECK QLW------- --EEGLKAYI
TRP_Lflor__emb|CAA11261.1   ---------- ----W----- ---------- ------SEIK QIW------- --MQGALEYI
TRP_Bflo XP_002611405.1      ---------- ----W----- ---------- ------GEIK QLW------- --EAGLKEYI
TRP4_Sman XP_002576849.1    ---------- ---------- ---------- ---------- ---------- ------ERFF
TRP_Nvec4 XP_001640409      ---------- ----V----- ---------- ------QELK DMW------- --RQGKERYL
TRP_Nvec1 XP_001637374.1    ---------- ----W----- ---------- ------SECK QVW------- --HEGARRYF
Cca7050                     ---------- ----Y----- ---------- ------NEFV TLV------- --REGFHFYF
Cca21784                    ---------- ----L----- ---------- ------QEIR SFL------- --RLERRFYF
Ava16635_4                  ---------- ----L----- ---------- ------EEVR QLFAGNGRVM KR---VSAYL
Ava9904_1                   ---------- ----M----- ---------- ------EECS QLLHEKGSLL NR---IRGYI
Ava6620_1                   ---------- ----LDEVFQ YRFLIEDKTK GKHIKHNQYI RIL------- --NSALFEYL

TRPC1_Hsap_NP_003295.1      EESRNQLSFV MNSLY----- ----LATFAL KVVAHNKFH- ---------- ----------
TRPC1_Mmus_NP_035773.1      EESRNQLSFV MNSLY----- ----LATFAL KVVAHNKFH- ---------- ----------
TRPC4 Hsap_NP_003297.1      HDWWNLMDFV MNSLY----- ----LATISL KIVAFVKYS- ---------- ----------
TRPC4_Mmus_NP_058680.1      HDWWNLMDFV MNSLY----- ----LATISL KIVAFVKYS- ---------- ----------
TRP_Spur XP_793901          KSQLTLMDER MAKIE----- ----AESEAA AVAAPVVSGG RRGKKAAAGT ATNT------
TRP_Cint XP_002124651.2      IQLWNVLDFF MLLIL----- ----ITSFAS SFISHRNSWI AQQRWDEIFK ENATFVECDH
TRP_Skow XP_002733765.1      RQWWNWLDFI MLSLY----- ----LCTISL RFTAWWLKKS GT-------- ----------
TRP_Lflor__emb|CAA11261.1   HDMWNIMDLV TNSLY----- ----MATFTL RLLAWLQVRR ERAE------ ----------
TRP_Bflo XP_002611405.1      SDQWNVMDFI MNSLY----- ----VATIAL RVVSYIKFKD ETLT------ ----------
TRP4_Sman XP_002576849.1    RLKGNSIWLL YNLCYKKGVL WKTKRSTRQI KIVFYNEP-- ---------- ----------
TRP_Nvec4 XP_001640409      SQWWNLVTLA MLGLF----- ----IISGIL WVAGSALS-- ---------- ----------
TRP_Nvec1 XP_001637374.1    SSGWNWMDIG MVFLL----- ----LGAFVL WTIIFIV--- ---------- ----------
Cca7050                     TRLGHKTNCI IITMF----- ----IIFYIV RLIVIYS--- ---------- ----------
Cca21784                    SGLDYKMDIS MLVLF----- ----VLFFIL RAIAYFT--- ---------- ----------
Ava16635_4                  NDRWNQFDTA FALFF----- ----IMGTVL RLIPQVPLS- ---------- ----------
Ava9904_1                   SSAWNILDLA LILLF----- ----LLAFSI KLGCDCS-A- ---------- ----------
Ava6620_1                   SSGWNILDIA IAIGF----- ----VATISL RISVFII--- ---------- ----------

TRPC1_Hsap_NP_003295.1      ---------- ---------- -----DFADR KDWDAFHPTL V--AEGLFAF ANVLSYLRLF
TRPC1_Mmus_NP_035773.1      ---------- ---------- -----DFADR KDWDAFHPTL V--AEGLFAF ANVLSYLRLF
TRPC4 Hsap_NP_003297.1      ---------- ---------- -----ALNPR ESWDMWHPTL V--AEALFAI ANIFSSLRLI
TRPC4_Mmus_NP_058680.1      ---------- ---------- -----ALNPR ESWDMWHPTL V--AEALFAI ANIFSSLRLI
TRP_Spur XP_793901          ---------- ---------- -----TRTGR ANWSAYDPLL I--SEGLFAI AKVLSFLRPI
TRP_Cint XP_002124651.2      MSGNITLFGQ LVNVDVPRWM CYYSYKHADR ANWYGSDPQL I--AEALYSF GIVLSFTRIC
TRP_Skow XP_002733765.1      ---------- ---------Y PSFTDGEVQR ATWPGDDPTL I--SEGVFAV ANVFSFFRII
TRP_Lflor__emb|CAA11261.1   ---------- ---------- -LNPYAFEER KHWDAYDPNL I--AEALFAG ANIFSSLKLI
TRP_Bflo XP_002611405.1      ---------- ---------- -----GIHPR STWQADHPTL V--AEGLFAT ANIFSSLRVI
TRP4_Sman XP_002576849.1    ---------- ---------- -----RYINR GQWDSFDPVL V--SECLFAA ANIVSTLKLV
TRP_Nvec4 XP_001640409      ---------- ---------- -----VSKEG SLAESYRFIL L--ANAFFTI GVLLAFLHFS
TRP_Nvec1 XP_001637374.1    ---------- ---------- -----DFDSN AKLAHDVLLS T--ADGMYAF GVVASFFRLI
Cca7050                     ---------- ---------- ---------- KVESPNDVLR L--TSYLLAV ASTMSCIRLL
Cca21784                    ---------- ---------- ---------- ----TDNLVL LRGSCYFLGP ATVLACVRVL
Ava16635_4                  ---------- ---------- -----Y---- ---------- ---SRHMYAV IGFVLFFRLL
Ava9904_1                   ---------- ---------- -----W---- ---------- ---VHRVYAL IILILYIRIF
Ava6620_1                   ---------- ---------- -----YDQTN GGENARNLNV I--ARLVFGL FGVFLYMRFF

TRPC1_Hsap_NP_003295.1      FMYTTSSILG PLQISMGQML QDFGKFLGMF LLVLFSFTIG LTQLYDKGYT SKEQ------
TRPC1_Mmus_NP_035773.1      FMYTTSSILG PLQISMGQML QDFGKFLGMF LLVLFSFTIG LTQLYDKGYT SKEQ------
TRPC4 Hsap_NP_003297.1      SLFTANSHLG PLQISLGRML LDILKFLFIY CLVLLAFANG LNQLYFY-YE ETKG------
TRPC4_Mmus_NP_058680.1      SLFTANSHLG PLQISLGRML LDILKFLFIY CLVLLAFANG LNQLYFY-YE ETKG------
TRP_Spur XP_793901          CYTVMNRHVG PMQISLGNMM FDICKFLLIF CFVWFAFSLG MNQLYGY-YS YITR------
TRP_Cint XP_002124651.2      YILEVNEKFG PLQISLLSTV GDIIKWSGIF FMIFGAFLLG LFNLYSY-YR EPEA------
TRP_Skow XP_002733765.1      YLFQANPYLG PLQISLGCML IDIAKFLFIF FLVLLSFACG LNQLYWY-YG SSSP------
TRP_Lflor__emb|CAA11261.1   YIFTVNAHLG PLQISLGRMV SDIVKFISVY FLVLFSFACG LNHLYWY-YA ALRA------
TRP_Bflo XP_002611405.1      FLFTSNSHLG PLQISLGRMG FDIVKVLFIY FLVLFAFANG LNQLYFP-YN NMNGSLQTDG
TRP4_Sman XP_002576849.1    YVFTVSPQLG PLQISLGRML HDIFRFFCVY FLVLVAFAFG FNQLYWF-YA KNRA------
TRP_Nvec4 XP_001640409      NAVQVNSTLG PLQLSLVKMV KDILKFLLLF FLIYLAFVVA LRRVYSQ-YV WAGK------
TRP_Nvec1 XP_001637374.1    YLCQISRYLG LLQLSLSRMV RVIFQFAFIS CVVLWSFSVA MTMLYMSSFE AYQL------
Cca7050                     QYLRVHRVIG PIQKSYGKVV QNMACFVVIL FIYLMAFASG LVNIYGA--- ----------
Cca21784                    RYLSWHPVIG PVQRAISKIV EEILLFLVIL MVFLTAFAAG ITNVYRG--- ----------
Ava16635_4                  QFLVIIKDSG PFVYMVFRML KNLGSFLILA LIFLLAYGVA SQTILYPNLP RDDP------
Ava9904_1                   QYLIMSAYFG VIVLIIFALA EEVVYFVFVL LLSMIGFGAA MQAVVASDTL YEDF------
Ava6620_1                   QYVLVIRGLG PLIYTCYIAF KRMLKFIVIF VLVSLGFGII EVTLTSTNQ- ----------
```

```
TRPC1_Hsap_NP_003295.1       --KDCVGIF- ---------- --CEQQSN-D TFHSFI-GTC FALFWYIFSL AHVAIFVTRF
TRPC1_Mmus_NP_035773.1       --KDCVGIF- ---------- --CEQQSN-D TFHSFI-GTC FALFWYIFSL AHVAIFVTRF
TRPC4_Hsap_NP_003297.1       --LTCKGIR- ---------- --CEKQN--N AFSTLF-ETL QSLFWSIFGL --INLYVTNV
TRPC4_Mmus_NP_058680.1       --LSCKGIR- ---------- --CEKQN--N AFSTLF-ETL QSLFWSIFGL --INLYVTNV
TRP_Spur_XP_793901           --VICDTNN- ---------- --PMEEHCKQ PFETVQ-YTM STLFWALFGL --PEMNIVDI
TRP_Cint_XP_002124651.2      ---------- ---------- ------KTTK AFTTIE-ETF ITLFWSMFGL --ADYRGVEV
TRP_Skow_XP_002733765.1      --VHSQDAD- ---------- --CPSNSNPD AFLSLD-RSM QTLFWALFGL --VGTDVIKL
TRP_Lflor__emb|CAA11261.1    --EECE---- ------AGIT HSCDIKY--R SFANLF-EIL QSLYWAVYGL --VDLEHAHL
TRP_Bflo_XP_002611405.1      NDYYCYGVR- ---------- --CHDQN--N AFSSLW-ETL QALFWSLFGL --VNLYVTKV
TRP4_Sman_XP_002576849.1     --RNCKNVHF TLEEGQKDVY DYCITRG--T YFTNLF-EIV QSLYWSAYGL --IDLTSLNL
TRP_Nvec4_XP_001640409       -TPHTISLY- ---------- ---------S VFCSVQ-GSM RLVFWAIFDK --TELEEFNT
TRP_Nvec1_XP_001637374.1     -PRPVKNVTS DIDAILD--- ---------K GYDNLL-STM VTLMWASLDM --VGLDTLNV
Cca7050                      -------TGF AVNKTEANNT QNCPS----E GFESMQ-SSA QSLFWSLFGL --LDVGDLSN
Cca21784                     -------TRF VFNETVRANA TNCPA----A DFSGVW-ISG ASLFWALFGL --LERQDMEN
Ava16635_4                   ---------- ---------- ---------N SYAFVFGNIF FRPYFQVFGE --FFLEHIQA
Ava9904_1                    ---------- ---------- ---------Q AQFLLF-TIF FRPYWQLFGE --FFLPQLGT
Ava6620_1                    ---------- ---------- ---------N GWNLLR-ILL FSPYYQIFGE --FGLANIRT

TRPC1_Hsap_NP_003295.1       -----SY--- -GEELQSF-- ---------- ---------- ---------- -----VGAVI
TRPC1_Mmus_NP_035773.1       -----SY--- -GEELQSF-- ---------- ---------- ---------- -----VGAVI
TRPC4_Hsap_NP_003297.1       -----KA--- -QHEFTEF-- ---------- ---------- ---------- -----VGATM
TRPC4_Mmus_NP_058680.1       -----KA--- -QHEFTEF-- ---------- ---------- ---------- -----VGATM
TRP_Spur_XP_793901           --------RG VDHEFTET-- ---------- ---------- ---------- -----VGLML
TRP_Cint_XP_002124651.2      -----KY--- -DHQLTRI-- ---------- ---------- ---------- -----VGYQL
TRP_Skow_XP_002733765.1      -----PTQ-- -GHNYTEF-- ---------- ---------- ---------- -----VGEIL
TRP_Lflor__emb|CAA11261.1    -----DE--- -PHKLTEL-- ---------- ---------- ---------- -----IGKLM
TRP_Bflo_XP_002611405.1      -----ENAQG YKHAFTEF-- ---------- ---------- ---------- -----VGTWM
TRP4_Sman_XP_002576849.1     -----EY--- -PHAFTEF-- ---------- ---------- ---------- -----VGKLT
TRP_Nvec4_XP_001640409       -----DP--S FPTKITQS-- ---------- ---------- ---------- -----TGEVL
TRP_Nvec1_XP_001637374.1     -----FK--- -KQSLIQF-- ---------- ---------- ---------- -----WTAAL
Cca7050                      ---------- -CTHEEAT-- ---------- ---------- ---------- -----AGKIV
Cca21784                     ---------- -CSGAEET-- ---------- ---------- ---------- -----AGQIT
Ava16635_4                   NAFTNGSSAM FGNSFPEVFV SSNF------ ---------- ---------- -----FVYFF
Ava9904_1                    D-INGGSGVI SGNLTTSILF SGSTKTSNGT VHSCVAQSSL QTTNPLLEFD YTRHPLPYFI
Ava6620_1                    --FSISFQNG NGTGEPQFFE STTF------ ---------- ---------- -----IAYVF

TRPC1_Hsap_NP_003295.1       VGTYNVVVVI VLTKLLVAML HKSFQLIANH EDKEWKFARA KLWLSYFDDK -CTLPPPFNI
TRPC1_Mmus_NP_035773.1       VGTYNVVVVI VLTKLLVAML HKSFQLIANH EDKEWKFARA KLWLSYFDDK -CTLPPPFNI
TRPC4_Hsap_NP_003297.1       FGTYNVISLV VLLNMLIAMM NNSYQLIADH ADIEWKFART KLWMSYFEEG -GTLPTPFNV
TRPC4_Mmus_NP_058680.1       FGTYNVISLV VLLNMLIAMM NNSYQLIADH ADIEWKFART KLWMSYFEEG -GTLPTPFNV
TRP_Spur_XP_793901           YAAYHVIAIV VLLNVLIAMM SNTYTRIEDD SEVQWKFSRS KLWMSYFAGR -GSLPAPFNV
TRP_Cint_XP_002124651.2      YGAYNIITVI IMLNMLIAMI NRSYSDVEVD SDLEWKFARA KLWLSYFDPG -STLPVPFNL
TRP_Skow_XP_002733765.1      FACYHVVAII VLLNMLIAMM SKSYQEIEDH ADREWKFART KLWMSYFDEG -STLPPPFNL
TRP_Lflor__emb|CAA11261.1    FGSYSMIAII ILLNLLIAMM SNSYQLIYSQ ADEEWKFARS RLWISYFAEG -ATVPPPFNI
TRP_Bflo_XP_002611405.1      FGAYNVIALI VLLNMLIAMM NNSYQHIADH ADTEWKFART KLWLSYFEEG -GTLPPPFNI
TRP4_Sman_XP_002576849.1     FGTYSYIAFI VLLNMLIAMM NNSYEQIVGQ VDIEWKFARS KLWISYFGEV -SKLRRGRE-
TRP_Nvec4_XP_001640409       FALFNIASIL VAINMLIAMM SNSFQKVADS EDFLWKFSRT RMWMEYIIKG -SVLPAPFSV
TRP_Nvec1_XP_001637374.1     FTLYHAASMV VLLNMLIAMM SNSYQQVEDN IETEYKFART QLWTDYIGDA VPTLPPPLNL
Cca7050                      FGLWLLQAMI ILLNMLIALV TNKFDEFQSN ADTEWKASFG ATVID-VQKS -QRYPIPFNL
Cca21784                     MAVWLVSSVV ILLNMLIAVV TNKFDEVEEN ADTEWKFTRA ELLMQ-VKHT -HSIPIPFNI
Ava16635_4                   LVIWLILSNI LLINIIIAKF NNTFVEIESN AAIYWKYKFF NS-VKEFRDK -PVLPPPFNV
Ava9904_1                    LALWLIFSNV LLVNLLIAKF NNIYLIVEAK SSILWKFTRY SA-IEEFRLK -PTFPPPFIF
Ava6620_1                    LIILQLIANV LLLNLMIAYF TKIFNEISEN ADSIYLTQFL EV-VDEYQRK -SLFPPPFNL

TRPC1_Hsap_NP_003295.1       IPSPKTI--- -CYMISSL-- -SKWICSHTS KGKVKRQNSL K-----EWRN LKQ-------
TRPC1_Mmus_NP_035773.1       IPSPKTI--- -CYMISSL-- -SKWICSHTS KGKVKRQNSL K-----EWRN LKQ-------
TRPC4_Hsap_NP_003297.1       IPSPKSL--- -WYLIKWI-- -WTHLCKKKM RRKPESFGTI GVRTQHRRAA DNL-------
TRPC4_Mmus_NP_058680.1       IPSPKSL--- -WYLVKWI-- -WTHLCKKKM RRKPESFGTI G-----RRAA DNL-------
TRP_Spur_XP_793901           LPTPKSM--- -LYLFRWL-- -RGKLGSEKH RQEIRDRNKA KME------- ----------
TRP_Cint_XP_002124651.2      LTLPQCIHDG VIYIFKLT-- -RKELYKKTR RASSSQRR-- ------DHHR KKTELRK---
TRP_Skow_XP_002733765.1      IISPKSM--- -VCFFKAI-- -QRIMCGSCE KTKSRPQRQS L-----EGTI KKTHLNSDGV
TRP_Lflor__emb|CAA11261.1    IPSPKSF--- -TNMMTLF-- -KSLILSHTA EQKQAKWTNV ------RSTV KNIN------
TRP_Bflo_XP_002611405.1      VPSPKFF--- -WYTIMWVKN SCSEICNFR- KKQLNRMRSI G-----KMTA VKL-------
TRP4_Sman_XP_002576849.1     ---------- -AYQLSEI-- -QEMVRGKKS RESIKRTNIS INE------- ----------
TRP_Nvec4_XP_001640409       LPSIASI--- -INFF----- ---------- ---------- ---------- ----------
TRP_Nvec1_XP_001637374.1     LPTPKYI--- -VRLL----- ---------- ---------- ---------- ----------
Cca7050                      LHVTVDI--- -LEWL----- ---------- ---------- ---------- ----------
Cca21784                     LNEIVDL--- -LEFLFRK-- -RRPNLEAAM WMSLIPLTTV TPE------- ----------
Ava16635_4                   IVILYRV--- -VKRLYLL-- CNRYRVNSTV RSKDRGNNE- ---------- ----------
Ava9904_1                    LSHIRKL--- -VKFLYGK-- CAVRIFRKNI YKEDMKNFH- ---------- ----------
Ava6620_1                    LVYLFRT--- -FGICKTV-- -CQDFFKEHE ETMEDEFQRD THFNRAKVYP VYFGHHNENG

TRPC1_Hsap_NP_003295.1       -----KRDEN YQKVMCCLVH RYLTSMRQKM QSTDQATVEN LNELRQDLSK FRNEIRDLLG
TRPC1_Mmus_NP_035773.1       -----KRDEN YQKVMCCLVH RYLTSMRQKM QSTDQATVEN LNELRQDLSK FRNEIRDLLG
TRPC4_Hsap_NP_003297.1       -----RRHHQ YQEVMRNLVK RYVAAMIRDA KTEEGLTEEN FKELKQDISS FRFEVLGLL-
TRPC4_Mmus_NP_058680.1       -----RRHHQ YQEVMRNLVK RYVAAMIREA KTEEGLTEEN VKELKQDISS FRFEVLGLL-
```

```
TRP_Spur XP_793901            -----EKEQQ YREIMGKLVK RYIFDAKRDE --DENNQEQW VNRLKQDISG FKYEMFEALT
TRP_Cint XP_002124651.2       -----RMSMH HINVMTRIVK RYVLRAQVEE AGGSNTSEAD LQEIKHDISS LRYEVIGGLT
TRP_Skow_XP_002733765.1       IGPPLQENIK YQDVMKRLVS RYIHQQKAEQ K-QDGVNEDD LNEIKQDISS LRYELRADR-
TRP_Lflor__emb|CAA11261.1     -----KREIR YQYVIRNLIS RYIMNKQRPQ K-DEMVSQDD INELKQDIST FRFELLTILS
TRP_Bflo_XP_002611405.1       -----RKDMA YKDVIQNLVR RYIANEIRDR ESSEGLCEDD INELKQDISS FRYEMLALL-
TRP4_Sman_XP_002576849.1      -----NKSTQ SKPLFSDIKQ ----PVHRKI TTSTGINKSD FNKTLLPSST SIGGIIGPV-
TRP_Nvec4_XP_001640409        ---------- ---------- ---------- ---------- ---------- ----------
TRP_Nvec1_XP_001637374.1      ---------- ---------- ---------- ---------- ---------- ----------
Cca7050                       ---------- ---------- ---------- ---------- ---------- ----------
Cca21784                      -----RRFEA QKELLQTLKR K-----EERE GMESPSTKSD MRKVSEQLHE LK--------
Ava16635_4                    -----HKEKE KEKILFDFEK RCVLACNFNL EQTNEL---- --NLDKRITE VDNRLKNT--
Ava9904_1                     -----RKNAE RWKWTKDIQQ QGLNRWESKR LSEQEE---- --SVRNLLSN VETKLEDVYR
Ava6620_1                     FADEYWRNRE KIRIKGNLQ- --LKKRQKQV EYLTQKSQEQ SLHRKYTVKY FYFNFTGVRR

TRPC1_Hsap_NP_003295.1        FRTSKYAMFY --PRN----- ---------- ---------- ---------- ----------
TRPC1_Mmus_NP_035773.1        FRTSKYAMFY --PRN----- ---------- ---------- ---------- ----------
TRPC4 Hsap_NP_003297.1        -RGSKLSTIQ --SANASKES SNSADSDEKS DSEGNS---- --------KD KKKNFSLFDL
TRPC4_Mmus_NP_058680.1        -RGSKLSTIQ --SANA---- ASSADSDEKS QSEGNG---- --------KD KRKNLSLFDL
TRP_Spur XP_793901            GMDKKMSEME QRIEDGGVKE PGTQMFHKME DVVKRPLYQP D-------SM QSVISGCSDL
TRP_Cint XP_002124651.2       RWDSKIEELF NSLEKQKTEE GQIQISTAVA QIERKS---- --------KN ERLAPPVLDA
TRP_Skow_XP_002733765.1       -KKEQMRGTS QIENVKHEII GELRGISGAP ENESTTIVGA PRRSLSTVNP PSFISQSFDK
TRP_Lflor__emb|CAA11261.1     DNGFETPTVH Q-AKTSSRLD RMWKNLSAAT EGQTETLMEE AGLDEE---- ----------
TRP_Bflo_XP_002611405.1       -KSDGGEGGP --DSLQDERP SRRKRRTGKY SLEALS---- --------QP RSRSMNNLDR
TRP4_Sman_XP_002576849.1      -DNPAFENEE --VIVNKTSI SSSVDLDGKK QGKSTTVLDQ KTSVSDVTSQ QLNPNFIPCT
TRP_Nvec4_XP_001640409        ---------- ---------- ---------- ---------- ---------- ----------
TRP_Nvec1_XP_001637374.1      ---------- ---------- ---------- ---------- ---------- ----------
Cca7050                       ---------- ---------- ---------- ---------- ---------- ----------
Cca21784                      ---------- ---------- ---------- ---------- ---------- ----------
Ava16635_4                    ---------- --QHLIVTLT NKVDSLVRST GSF--E---- --------SE QDFFSLQGDT
Ava9904_1                     NCHTLLDHMG SDEPLLTMMT SEIREVRRQL ADFGVH---- --------SF QNYLLILHLV
Ava6620_1                     KGVKSVNLFF CAYPFNLX-- ---------- ---------- ---------- ----------

TRPC1_Hsap_NP_003295.1        ---------- ---------- ---------- ---------- ---------- ----------
TRPC1_Mmus_NP_035773.1        ---------- ---------- ---------- ---------- ---------- ----------
TRPC4 Hsap_NP_003297.1        TTLIHPRSAA IASERHNISN --GSALVVQE PPREKQRKVN FVT------- ----------
TRPC4_Mmus_NP_058680.1        TTLIHPRSAA IASERHNLSN --GSALVVQE PPREKQRKVN FVA------- ----------
TRP_Spur XP_793901            MTAASKDLMN SKGSNNADNS PILDDGLSDP STKNRRQPKS TSMPCNIAGY LYPPDWEKNE
TRP_Cint XP_002124651.2       TEINK----- ---------- ---------- ---------- ---------- ----------
TRP_Skow_XP_002733765.1       SELESLKDDL IHTIRSEIKE ELQESLRR-- ---------- ---------- ----------
TRP_Lflor__emb|CAA11261.1     ---------- ---------- ---------- ---------- ---------- ----------
TRP_Bflo_XP_002611405.1       LEE-HPAVDE TDSAKACLSR PLAAAAVRSV FPVLEQRQRN -VM------- ----------
TRP4_Sman_XP_002576849.1      SAVVCEETIT NNTDQNTVEK QLQQSKLKQP PSQLSIETKT PSM------- ----------
TRP_Nvec4_XP_001640409        ---------- ---------- ---------- ---------- ---------- ----------
TRP_Nvec1_XP_001637374.1      ---------- ---------- ---------- ---------- ---------- ----------
Cca7050                       ---------- ---------- ---------- ---------- ---------- ----------
Cca21784                      ---------- ---------- ---------- ---------- ---------- ----------
Ava16635_4                    MGSMHGRRIL IRYFNNVSIL QLFISPLSYT TSPVLYQERL NIF------- ------FILC
Ava9904_1                     I--KHITRST HFKITGLLLL PLYIYTVYSC TSPLCYFPKT FIFVVTIQYF PINICLFLFC
Ava6620_1                     ---------- ---------- ---------- ---------- ---------- ----------

TRPC1_Hsap_NP_003295.1        ---------- ---------- ---------- ---------- ---------- ----------
TRPC1_Mmus_NP_035773.1        ---------- ---------- ---------- ---------- ---------- ----------
TRPC4 Hsap_NP_003297.1        ---------- --DIKNFGLF HRRSKQNAAE QNANQIFSVS EEVARQQAAG PLERNIQLES
TRPC4_Mmus_NP_058680.1        ---------- --DIKNFGLF HRRSKQNAAE QNANQIFSVS EEITRQQAAG ALERNIELES
TRP_Spur XP_793901            PILFMDEDPD VPILGGPPRK ESKKRRFSAN HMMNHIGSRH LKRKKSHTKN TTRV------
TRP_Cint XP_002124651.2       ---------- ---------- ---------- ---------- ---------- ----------
TRP_Skow_XP_002733765.1       ---------- ---------- --ALNSAAQE SSVNGLLPTS SDLYHTHLYT QL--------
TRP_Lflor__emb|CAA11261.1     ---------- ---------- ---------- ---------- ---------- ----------
TRP_Bflo_XP_002611405.1       ---------N GQVAGAADRT WSRGNPGGGL QDGNPLLLQT ERVS---SMG RISRTFVTLA
TRP4_Sman_XP_002576849.1      ---NIIKNDK MPNLKNSTFT NETRSQTVKQ DHADKIESVE NEATSQLFSD RKSTDHKTIE
TRP_Nvec4_XP_001640409        ---------- ---------- ---------- ---------- ---------- ----------
TRP_Nvec1_XP_001637374.1      ---------- ---------- ---------- ---------- ---------- ----------
Cca7050                       ---------- ---------- ---------- ---------- ---------- ----------
Cca21784                      ---------- ---------- ---------- ---------- ---------- ----------
Ava16635_4                    MKVSEDLVVL SR-------- ---------- ---------- ---------- ----------
Ava9904_1                     ---SSRMIYD SMLCCV---- ---------- ---------- ---------- ----------
Ava6620_1                     ---------- ---------- ---------- ---------- ---------- ----------

TRPC1_Hsap_NP_003295.1        ---------- ---------- ---------- ---------- ---------- ----------
TRPC1_Mmus_NP_035773.1        ---------- ---------- ---------- ---------- ---------- ----------
TRPC4 Hsap_NP_003297.1        RGLASRGDLS IPGLSEQCVL VDHRERNTDT LGLQVGKRVC P-FKSEKVVV EDTVPIIPKE
TRPC4_Mmus_NP_058680.1        KGLASRGDRS IPGLNEQCVL VDHRERNTDT LGLQVGKRVC STFKSEKVVV EDTVPIIPKE
TRP_Spur XP_793901            ---------- ---------- ---------- ---------- ---------- ----------
TRP_Cint XP_002124651.2       ---------- ---------- ---------- ---------- ---------- ----------
TRP_Skow_XP_002733765.1       ---------- ---------- ---------- ---------- ---------- ----------
TRP_Lflor__emb|CAA11261.1     ---------- ---------- ---------- ---------- ---------- ----------
TRP_Bflo_XP_002611405.1       SPVLGRRRRQ QRKGLDE--- -----EEDTE LGAMSRTNRN FANENDMGSV FTCAPITEID
```

```
TRP4_Sman_XP_002576849.1   HCKLNKISHH PMDECSQMKN QRSDLV---- ---------- ---------- ----------
TRP_Nvec4_XP_001640409     ---------- ---------- ---------- ---------- ---------- ----------
TRP_Nvec1_XP_001637374.1   ---------- ---------- ---------- ---------- ---------- ----------
Cca7050                    ---------- ---------- ---------- ---------- ---------- ----------
Cca21784                   ---------- ---------- ---------- ---------- ---------- ----------
Ava16635_4                 ---------- ---------- ---------- ---------- ---------- ----------
Ava9904_1                  ---------- ---------- ---------- ---------- ---------- ----------
Ava6620_1                  ---------- ---------- ---------- ---------- ---------- ----------

TRPC1_Hsap_NP_003295.1     ---------- ---------- ----------
TRPC1_Mmus_NP_035773.1     ---------- ---------- ----------
TRPC4_Hsap_NP_003297.1     KHAKEEDSSI DYDLNLPDTV THEDYVTTRL
TRPC4_Mmus_NP_058680.1     KHAHEEDSSI DYDLSPTDTA AHEDYVTTRL
TRP_Spur_XP_793901         ---------- ---------- ----------
TRP_Cint_XP_002124651.2    ---------- ---------- ----------
TRP_Skow_XP_002733765.1    ---------- ---------- ----------
TRP_Lflor__emb|CAA11261.1  ---------- ---------- ----------
TRP_Bflo_XP_002611405.1    DSAEEEASHD EENSSSESAS RHE-----VV
TRP4_Sman_XP_002576849.1   ---------- ---------- ----------
TRP_Nvec4_XP_001640409     ---------- ---------- ----------
TRP_Nvec1_XP_001637374.1   ---------- ---------- ----------
Cca7050                    ---------- ---------- ----------
Cca21784                   ---------- ---------- ----------
Ava16635_4                 ---------- ---------- ----------
Ava9904_1                  ---------- ---------- ----------
Ava6620_1                  ---------- ---------- ----------
```
